# Supplementary material for: Exploration of How Uncertainty Tolerance, Emotion Regulation, and Hope Are Linked and Influenced in People with Chronic Low Back Pain: A Worked Example of a Social Constructivist Meta-Ethnography Study
Source: Behav Sci (Basel). 2025 Oct 15;15(10):1399. doi: 10.3390/bs15101399 (PMC12561381; doi:10.3390/bs15101399)

Title:
Exploration of how uncertainty tolerance, emotion regulation, and hope are linked and influenced in people with chronic low back pain: a worked example of a social constructivist meta-ethnography study.

Supplementary File A: Audit Trail.

Contents:

	Information	Page numbers:
**Step 1**<br>**The Primary Systematic Search: Hope**	Databases searched: MEDLINE, CINAHL Plus, Pubmed, AMED, PEDro, SportDiscus, Google Scholar, ScienceDirect, Hope-Lit Database. – Screenshots.<br>Search terms outlined.<br>Reasons for exclusions provided.<br>Undertaken by the main author (MSc dissertation)<br>January 2024	5-18
**Step 2**<br>**Immersive reading, coding and exploring how the studies relate.**	A table outlining the coding of the qualitative articles and how the theme of uncertainty came to light and became a new focus is explained.	19-23

**Step 3**<br>**Second systematic search: Uncertainty**	Databases searched: MEDLINE, CINAHL Plus, Pubmed, AMED, PEDro, SportDiscus, Google Scholar, ScienceDirect, Hope-Lit Database. – Screenshots.<br>Search terms outlined.<br>Reasons for exclusions provided.<br>Undertaken by the main author (MSc dissertation)<br>January 2024	23-35
**Repeat Step 2:**<br>**Immersive reading, coding and exploring how the studies relate.**	A table outlining the coding of the two qualitative articles on uncertainty in people with CLBP.	36-40
**Step 4:**<br>**Iterative process of idea generation**	The following tables consider the different concepts to ensure alignment with thoughts and the process of testing information.<br>- Summary Table: Comparison of Uncertainty, and Intolerance of Uncertainty<br>- Summary Table: Frameworks for Intolerance of Uncertainty (IU) and identifying supportive comments and concepts<br>- Summary Table: Emotional Regulation Main Theories<br>- Summary Table: Comparison of Hope Theories in Health and Illness<br>- Summary Table: Theories of Hopelessness in Psychology	41-51

	A table outlining the first and second research question and its line of argument synthesis<br>A table outlining the third research question and its line of argument synthesis<br>A table outlining the fourth research question and its line of argument synthesis	51-60
**Step 5: Expression of the theory and testing the model.**	Model versions 1-7b and reasons for modifications	61-70
**Peer review process: Feedback and amendments**		71-76
**Step 6**<br>**Repeat all three systematic searches**	**Three blind systematic searches using COVIDENCE Software**<br>- Hope<br>- Uncertainty<br>- Emotion regulation	77-81
**Repeat Step 2**<br>**Immersive reading, coding and exploring how the studies relate.**	- Review of qualitative literature<br>- Coding of articles on hope and uncertainty<br>- Generation of themes<br>*Strengthened previous theory	82-90
**Repeat Step 4:**		91-95

**Iterative process of idea generation**		
**Repeat Step 5: Modifications to the model.**	Model versions 8 & 9 with changes/justifications	96-97
**FINAL MODEL**		98

STEP 1: The primary systematic search

Figure 1. A table outlining the databases and search terms for the first systematic search.

**Database**	**Search Terms**
MEDLINE	'Hope' OR 'Hopelessness' OR 'Hope scale' NOT 'optimism'<br>AND 'Chronic low back pain' OR 'non-specific low back pain' OR 'persistent low back pain' AND 'pain management' OR 'pain reduction' OR 'quality of life'
CINAHL	'Hope' OR 'Hopelessness' OR 'Hope scale' NOT 'optimism'<br>AND 'Chronic low back pain' OR 'non-specific low back pain' OR 'persistent low back pain' AND 'pain management' OR 'pain reduction' OR 'quality of life'
PubMed	(Chronic low back pain OR non-specific low back pain OR persistent low back pain) AND (Hope' OR 'Hopelessness' OR 'Hope scale' NOT 'optimism') AND ('pain management' OR 'pain reduction' OR 'quality of life')
AMED	'Hope' OR 'Hopelessness' OR 'Hope scale' NOT 'optimism'<br>AND 'Chronic low back pain OR non-specific low back pain OR persistent low back pain'.
PEDro	'Chronic pain' 'hope'<br>'Chronic low back pain' 'hope'<br>'Persistent low back pain' 'hope'

	Low back * 'hope'<br>NB: It automatically uses 'and' between words. Also use the askaris (e.g. hope* - will cover hopelessness etc.)
SPORTDiscus	'Hope' OR 'Hopelessness' OR 'Hope scale' NOT 'optimism'<br>AND 'Chronic low back pain' OR 'non-specific low back pain' OR 'persistent low back pain' AND 'pain management' OR 'pain reduction' OR 'quality of life'
Google Scholar	allintitle: hope "chronic pain".
ScienceDirect	(hope OR hopelessness OR hope scale NOT optimism) AND (chronic low back pain OR persistent low back pain OR non-specific low back pain) in title and abstract.
Hope-Lit database. The University of Alberta.	Searched through all articles in the 'Chronic Pain' section.

Limitations:

- Search mode: Boolean/Phrase
- Publication date: 2003-2024
- Language: English
- Search field: Abstract.

Inclusion criteria:

- Population: adults (18-70yrs) with chronic low back pain (>3months)
- The use of an outcome measure of hope, or discussed the concept of hope from the perspective of the individual with CLBP in the results section of the abstract.

<u>STEP 1 (continued):</u> Search Process

Figure 2. NHS Knowledge and library hub search strategy screenshot.

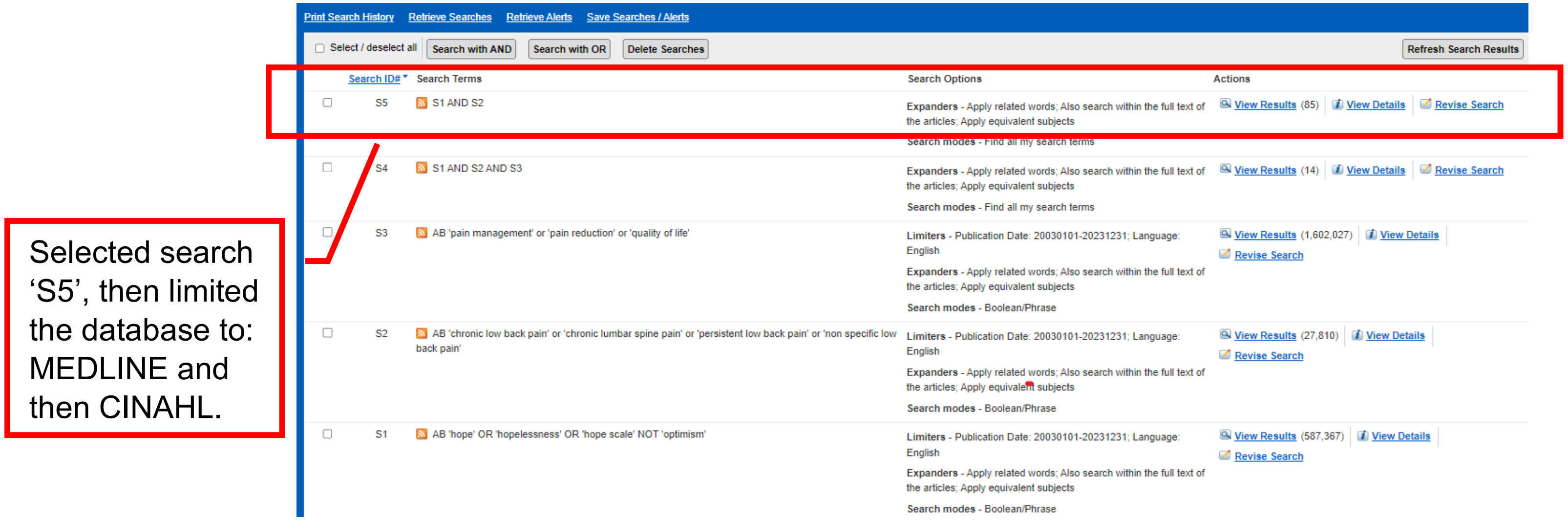

Figure 3. A table outlining the search results for MEDLINE and CINAHL.

**Database**	No. of articles	Met eligibility criteria	Articles:
MEDLINE	18	1	**Included:**<br>- Toye, F. & Barker, K. (2012). I can’t see any reason for stopping doing anything, but I might have to do it differently’ – restoring hope to patients with persistent non-specific low back pain – a qualitative study. *Disability & Rehabilitation, 34* (11), pp. 894-903.

CINAHL	13	1	**Included:**<br>- Toye, F. & Barker, K. (2012). I can't see any reason for stopping doing anything, but I might have to do it differently' – restoring hope to patients with persistent non-specific low back pain – a qualitative study. *Disability & Rehabilitation, 34* (11), pp. 894-903.

STEP 1 (continued): **Search Process**

Figure 4. A screenshot of the AMED (available through OVID platform) search strategy:

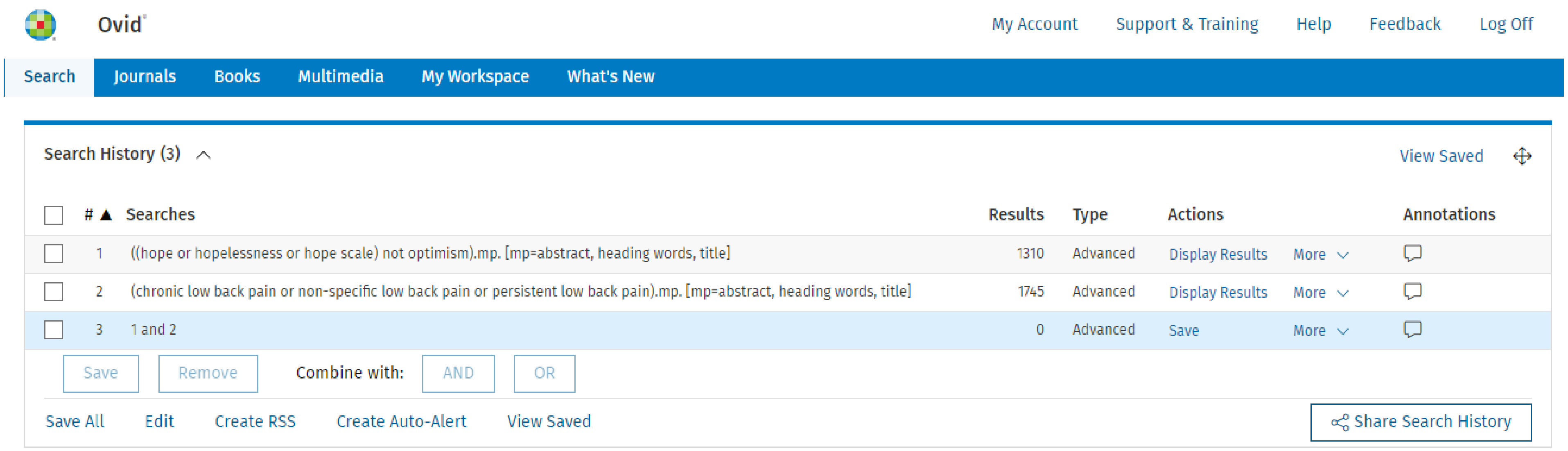

Figure 5. A table outlining the search results for AMED:

**Database**	No. of articles	Met eligibility criteria	Articles:
AMED	0	0	

<u>STEP 1 (continued):</u> Search Process

Figure 6. Screenshots (x4) of the PEDro search strategies.

Search 1:

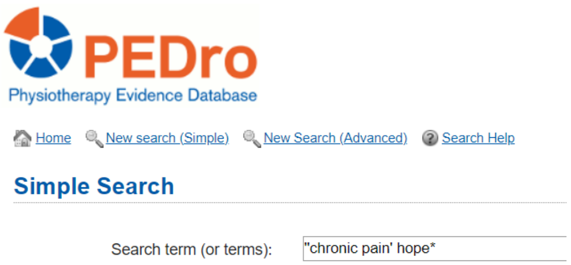

Search 2:

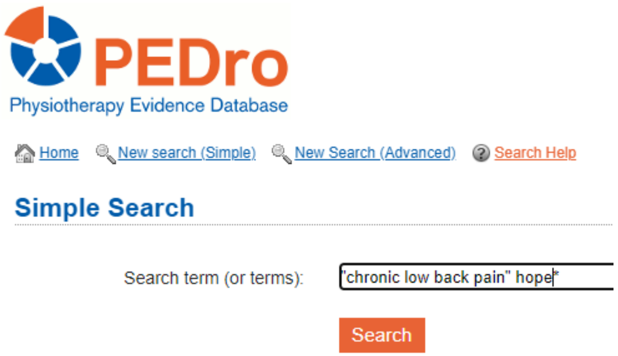

Search 3:

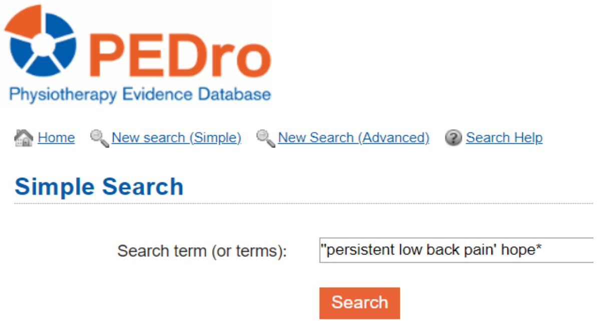

Search 4:

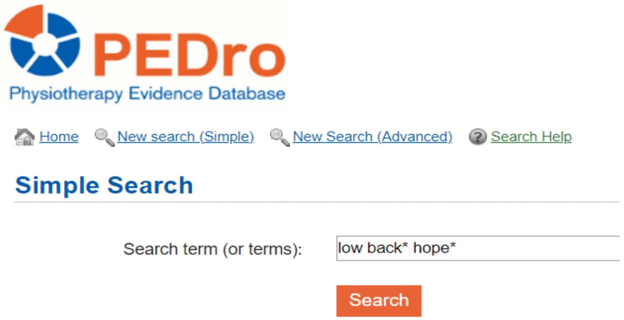

Figure 7. A table outlining the search results for PEDro

**Database**	No. of articles	Met eligibility criteria	Articles:

PEDro (Search 1)	19	0	
PEDro (Search 2)	6	0	
PEDro (Search 3)	1	0	
PEDro (Search 4)	17	0	

<u>STEP 1 (continued):</u> Search Process

Figure 8. A screenshot of the PudMed search strategy.

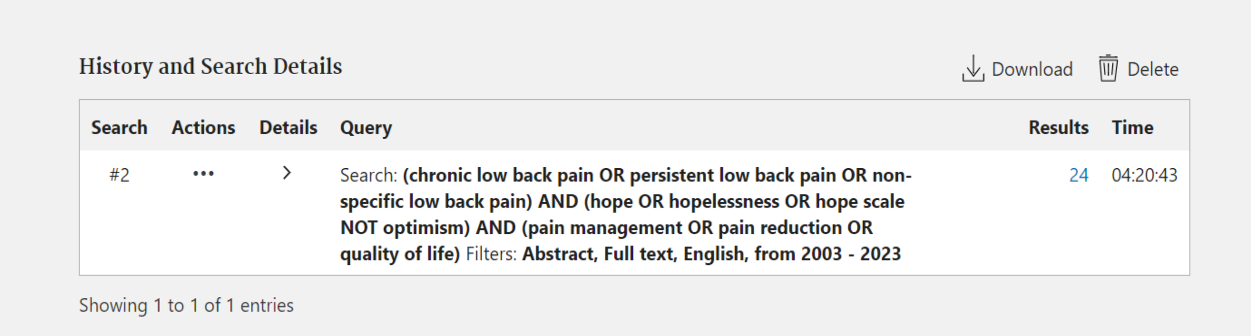

Figure 9. A table outlining the search results for PubMed

**Database**	No. of articles	Met eligibility criteria	Articles:
PubMed	24	2	Toye, F. and Barker, K. (2012). I can't see any reason for stopping doing anything, but I might have to do it differently' – restoring hope to patients with persistent non-specific low back pain – a qualitative study. *Disability & Rehabilitation, 34* (11), pp. 894-903.

			Corbett, M., Foster, N. and Ong, B. (2007) 'Living with low back pain - Stories of hope and despair', *Social science and Medicine*, 65 (8) pp. 1584-1594. doi:10.1016/j.socscimed.2007.06.008

<u>STEP 1 (continued):</u> Search Process

Figure 10. A screenshot of the SPORTDiscus search strategy.

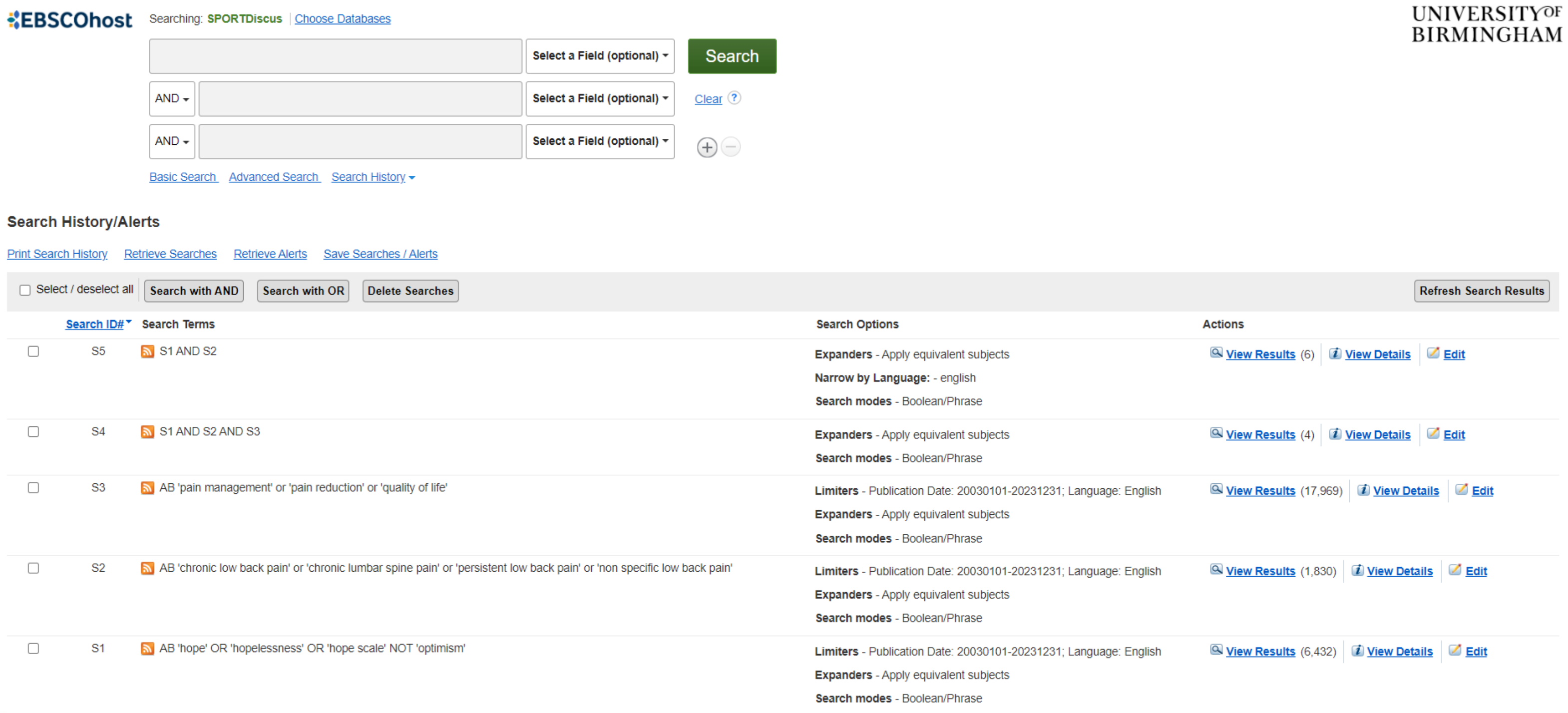

Figure 11. A table outlining the search results for SPORTDiscus.

**Database**	No. of articles	Met eligibility criteria	Articles:
SPORTDiscus	6	1	Toye, F. and Barker, K. (2012). I can't see any reason for stopping doing anything, but I might have to do it differently' – restoring hope to patients with persistent non-specific low back pain – a qualitative study. *Disability & Rehabilitation, 34* (11), pp. 894-903.

<u>STEP 1 (continued):</u> Search Process

Figure 12. A screenshot of the first Google Scholar search strategy (NB: Unable to limit language)

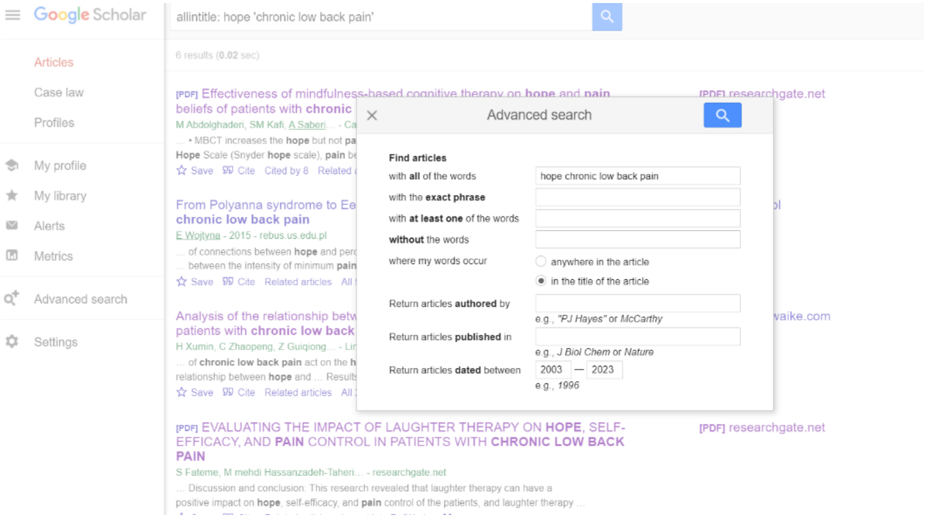

Figure 13. A table outlining the results for Google Scholar (search 1).

**Electronic Database**	No. of articles	Met eligibility criteria	Articles:
GoogleScholar Search 1	6	3	**Accepted:**

			• Wojtyna, E., Palt, L. and Popiolek, K. (2015) ‘From Polyanna syndrome to Eeyore’s Corner? Hope and pain in patients with chronic low back pain’, *Polish Psychological Bulletin, 46 (1) pp. 96-103.* doi:10.1515/ppb-2015-0013.<br>• Abdolghaderi, M. Kafi, S., Saberi, A. and Ariaporan, S. (2018) ‘ Effectiveness of Mindfulness-Based Cognitive Therapy on Hope and Pain Beliefs of Patients with Chronic Low Back Pain’, *Caspian Journal of Neurological Sciences*, 4 (1) pp. 18-23.<br>• Fateme, S., Hassanzadeh-Taheri, M., Fatemi, S., Moodi, H., Hosseini, M., Akbari, A., Doostabadi, M. (2017) ‘Evaluating the impact of laughter therapy on hope, self-efficacy, and pain control in patients with chronic low back pain’, *Pharmacophore*, 8 (6) e-1173663.<br><br>**Excluded:**<br>• Xumin et al (2015) ‘<u>Analysis of the relationship between hope and curative effect of spinal fusion in patients with chronic low back pain</u>’, *Lingnan Modern Clinics*.<br>**Reason: Not in English**

<u>STEP 1 (continued):</u> Search Process

Figure 14. A screenshot of the second Google Scholar search strategy (NB: Unable to limit language)

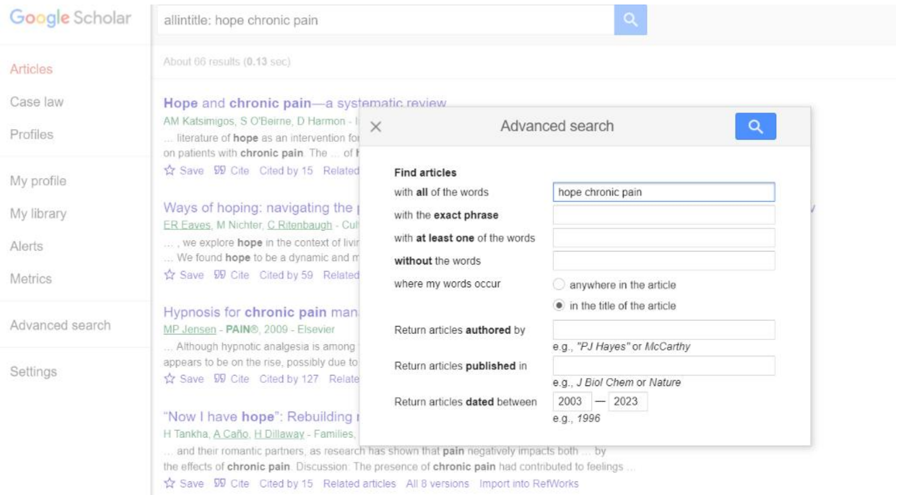

<u>STEP 1 (continued):</u> Search Process

Figure 15. A table outlining the results for Google Scholar (search 2).

**Electronic Database**	No. of articles	Met eligibility criteria	Articles:
GoogleScholar Search 2	66	4	**Accepted:**<br>• Abdolghaderi, M. Kafi, S., Saberi, A. and Ariaporan, S. (2018) 'Effectiveness of Mindfulness-Based Cognitive Therapy on Hope and Pain Beliefs of Patients with Chronic Low Back Pain', *Caspian Journal of Neurological Sciences*, 4 (1) pp. 18-23.<br>• Fateme, S., Hassanzadeh-Taheri, M., Fatemi, S., Moodi, H., Hosseini, M., Akbari, A., Doostabadi, M. (2017) 'Evaluating the impact of laughter therapy on hope, self-efficacy, and pain control in patients with chronic low back pain', *Pharmacophore*, 8 (6) e-1173663.<br>• Razavi, S., Aboalghasimi, S., Akbari, B. and Nadirinabi, B. (2022) 'The effectiveness of Cognitive Therapy on Hope and Pain management in Women with Chronic Pain', *Preventative Care in Nursing and Midwifery Journal,* 12 (2) pp. 18-23.<br>• Wojtyna, E., Palt, L., & Popiolek, K. (2015). 'From Polyanna syndrome to Eeyore's Corner? Hope and pain in patients with chronic low back pain'. *Polish Psychological Bulletin, 46* (1) pp. 96-103.<br><br>**Excluded:**<br>• Xumin et al (2015) '<u>Analysis of the relationship between hope and curative effect of spinal fusion in patients with chronic low back pain</u>', *Lingnan Modern Clinics*

			**Reason not included: Not in English. Surgical management.**<br>• Eaves, E., Nichter, M. and Ritenbaugh. C. (2016) '<u>Ways of hoping: navigating the paradox of hope and despair in chronic pain</u>', *Culture, medicine and Psychiatry'*, 40 pp. 35-58.<br>**Reason not included: Participants had temporomandibular disorders.**<br>• Katsimigos, O'Beirne, S. and Harmon, D. (2021) 'Hope and chronic pain - a systematic review', *Irish Journal of Medical Sciences*, 190 pp. 307-213.<br>**Reason not included: Covered a variety of chronic illnesses, not just LBP.**

<u>STEP 1 (continued):</u> Search Process

Figure 16. A screenshot of the ScienceDirect search strategy

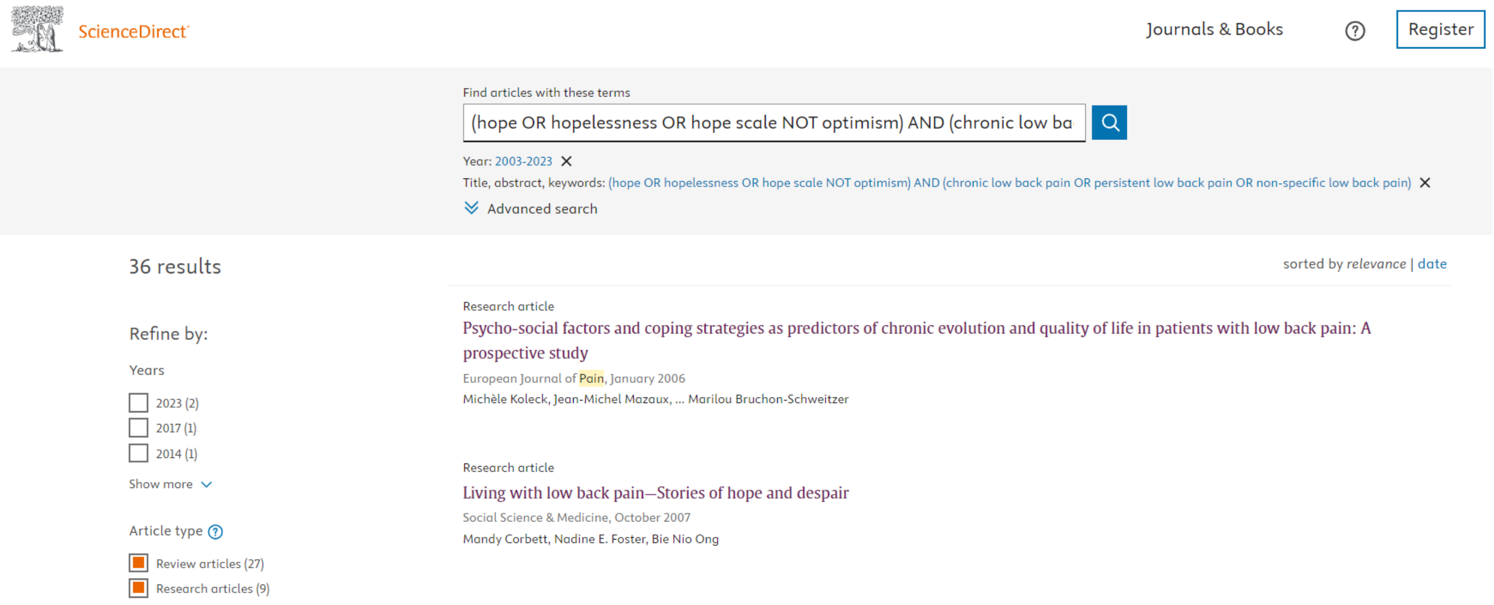

Figure 17. A table outlining the results for ScienceDirect.

**Electronic Database**	No. of articles	Met eligibility criteria	Articles:
ScienceDirect	36	1	Corbett, M., Foster, N. and Ong, B. (2007) 'Living with low back pain - Stories of hope and despair', *Social science and Medicine*, 65 (8) pp. 1584-1594. doi:10.1016/j.socscimed.2007.06.008

STEP 1 (continued): Search Process

Figure 18. The search results for the Hope-Lit Database, accessed via The University of Alberta website.

Selected Sub-category = Chronic pain

Search Results:

**Database**	No. of articles	Met eligibility criteria	Articles:
Hope-Lit Database	13	3	**Accepted:**<br>• Corbett, M., Foster, N. and Ong, B. (2007) 'Living with low back pain - Stories of hope and despair', *Social science and Medicine*, 65 (8) pp. 1584-1594. doi:10.1016/j.socscimed.2007.06.008<br>• Toye, F. and Barker, K. (2012). I can't see any reason for stopping doing anything, but I might have to do it differently' – restoring hope to patients with persistent non-specific low back pain – a qualitative study. *Disability & Rehabilitation, 34* (11) pp. 894-903.<br>• Wojtyna, E., Palt, L., & Popiolek, K. (2015). 'From Polyanna syndrome to Eeyore's Corner? Hope and pain in patients with chronic low back pain'. *Polish Psychological Bulletin, 46* (1) pp. 96-103.<br><br>**Excluded:**

			• Eaves, E. R, (2016). Ways of hoping: Navigating the paradox of hope and despair in chronic pain. *Culture, Medicine &Psychiatry, 40*(1), 35-58.<br>**Reason not included:** TMJ Disorders, not LBP<br>• Eaves, E. R., Ritenbaugh, C., Nichter, M., Hopkins, A. L., & Sherman, K. J. (2014). Modes of hoping: Understanding hope and expectation in the context of a clinical trial of complementary and alternative medicine for chronic pain. *Explore: The Journal of Science & Healing, 10*(4), 225-232.<br>**Reason not included:** TMJ Disorders, not LBP<br>• Edey, W., King, R. L., Larsen, D. J., & Stege, R. (2016). The "Being Hopeful in the Face of Chronic Pain" program: A counseling program for people experiencing chronic pain. *Journal for Specialists in Group Work, 41*(2), 161-187.<br>**Reason not included:** Chronic pain, multiple reasons – not solely LBP<br>• Howell, A. J., Jacobson, R. M., & Larsen, D. J. (2015). Enhanced psychological health among chronic pain clients engaged in hope-focused group counseling. *The Counseling Psychologist, 43* (4) pp. 586-613.<br>**Reason not included:** Chronic pain, multiple reasons/diagnoses – not solely LBP<br>• Jacobson, R. (2012). *Hope amidst the pain: Effects of a hope-focused intervention for individuals suffering from chronic pain*. Unpublished manuscript, Department of Psychology, Grant MacEwan University, Edmonton, AB, Canada.<br>**Reason not included:** unable to access full text<br>• Larsen, D. J., King, R. L., Stege, R., & Egeli, N. A. (2015). Hope in a strengths-based group activity for individuals with chronic pain. *Counselling Psychology Quarterly, 28* (2) pp. 175-199.<br>**Reason not included:** Chronic pain, multiple reasons/diagnoses – not solely LBP<br>• Säellfors, C., Fasth, A., & Hallberg, L.R.M. (2002). Oscillating between hope and despair--A qualitative study. *Child: Care, Health & Development*, 28 (6) pp. 495-505.<br>**Reason not included:** Children, not adults.<br>• Taylor, R. R. (2006). Instilling hope in people with chronic conditions. In Renee R. Taylor's *Cognitive behavioral therapy for chronic illness and disability* (pp. 172-183). New York, NY: Springer Science & Business Media, Inc. HSC<br>**Reason not included:** Chronic pain, multiple reasons/diagnoses – not solely LBP

			• Underwood, R. (2009). 'Hope in the face of chronic pain and mortality'. *Pastoral Psychology, 58* (5/6) pp. 655-665.<br>**Reason not included:** Not LBP<br>• Wright, M. A., Wren, A. A., Somers, T. J., Goetz, M. C., Fras, A. M., Huh, B. K., Rogers, L. L., & Keefe, F. J. (2011). 'Pain acceptance, hope, and optimism: Relationships to pain and adjustment in patients with chronic musculoskeletal pain'. *The Journal of Pain, 12* (11) pp. 1155-1162.<br>**Reason not included:** Chronic MSK pain, multiple reasons – not solely LBP.

<u>STEP 1 (continued):</u>

Figure. 19. A table summarising the articles which met the eligibility criteria following the first systematic search.

Database	Number of articles
MEDLINE	18
CINAHL	13
AMED	0
PEDro	43
PubMed	24
SPORTDiscus	6
Google Scholar	72
ScienceDirect	36
Hope-Lit database	13
**Total**	**225**
**Removed duplicates**	**47**
**Records screened**	**178**<br>(excluded 161 by title/abstract)
**Records sought for retrieval**	**16**<br>(1 not available full text)
**Reports excluded**	**10**

**Included**	**6**<br>(2 x qualitative and 4 x quantitative articles)

Qualitative articles (x2):

- Corbett, M., Foster, N. and Ong, B. (2007)
- Toye and Barker (2012)

Quantitative articles (x4)

- Abdolghaderi et al (2018)
- Fateme et al (2017)
- Razavi et al (2022)
- Wojtyna, E., Palt, L., & Popiolek, K. (2015).

<u>STEP 2:</u> Immersive reading, coding and exploring how the studies relate.

Figure. 20. A table outlining the coding of the two qualitative articles.

	**First order coding**<br>(Participant verbatim quotes):	**Second order coding**<br>(Study author reported findings)	**Third order constructs**<br>(Meta-ethnography interpretation)
**Article 1.**<br>Corbett, M., Foster, N. and Ong, B. (2007)	It [the back pain] is there all the time and it's horrible for the children to see you in pain and just …it's just …I can't adopt to a different way of lifestyle other than what I've been—how I've been in the past and it's …I just can't fit into …to how the …well, to manage the pain and …an carry on like that. It's just, I just want to know what …what the pain is.<br><br>…but I would still like to know for sure that :here's no … no other damage that's happened that could be put right. You know what I mean? 'Cos you hear of people, you know, certain discs have collapsed or whatever, and they can remove it, you know, and they're o.k. You know, why can't that happen to me? Why can't they do something and I'm o.k.?	**Insufficient medical and scientific explanation.**<br>- People with CLBP want/need to know the cause of their pain, before they are willing make next steps towards adapting their lifestyle.<br>- However, identifying the specific underlying pathology of CLBP is notoriously difficult and often there is not a structural cause.<br>- They are often labelled 'non-specific low back pain' which makes acceptance difficult.<br>- They feel like they are not believed. Symptoms not legitimised.	**Diagnostic uncertainty**<br>- People with CLBP want a clear diagnosis or acceptable

			explanation for their symptoms.<br>- +/- diagnostic investigations
	I wouldn't benefit from having an X-ray done because she knows what is happening to me. Er, she can tell from examining me, you know, about the spine and everything. So she didn't really feel that an X-ray … We wouldn't achieve anything, you know, because I don't think there's anything that you can physically do to put things right.<br><br>…which makes me think as there's something wrong and hopefully things can be made better.	**Expected linear progression:**<br>- People with CLBP believe a clear diagnosis leads to effective treatment and possible cure. When this doesn't happen, it increases uncertainty and reduces hope.<br>- Even more pronounced when HCPs suggest key diagnostic tests will not help.<br>- Without a clear diagnosis, they perceive their prognosis and the chance of a cure is limited.	
	That particular period in my life took me down to a place that I don't ever, ever want to go back to. It's purely through pain, and I think it's only somebody who has suffered from constant … constant pain can really understand that. I really did feel like I was going insane and … and there were, well, there was more than a couple of instances, then, when I would get up at three o'clock in the morning, wanting more pain killers and I … I sat at the table, looked at the bottle and thought "Wah, I just want a rest from this",	**Psychological components/despair resulting from living with chronic pain**<br>- Psychological despair caused by persistent pain<br>- Nearly all respondents considered the psychological aspects of CLBP as inextricably linked to physical aspects.<br>- Managing pain is essential for psychological well-being.	**Prognostic uncertainty**<br>- Psychological component/well-being.
		- The **fluctuating symptoms / unpredictable nature of CLBP** makes it difficult to manage causing a cyclic process of hope and despair, mixed with uncertainty.<br>- People with CLBP believe nothing can be done, but do not want to live with hopelessness, thus raises doubt.	**Prognostic uncertainty**<br>- Unpredictable symptoms

	… at the moment, like I say, I'm having every Wednesday off as annual leave until Christmas, but eventually, I've got no annual leave left, I've got to do full-time. You know, I can't have one day a week off. I've got to do full-time and I'm finding it very hard and I'm frightened that I'm going to do it because I have to, but then I end up getting worse and I just can't cope. What do I do? Because that worries me. I can't go off sick. I can't afford to go on half pay. So .. so that's a real dilemma and then I think: God, I have to work until I'm 65! I've got a mortgage to pay. How … how am I going to cope?, and I know, I shouldn't look so far ahead, but when you … when you've been in pain for several months, you do. You start thinking: what if it never goes right? What if it gets worse? What am I going to do?	- Doubt can lead to hopelessness, but it can also redirect the patient towards hope.	
	I sort of think: well, if I, you know, I'm 31 now and it's bad. How on earth …it's not going to improve, it's not going to fix itself, is it, magically? 'Cos things don't, so surely it can only get worse. (Fiona)<br><br>'cos I have a letter from […] the Specialist, and I hadn't seen it until the Solicitors (had) got all the records and then I read the letter and it said, erm, something about.., basically, "it's here forever so learn to live with it," erm and.. "I can't do anything like surgery or anything at the moment but if you suddenly can't feel your legs and you can't walk, give me a ring and I'll operate.	**Worry and fear for the future**<br>- This pervades nearly all interviews.<br>- Linked with unknown cause/unclear diagnosis and unpredictable/fluctuating symptoms, it creates uncertainty of what their future will look like.	**Prognostic uncertainty**<br>- Fear of the unknown<br>- Impact on their self-identity / future
	They [others with back pain] go through what I've been through. They've got to come through it all: the stress, the anger, erm…the feeling of ..er… uselessness, and it can take a toll on a marriage and a family so bad, to the point that, that person may not have a family in 18 months, four years…<br><br>It's nice to know that, you know, after all this time, there are little threads of 'light at the end of the tunnel', you know. It's not … it's not all about being unemployed. This is what they've got to get away from. This, not look at it as "that's it, my life's over". They've got to look, now, towards things they can do—positive things they can do.	**Worries for the future is also affected by social and cultural influences:**<br>- Social networks, culture and relationships shapes beliefs and expectations.<br>- Fear of losing relationships (family, marriage) and employment.	**Prognostic uncertainty**<br>- Fear of the unknown<br>- Impact on their self-identity / future

	…which is one of the reasons why I've started back to work, as opposed to sitting here being in pain. I may as well be at work in pain and getting paid for it.		
**Article 2.**<br>Toye, &<br>Barker, (2012)	If you bent in a certain way, and your disc slipped and you are incapacitated, then you are frightened to do that again . . . because that is something that you associate with a bad back. (interview 1)<br><br>I used to be just so frightened, and I'd think . . . the more I aggravated it, the worse it was gonna be, so I would avoid doing things which is completely the wrong thing to do because you just seize up. (interview 2)<br><br>Am I going to get to a point where ten years down the line I have had to stop doing everything that I like . . . I don't know. I suppose that scares me now, and it didn't before because I hadn't really thought about it. (interview 3)	**Finding hope for the future was central to good outcomes.**<br><br>Individuals with CLBP restored hope by making the following three changes:<br><br>1) **Deconstructing fears**<br>e.g. fear of movement, a certain activity and/or needing to protect themselves from further damage.<br><br>Those worse at one year described a fear of loss	**Prognostic uncertainty**<br>- Fear: Impact on self-identity
	I feel that, all these rigid things that you try and put in place to protect yourself, quite often are actually a problem that you have in your mind rather than your back. So I think your mind and your back are quite closely linked. (interview 3)<br><br>I think it is all about relaxation to be honest. To a degree it might be mind over matter, it might be sort of a lot in my head . . . you get all tense and worried that things are going wrong, and that sort of brings on the spasm. (interview 2)	2) **Constructing an acceptable explanatory model**<br><br>- Patients who restored hope accepted the biopsychosocial model and embraced a psychological explanation, but a physical explanation was still important.	**Diagnostic uncertainty**<br>- Biomedical focus vs accepting a biopsychosocial explanation

I feel that, all these rigid things that you try and put in place to protect yourself, quite often are actually a problem that you have in your mind rather than your back. So I think your mind and your back are quite closely linked. (interview 3)

I mean I was doing exercises like . . . stretching my fingers, and I

		- Viewing the body as 'out of balance', not broken helped to resolve the contradiction between needing a medical explanation / clear diagnosis.<br><br>- **Those who were worse at one year adhered to a medical explanation** (biomedical model) and were unable to make acceptable changes to their lifestyle.	
	I am not running round like a headless chicken trying to do everything at once, just taking my time and thinking about myself, and what I am doing . . . just being much more careful . . . whether I am gonna be disciplined and carry on with that . . . I mean, if the sun comes out and I want to do some gardening . . . as long as I am sensible, I think I will be alright. (interview 2)<br><br>And everyday I will just build it up. Yeah, it is just gradual. And I will just carry on with the pacing. Like today I did five minutes. Tomorrow I will do five minutes, ten seconds. And at that, it was very difficult to cut off, when you are feeling really well, to stop, but I will. (interview 2)<br><br>I suppose the worry is not being independent, and that is what I am all about. I pride myself on being independent and I enjoy doing things for myself and other people. (interview 3)	3) **Reconstructing self-identify by making acceptable changes to activity levels**<br><br>- **Those worse at one year felt loss of self-identity and couldn't limit activities or make acceptable changes.**	**Prognostic uncertainty**<br>- Impact on self-identity

These findings support Mishel's theory on uncertainty in healthcare which states that regardless of the underlying health condition, uncertainty prevails when people cannot cognitively appraise information on the state of illness, especially if the

course of the disease is unpredictable, but also if there is a lack of information about the diagnosis and/or prognosis (Mishel, 1988). Moreover, Soundy et al (2014) who developed a framework for hope also recognise that hope is particularly challenged at time of onset, during change or uncertainty. Therefore, this became a critical turning point in the analytical process and the concept of uncertainty became a new line of enquiry.

STEP 3: Second systematic search

Eligibility criteria refined:

- Population: adults (18-70yrs) with chronic low back pain (>3months)
- Articles that discussed hope ***or*** uncertainty from the perspective of the individual with CLBP in the results section of the abstract.

STEP 3 (continued): Second systematic search

Figure 21. A table outlining the databases and search terms for the second systematic search.

**Database**	**Search Terms**
MEDLINE	'Uncertainty OR Uncertain OR Intolerance of uncertainty OR Possibility' AND 'Chronic low back pain' OR 'non-specific low back pain' OR 'persistent low back pain'
CINAHL	'Uncertainty OR Uncertain OR Intolerance of uncertainty OR Possibility' AND 'Chronic low back pain' OR 'non-specific low back pain' OR 'persistent low back pain'

PubMed	‘Uncertainty[Title/Abstract] OR Uncertain[Title/Abstract] OR Intolerance of uncertainty[Title/Abstract] OR Possibility[Title/Abstract] AND ‘Chronic low back pain’[Title/Abstract] OR ‘non-specific low back pain’[Title/Abstract] OR ‘persistent low back pain’[Title/Abstract]
AMED	‘Uncertainty OR Uncertain OR Intolerance of uncertainty OR Possibility’ AND ‘Chronic low back pain’ OR ‘non-specific low back pain’ OR ‘persistent low back pain’
PEDro	‘persistent low back pain’ uncertain*<br>‘chronic low back pain’ uncertain*<br>‘non-specific low back pain’ uncertain*
SPORTDiscus	‘Uncertainty OR Uncertain OR Intolerance of uncertainty OR Possibility’ AND ‘Chronic low back pain’ OR ‘non-specific low back pain’ OR ‘persistent low back pain’
Google Scholar	allintitle: uncertainty "low back pain". 2003-2024.
ScienceDirect	‘Uncertainty’ AND ‘low back pain’ - in title and abstract.

STEP 3 (continued): Search Process

Figure 22. NHS Knowledge and library hub search strategy screenshot.

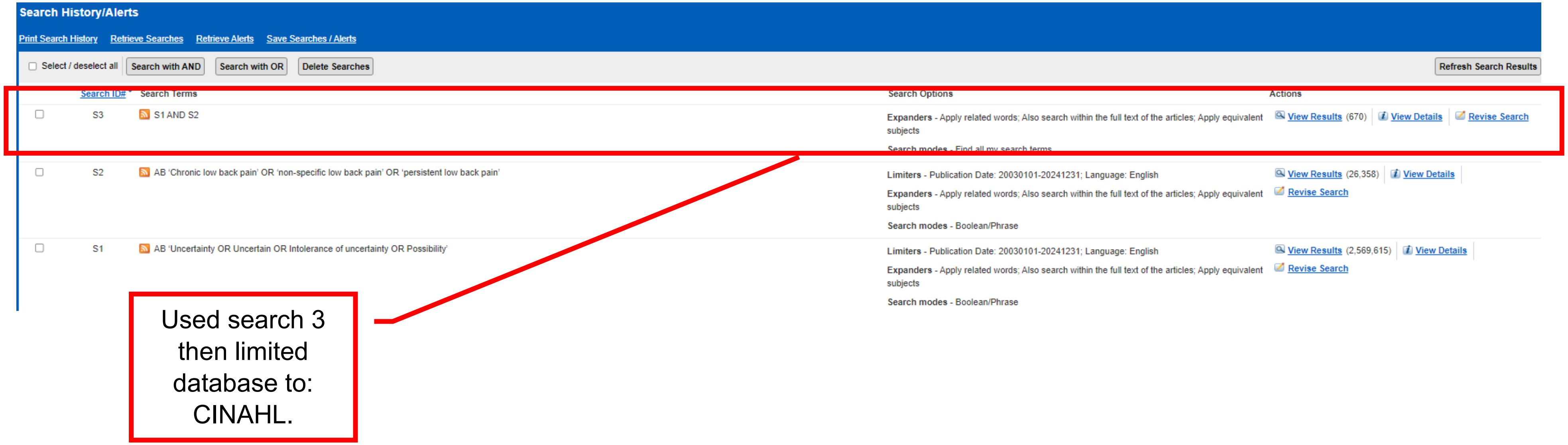

<u>STEP 3 (continued):</u> Search Process

Figure 23. A table outlining the search results for CINAHL.

**Database**	No. of articles	Met eligibility criteria	Articles:
CINAHL	99	0	**Excluded:**<br><br>• Bourke, M., Ferguson, D. & Cooke, M. (2022) 'Patient experiences of self-management for chronic low back pain: a qualitative study', *Physical Therapy and Rehabilitation Journal*, 102 pp. 1-10. doi.org/10.1093/ptj/pzac030<br>**Reason not included:** It didn't discuss or measure hope or uncertainty, rather the key theme of the model was 'fluctuating uncertainty' which was developed from 6 sub themes

STEP 3 (continued): Search Process

Figure 24. A screenshot of the search process for MEDLINE.

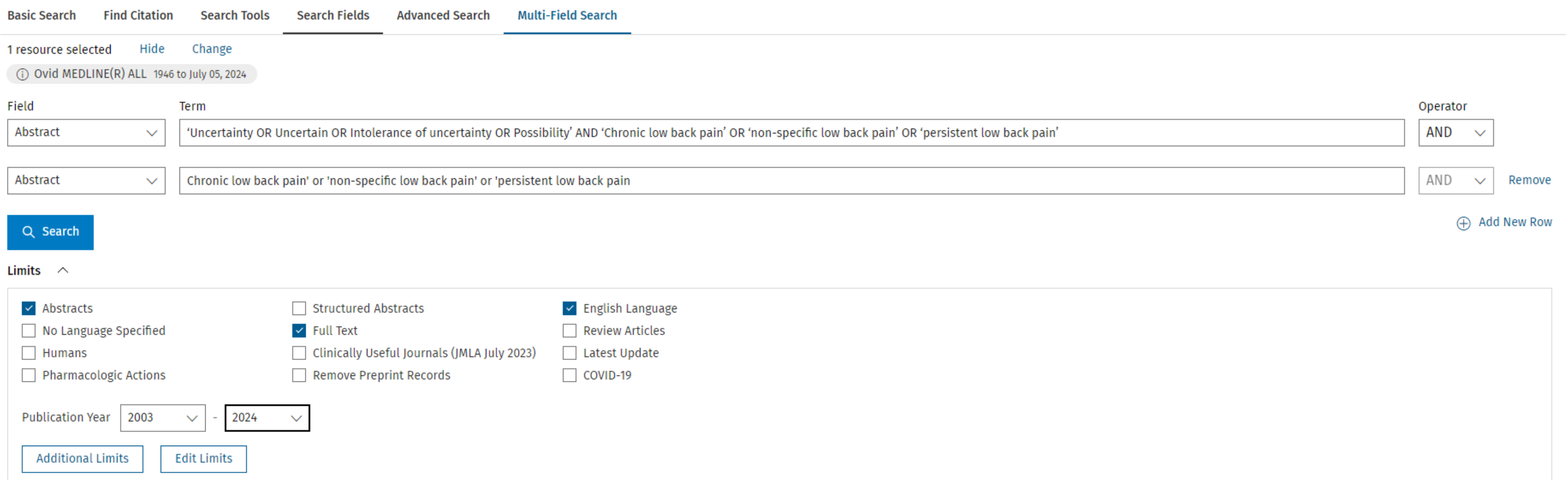

STEP 3 (continued): Search Process

Figure 25. A table outlining the search results for MEDLINE.

**Database**	No. of articles	Met eligibility criteria	Articles:
MEDLINE	234	1	**Accepted:**<br>• Choi, J.W., So, W.Y., and Kim, K., (2022) 'The Mediating Effects of Social Support on the Relationship between Uncertainty and Quality of Life among

			Patients with Chronic Low Back Pain: A Cross-Sectional Survey', *Healthcare*, 10 (9) e1805. doi.org/10.3390/healthcare10091805.<br><br>**Excluded:**<br><br>• Slade, S., Molloy, E. and Keating, J. (2012) 'The dilemma of diagnostic uncertainty when treating people with chronic LBP: a qualitative study', *Clinical rehabilitation,* 26 (6) pp. 558-569<br>**Reason not included:** Physiotherapists perceptions, not patients.<br><br>• Bourke, M., Ferguson, D. & Cooke, M. (2022) 'Patient experiences of self-management for chronic low back pain: a qualitative study', *Physical Therapy and Rehabilitation Journal*, 102 pp. 1-10. doi.org/10.1093/ptj/pzac030<br>**Reason not included:** It didn't discuss or measure hope or uncertainty, rather the key theme of the model was 'fluctuating uncertainty' which was developed from 6 sub themes.

STEP 3 (continued): Search Process

Figure 26. A screenshot of the AMED (available through OVID platform) search strategy:

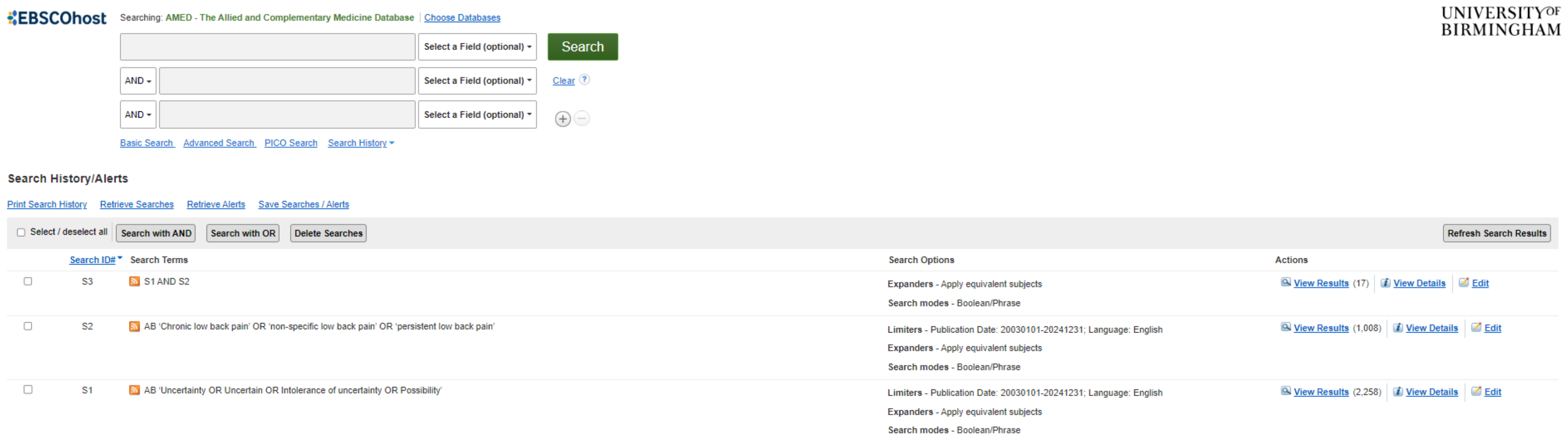

Figure 27. A table outlining the search results for AMED:

**Database**	No. of articles	Met eligibility criteria	Articles:
AMED	17	0	

STEP 3 (continued): Search Process

Figure 28. Screenshots (x3) of the PEDro search strategies.

Search 1:

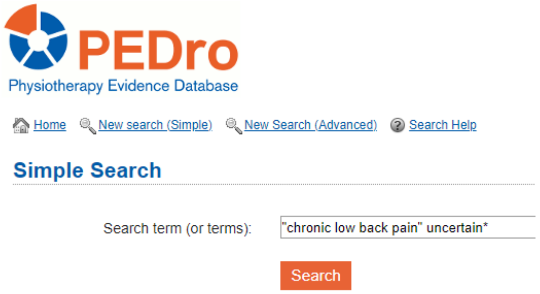

Search 2:

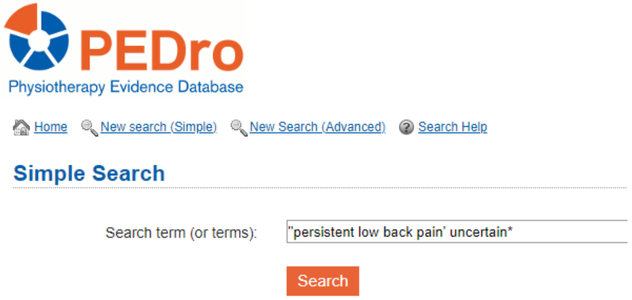

Search 3:

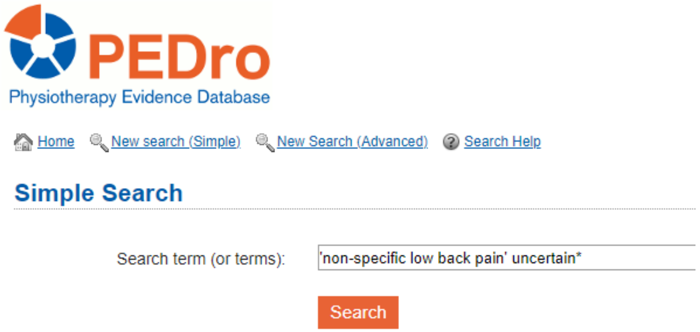

Figure 29. A table outlining the search results for PEDro

**Database**	No. of articles	Met eligibility criteria	Articles:
PEDro (Search 1)	30	0	
PEDro (Search 2)	3	0	
PEDro (Search 3)	14	0	

<u>STEP 3 (continued):</u> Search Process

Figure 30. A screenshot of the PudMed search strategy.

History and Search Details — Download — Delete

Search	Actions	Details	Query	Results	Time
#1	•••	>	Search: **('Uncertainty[Title/Abstract] OR Uncertain[Title/Abstract] OR Intolerance of uncertainty[Title/Abstract] OR Possibility'[Title/Abstract]) AND (Chronic low back pain'[Title/Abstract] OR 'non-specific low back pain'[Title/Abstract] OR 'persistent low back pain'[Title/Abstract])** Filters: **Abstract, Full text, English, from 2003 - 2024**	224	09:29:54

Figure 31. A table outlining the search results for PubMed

**Database**	No. of articles	Met eligibility criteria	Articles:
PubMed	224	1	**Accepted:**<br>• Choi, J.W., So, W.Y., and Kim, K., (2022) 'The Mediating Effects of Social Support on the Relationship between Uncertainty and Quality of Life among Patients with Chronic Low Back Pain: A Cross-Sectional Survey', *Healthcare*, 10 (9) e1805. doi.org/10.3390/healthcare10091805.<br>**Excluded:**<br>• Slade, S., Molloy, E. and Keating, J. (2012) 'The dilemma of diagnostic uncertainty when treating people with chronic LBP: a qualitative study', *Clinical rehabilitation*, 26 (6) pp. 558-569. **Reason not included:** Physiotherapist's perceptions, not patients.<br>• Serbic, D and Pincus, T. (2015) 'Diagnostic uncertainty and recall bias in chronic low back pain', *Bone & Joint*, 155 (8) pp. 1540-1546. **Reason not included:** No outcome measure of hope or discussion on hope or uncertainty from the participants perceptive.

<u>STEP 3 (continued):</u> Search Process

Figure 32. A screenshot of the SPORTDiscus search strategy.

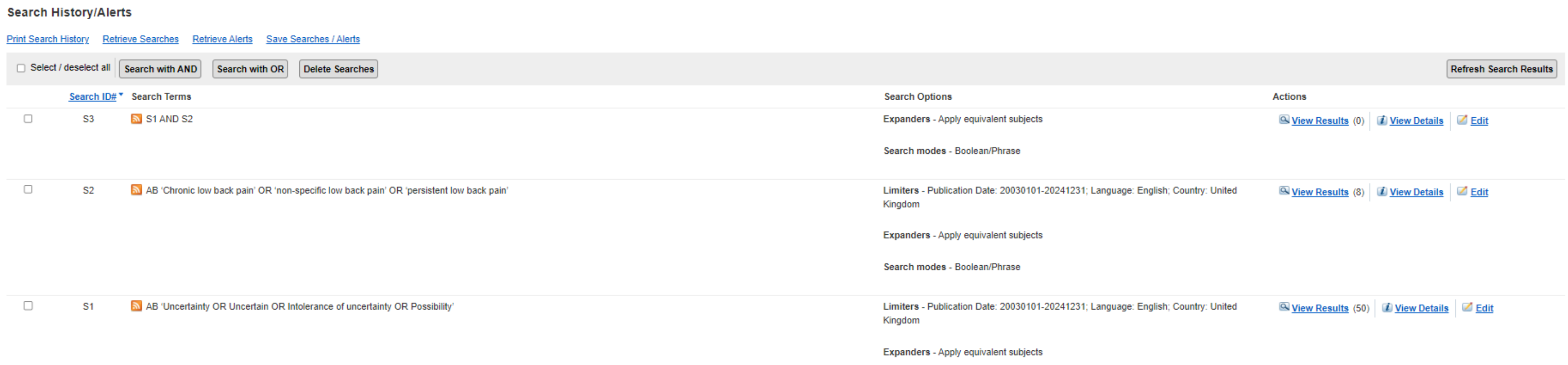

Figure 33. A table outlining the search results for SPORTDiscus.

**Database**	No. of articles	Met eligibility criteria	Articles:
SPORTDiscus	0	0	

STEP 3 (continued): Search Process

Figure 34. A screenshot of the Google Scholar search strategy
(NB: Unable to limit language)

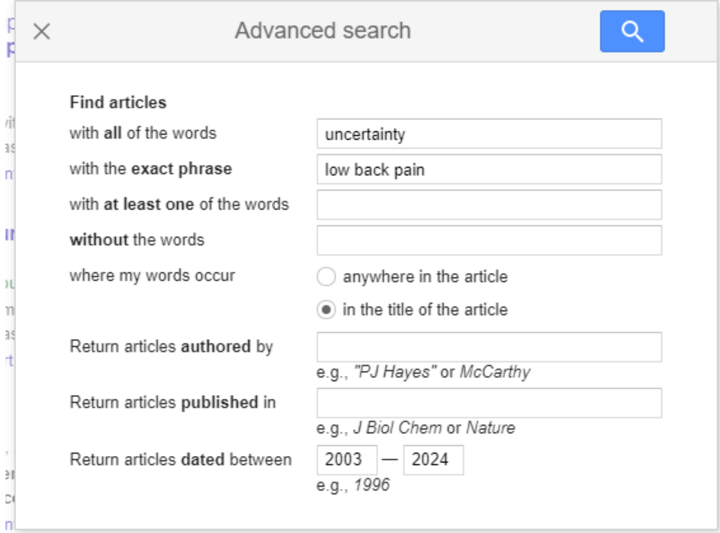

Figure 35. A table outlining the results for Google Scholar.

**Electronic Database**	No. of articles	Met eligibility criteria	Articles:
Google Scholar	12	3	**Accepted:**<br>• Choi, J.W., So, W.Y., and Kim, K., (2022) 'The Mediating Effects of Social Support on the Relationship between Uncertainty and Quality of Life among Patients with Chronic Low Back Pain: A Cross-Sectional Survey', *Healthcare*, 10 (9) e1805. doi.org/10.3390/healthcare10091805.<br>• Costa. N, Butler, P., Dillon, M., Mescouto, K., Olson, R., Forbes, R. and Setchell, J. (2022a) "I felt uncertain about my whole future" – a qualitative investigation of people's experiences of navigating uncertainty when seeking care for their low back pain', *PAIN*, 164 (12) pp. 2749-2758.<br>• Costa, N., Olson, R., Mescouto, K., Hodges, P.W., Dillon, M., Evans, K., Walsh, K., Jensen, N. and Setchell, J. (2023) 'Uncertainty in low back pain care – insights from an ethnographic study', *Disability and Rehabilitation*, 45 (5) pp. 784-795.

			**Excluded:**<br><br>• Slade, S., Molloy, E. and Keating, J. (2012) 'The dilemma of diagnostic uncertainty when treating people with chronic LBP: a qualitative study', *Clinical rehabilitation*, 26 (6) pp. 558-569.<br>**Reason not included:** Physiotherapist's perception, not patients.<br><br>• Costa. N, Mescouto, K., Dillon, M., Olson, R., Butler, P., Forbes, R. and Setchell, J. (2022b) 'The ubiquity of uncertainty in low back pain care', *Social science and medicine*, 313, e115422.<br>**Reason not included:** Physiotherapists perception, not patients.

<u>STEP 3 (continued):</u> Search Process

Figure 36. A screenshot of the ScienceDirect search strategy.

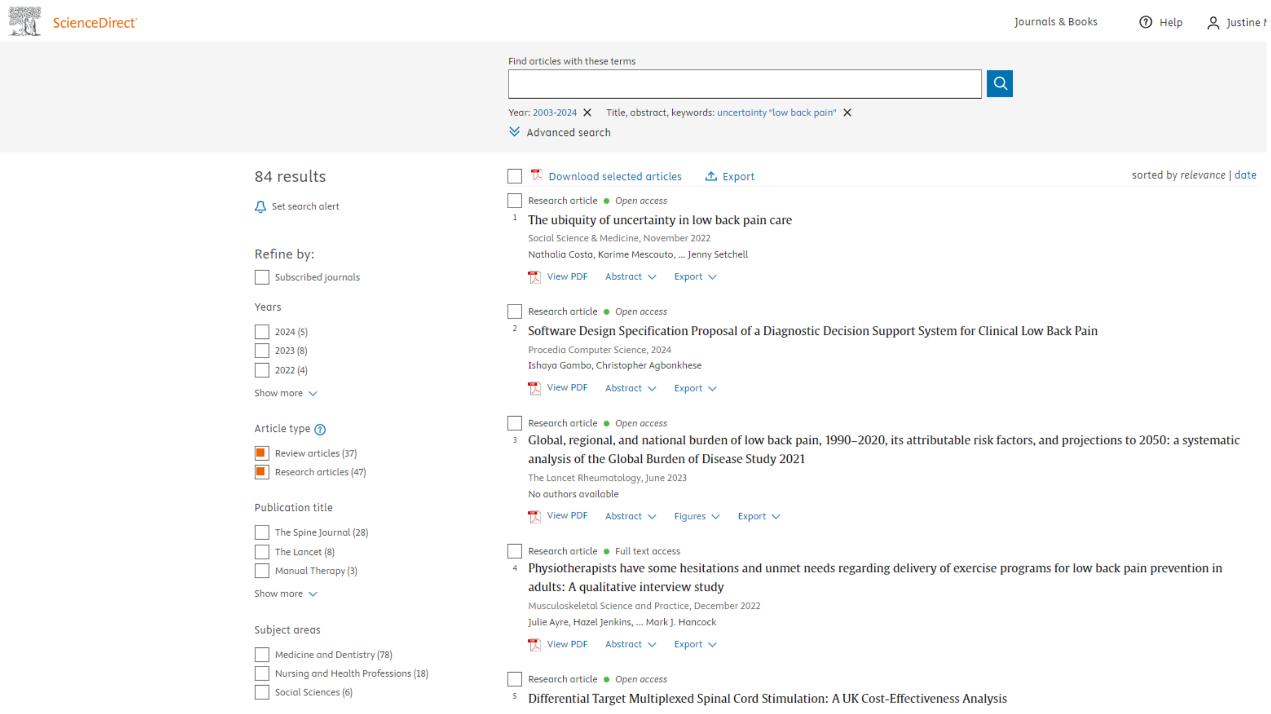

STEP 3 (continued):

Figure 37. A table outlining the results for ScienceDirect.

**Electronic Database**	No. of articles	Met eligibility criteria	Articles:
ScienceDirect	84	0	**Excluded:**<br>• Koleck et al (2006) 'Psycho-social factors and coping strategies as predictors of chronic evolution and quality of life in patients with low back pain: A prospective study', *European journal of pain*,<br>**Reason not included:** Predictors of developing chronic pain – not patients with chronic pain.<br><br>• Serbic, D and Pincus, T. (2015) 'Diagnostic uncertainty and recall bias in chronic low back pain', *Bone & Joint*, 155 (8) pp. 1540-1546.<br>= Higher levels of depression and disability were found in the group with diagnostic uncertainty, but levels of pain intensity did not differ between the groups.<br>**Reason not included:** No outcome measure of hope or discussion on hope or uncertainty from the participants perceptive.<br><br>• Costa. N, Mescouto, K., Dillon, M., Olson, R., Butler, P., Forbes, R. and Setchell, J. (2022b) 'The ubiquity of uncertainty in low back pain care', *Social science and medicine*, 313, e115422.<br>**Reason not included:** Physiotherapist's perception, not the patients.

STEP 3 (continued):

Figure 38. A table summarising the articles which met the eligibility criteria following the second systematic search.

**Database**	**Number of articles**
MEDLINE	234
CINAHL	99

AMED	17
PEDro	47
PubMed	224
SPORTDiscus	0
Google Scholar	12
ScienceDirect	84
**Total**	**717**
**Removed duplicates**	**51**
**Records screened**	**666**<br>(excluded 658 by title/abstract)
**Records sought for retrieval**	**8**
**Reports excluded**	**5**
**Included**	**3**<br>(2 x qualitative and 1 x quantitative articles)

Qualitative articles (x2)

- Costa et al (2022a)
- Costa et al (2023)

Quantitative articles (x1)

- Choi, J.W., So, W.Y., and Kim, K. (2022)

<u>REPEAT STEP 2:</u> Further immersive reading, coding and exploring how studies relate.

Figure 39. A table outlining the coding of the two qualitative articles on uncertainty in people with CLBP.

	**First order coding**<br>(Participant verbatim quotes):	**Second order coding**<br>(Study author reported findings)	**Third order constructs**<br>(Meta-ethnography interpretation)
**Costa et al (2022a)**<br><br>**(Inclusion criteria:** All participants had experienced LBP for at least 2 years)	*I remember one in particular, who was a really nice doctor, but I went to see her and she said, "you got arthritis, it will get worse." That was really awful, because she didn't really give me any guidance as to what I could do… So I felt very uncertain then about my whole future…* Jessica, aged 75 years, 10 years since the first LBP episode<br><br>*Frustrated again… I wasn't mentally or physically prepared to, or even emotionally prepared to start having a chronic condition. I was still hoping that it would be an acute condition. But as time passed, I sort of saw it progressing, the pain was still the same sometimes going up, going down, but not going away… the uncertainty of not knowing how this condition will resolve… And I'd say that, although I've been with pain for the last two years, I'm still poorly prepared to manage uncertainty.* Jorge, aged 36 years, 2 years since the first LBP episode<br><br>*The first confusion for me or uncertainty is the fact that I've had the same thing, the same type of pain come and go many, many times for many years, and then I was certain that something serious was going to come up on that test, but it didn't. So, it's like, okay, so I don't have anything, which is great. I don't have anything serious but this pain… this pain just can't be explained in a test. So that leaves me with this question in my head, because I know that it could come back at any time… and I still won't know, the actual reason why it happens, because the test came back with no issues at all…* Luke, aged 32 years, 12 years since the first LBP episode	**Uncertainty was situated in….**<br><br>**Time (past, present and future).**<br>- Certainty of having a specific diagnosis and the certainty of not having one both produced uncertainty about their future.<br>e.g. when MRI results don't correlate with their symptoms or if incidental findings arose, it caused further uncertainty regarding the cause, future episodes and management.	**Diagnostic uncertainty**<br>- Patients want a clear diagnosis or acceptable explanation for their symptoms.<br>- +/- diagnostic investigations
	I've had a few doctors in the past for previous things that maybe they didn't show enough care, or I didn't get the right answers that I wanted. Maybe that had some influence as to why I didn't go back… I just felt like I was rushed out the door, to get to the next patient. And it happened quite a few times… then there is the cost of all the X-rays and whatnot. And then with the chiropractic sessions, you have to come back from multiple sessions and then it still might not have any extra outcome or any extra benefits. You know, I've spent $600 and it hasn't gotten me any better. Jason, aged 29 years, 10 years since the first LBP episode	**Clinicians – can they help me?**<br><br>- Ability and willingness to help	**The clinical encounter**

	The key thing for me would be, as part of any training package, is making sure you actually listen to the person and don't assume! Shuffle your assumptions out the window or up your ass… Don't assume because that's, that's probably the most frustrating part for almost all of us. Is the assumption that "it's not as bad" or "we're lazy." Or we may, you know, we're trying to milk the system to get drugs or whatever woeful ideas some idiots get… Even actually utilising uncertainty. Approaching it with that uncertainty of what, what exactly is going on here, for that person? Instead of "okay, you've got back pain. This is what we do." Anna, aged 53 years, 30 years since the first LBP episode	- Level of expertise<br>- Listened to and understood	
	The line of questioning, I still remember just trying to figure out how is this all adding up to painting the wrong… How does it all hang together? Is this just another symptom of what's going on over there? Is it something different?… I was a bit confused because I just… where was this going? Did I have cancer in my back or what? What was going on? So yeah, just lead you to be a bit more concerned about what's going on… Helen, aged 55 years, 30 years since the first LBP episode<br><br>*Being a bit more honest, and upfront and consistent, saying at the start "this isn't a straightforward process, we're just going to be honest with you." Because I guess clinically, there is a lot of uncertainty around low back pain anyway, so it is very hard for them to diagnose but if they're honest with that, I think for a lot of reasonable people, and maybe it's different when someone's in, like a whole world of pain. But for me, because it was not like terrible pain, that like if someone's just upfront and they're, "look like this is how it is with back pain, what we know we can do is this, and we're just going to see, and then we'll check in"… almost pre-empting discussions, starting off by explaining that and yeah, just a bit more consistency, seeing that there is like a plan in place and like knowing that.* Lara, aged 21 years, 3 years since the first LBP episode	**What clinicians are asking?**<br>- Uncertainty around what/why clinicians are asking specific questions.<br>- Exploring concerns<br>- Honesty about uncertainty)	**The clinical encounter**<br>- Identifying psychosocial factors<br>- Epistemic humility
	He [the doctor] sort of made some comment like "you are trying to affect, no good deed goes unpunished." And I'm just looking at him going "I'm in tears because I am in pain!"… He [the doctor] was horrid. Everybody does take me incredibly seriously, except this last time. Simone, aged 61 years, 2.5 years since the first LBP episode	**Am I being taken seriously? (Legitimacy of symptoms)**<br>Uncertainty and negative emotions are produced when patients feel that they are heard, understood or believed.	**The clinical encounter**<br>- Epistemic humility

	Sometimes I felt uncertain whether I'm actually being taken seriously… because sometimes I feel like unless your pain is visible, people sometimes have a hard time relating to it or understanding it or believing it. So, I feel sometimes like I have to work extra hard for people to take me seriously. So often I'll leave appointments and then the more I think about them later: Did I tell them enough today? Did they ask the right questions? Did I convince them enough that they're going to help me?… [it] can actually have a very negative effect afterwards because then you start going over it in your mind and questioning yourself, and then you just sort of leave a medical appointment almost feeling worse than when I went in. Emma, aged 34 years, 12 years since the first LBP episode<br><br>*I knew that I wasn't imagining the stuff that was going on or the amount of pain I was in with my back… Ask me more questions if you need to know about my symptoms. I don't care if you ask me them six times back to front… do whatever you need to do to understand that this is real for me. Listen to your patient. That's probably the biggest thing—put bias aside. I feel like there was an assumption there made on me, which I'm not taking personally, I just think it's problematic. And it stops access [to healthcare]. It makes access [to healthcare] terrible and it makes the experience hard.* Carla, aged 44 years, 15 years since the first LBP episode		
**Costa et al (2023)**	Grace was sitting on a chair and JadePhysio sat on a stool facing her […] "If I tell you that it hurts here, do you know which nerves come in here?" Grace asked in a very natural way, and JadePhysio said that she could tell her, but it was more likely that her pain was probably coming from her muscles rather than her nerves. JadePhysio asked Grace to stand up and do some movements with her back. (Grace – 18 months, 8/10; JadePhysio – 11-15 years)<br><br>JohnPainMgmt comes back and sits down, facing Leah. He adopted a quite 'open' posture – sitting tall, feet on the floor, hands resting on his thighs, looking straight to her […] "Interestingly enough, ratty looking backs [in scans] sometimes do not cause problems … and there are some pristine looking backs that can cause a lot of pain so that leans a bit off the weight towards that potential facets causing pain … you also have SIJ pain and it can be hard to differentiate". (Leah - 6 years, 8/10; JohnPainMgmt – over 15 years)<br><br>"Do you know that my MRI demonstrated that I have damage on the left, but I'm feeling pain on the right. What is the explanation for that?" Anika asked. MiaPhysio hesitated a little on the answer, but she tried to explain that it was because of overall sensitisation and that she already saw a lot of patients with the same presentation and that they were fine. Anika didn't say anything. (Anika – 8 years, 3/10; MiaPhysio – 5–10 years)	**Sources of uncertainty**<br>- **Patient**<br>Expects definitive answers as they seek information about the cause (including structural causes), prognosis and imaging findings (but often a mismatch between MRI results and clinical presentation).<br><br>- **Clinician – Often neglect uncertainty during clinical interactions due to the complexity of the condition.**<br>There is a need for clinicians to disclose uncertainty.	**Diagnostic and prognostic uncertainty**<br><br>**The clinical encounter**<br>- Epistemic humility vs epistemic injustice

	CarolPhysio asked again if Sara had any idea of what could have been the cause of her pain and what could be contributing to her back. "I'm never able to see what triggers it" Sara mentioned in almost a tired/bit angry voice and added "Would be nice to know what triggers it" and almost sounded hopeful. CarolPhysio was doing some rocking movement on Sara's back, in a very soothing rhythm. "What are you planning to do in Africa?" CarolPhysio asked. (Sara – 16 years, 1/10; CarolPhysio – over 15 years)	Uncertainty was also produced during interactions with the clinician – e.g. questions around aggravating activities, patients find it challenging to answer, as activities that cause pain are not consistent. These conversations were filled with emotions (frustration, anger etc).	
	Different from the other sessions, I've noticed that OscarPainSpDr positioned his computer between him and Mila, almost like he wanted to have something to divide them (since the desk was pressed against the wall). He spoke in a very low and smooth tone of voice that contrasted with his reserved look […] OscarPainSp mentioned that healthcare professionals now have a different approach to pain, since the body and mind are more recognised as connected. He mentioned that the 'best evidence' showed that this connection between mind and body would better be addressed through things such as psychology or physiotherapy, rather than medication. He talked for a while about this evidence, and how structure was not related to pain. (Mila – 3 years, 7/10; OscarPainSpDr – 11–15 years)<br><br>Chelsea: I've suffered enough. I ran out of options. [Chelsea starts crying].<br><br>TimPhysio: You haven't run out of options, you can try prolotherapy but they don't want to do that until you try other things.<br><br>Chelsea: Today I was driving here and I noticed that I could push the accelerator without having too much pain and I celebrated it. I've learnt how to change my mind – I used to be a high achiever but now I celebrate all these little things… [Chelsea keeps crying. TimPhysio grabs the box of tissues and gives it to Chelsea. She thanks him and starts wiping her eyes.]<br><br>"I'm just tired. I have tried everything. You know, I work for myself because of this crap. […] I don't know what is going on and I have no idea how I can fix it." (Karen – 20 years 8/10; OmarPainSpDr – 5-10 years)	**Neglecting complexity**<br>- Clinicians try and decrease uncertainty and its emotional effects by neglecting its complexity and focusing on the psychosocial or pathoanatomical causes, but not both.<br><br>Having tried many treatment interventions which have failed and seen by numerous or various clinicians, patients felt hopeless.	**The clinical encounter**<br>- Epistemic humility vs epistemic injustice

	MiaPhysio came back with a skeleton model of a back and explained to [patient Valeria] what was going on anatomically. "With treatment and exercise you will be back very quickly" MiaPhysio reassured Valeria.<br><br>BradleyGP was looking at Rana while she was talking but then he would quickly check his computer to look at the notes in the system every now and then […]<br><br>Rana: Do you think the injections will help? It didn't really help my shoulder …<br><br>BradleyGP: There is no guarantee. Everything in pain medicine is a trial and error. It should work but it may not work. Let's give it a go and hope for good outcomes. I will see you in two or three months. Do you have any other questions?<br><br>Rana: Not really … thanks, BradleyGP.<br><br>Rana and her husband thanked BradleyGP and left.<br><br>(Rana – 38 years, 7/10; BradleyGP – less than 5 years)	**Attending to uncertainty**<br><br>- Clinicians often reduce it, as opposed to attend to it.<br>- They reduce uncertainty by emphasising a definite likelihood of improvement (common strategy).	**The clinical encounter**<br>- Epistemic humility vs epistemic injustice

Figure 40. Exploring how the core four studies on hope and uncertainty translate.

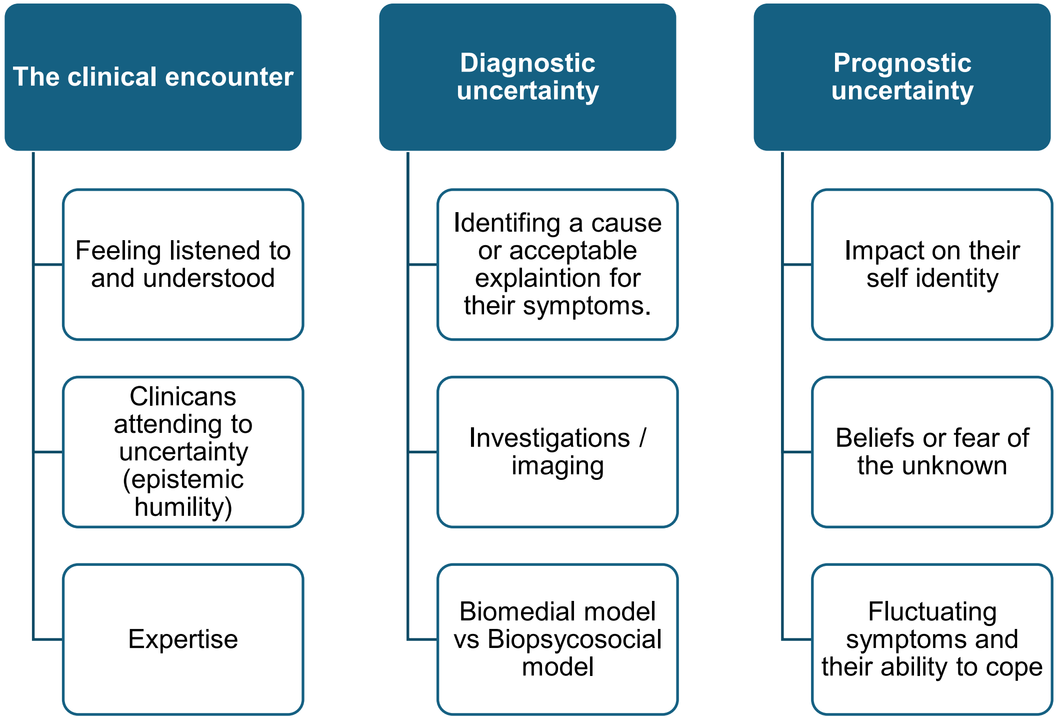

<u>STEP 4:</u> Iterative process of idea generation

Figure 41: A table considering the different concepts to ensure alignment with thoughts and the process of testing information

Identifying and exploring concepts and constructs that help support understanding of the model.

1. Situating the critical ideas of uncertainty as a core concept for the model.

Summary table: Comparison of Uncertainty, and Intolerance of Uncertainty

Concept	Core Idea	Key Strategies	Context Sensitivity	Strengths	Limitations
Uncertainty (Careton, 2016)	A state of having limited knowledge about outcomes, leading to unpredictability and ambiguity.	Information seeking, tolerance building, probabilistic reasoning.	Moderate – varies by domain (e.g., health, decision-making, risk).	Encourages exploration and learning in complex environments.	Can provoke anxiety and hinder decision-making if unmanaged.
Intolerance of Uncertainty (Dugas, & Robichaud, 2007)	A dispositional tendency to perceive uncertain situations as stressful and unacceptable.	Cognitive restructuring, exposure therapy, acceptance-based approaches.	High – influenced by personality, mental health status, and situational factors.	Identifying intolerance can guide targeted interventions for anxiety and OCD.	Associated with maladaptive coping, avoidance, and heightened distress.
Cognitive Flexibility (Martin et al., 1995)	The mental ability to switch between thinking about different concepts and to adapt behaviour to achieve goals in changing environments.	Reframing, perspective-taking, problem-solving, mindfulness practices.	High – involves adapting to situational demands and shifting perspectives.	Supports resilience, creativity, and adaptive functioning.	Can be impaired by stress, anxiety, or rigid thinking patterns.

References:

Martin, M. M., & Rubin, R. B. (1995). A new measure of cognitive flexibility. Psychological Reports, 76(2), 623–626.

Carleton, R. N. (2016). Into the unknown: A review and synthesis of contemporary models involving uncertainty. Journal of Anxiety Disorders, 39, 30–43.

Dugas, M. J., & Robichaud, M. (2007). Cognitive-behavioral treatment for generalized anxiety disorder: From science to practice. Routledge.

Summary Table: Frameworks for Intolerance of Uncertainty (IU) and identifying supportive comments and concepts

Framework	Key Contributors	Core Concepts	Mechanisms	Comments useful to articles
Cognitive-Behavioral Model	Freeston et al. (1994)	IU as a dispositional trait	Threat overestimation, avoidance, negative problem orientation	· IU is defined as a dispositional trait—a stable tendency to react negatively to uncertain situations.<br>· Individuals high in IU experience emotional, cognitive, and behavioural distress when faced with ambiguity or unpredictability<br>· Individuals high in IU overestimate the possibility and severity of negative outcomes – this reinforces worry
Intolerance of Uncertainty Model (IUM)	Dugas et al. (1998)	IU central to worry	IU, positive beliefs about worry, poor problem orientation, cognitive avoidance	· Defined as a dispositional tendency to react negatively to uncertain situations.<br>· IU drives worry as a coping mechanism to reduce uncertainty.<br>· Individuals interpret ambiguous situations as potentially harmful<br>· Complications: (1) worry seen as positive as it is identified as a way to prepare for negative outcome or solving problems, seeming protective overtime (2) problems are seen as threats and

				this can lead to avoidance of problem solving which maintains worry. (3) distressing thoughts are avoided by engaging verbally about worry which in turn sustains anxiety
Uncertainty and Threat Model	Carleton (2016)	Fear of the unknown	Uncertainty is aversive even without threat	· Fear of the unknown is defined as “an individual’s propensity to experience fear caused by the perceived absence of information at any level of consciousness or point of processing.”<br>· Relation to Intolerance of Uncertainty (IU): IU is described as “a dispositional incapacity to endure the aversive response triggered by the perceived absence of salient, key, or sufficient information.” IU is essentially the behavioral and emotional manifestation of fear of the unknown.
Emotion Regulation & IU	Aldao et al.	IU affects emotion regulation flexibility	Difficulty adapting emotional responses in uncertain contexts	· Individuals with a high intolerance to uncertainty may struggle with emotional regulation flexibility<br>· High intolerance to uncertainty can impair the ability to assess context accurately, select appropriate strategies and adapt emotional responses<br>· High intolerance to uncertainty contributes to maladaptive coping and is associated with psychological disorders.

References

Aldao, A., Sheppes, G., & Gross, J. J. (2015). Emotion regulation flexibility. Cognitive Therapy and Research, 39(3), 263–278. https://doi.org/10.1007/s10608-014-9662-4

Carleton, R. N. (2016). Into the unknown: A review and synthesis of contemporary models involving uncertainty. Journal of Anxiety Disorders, 39, 30–43. https://doi.org/10.1016/j.janxdis.2016.02.007

Dugas, M. J., Gagnon, F., Ladouceur, R., & Freeston, M. H. (1998). Generalized anxiety disorder: A preliminary test of a conceptual model. Behaviour Research and Therapy, 36(2), 215–226. https://doi.org/10.1016/S0005-7967(97)00070-3

Freeston, M. H., Rhéaume, J., Letarte, H., Dugas, M. J., & Ladouceur, R. (1994). Why do people worry? Personality and Individual Differences, 17(6), 791–802. https://doi.org/10.1016/0191-8869(94)90048-5

McEvoy, P. M., & Mahoney, A. E. J. (2012). To be sure, to be sure: Intolerance of uncertainty mediates symptoms of various anxiety disorders and depression. Behavior Therapy, 43(3), 533–545. https://doi.org/10.1016/j.beth.2011.02.007

Identifying the main theories of emotional regulation, hope, predisposition factors, BAS activation, reappraisal that provide information that could help explain the processes and outcomes when dealing with the unknown. Identifying the used theory.

Summary table: Emotional Regulation Main Theories

**Model/Theory**	**Core Idea**	**Key Strategies**	**Context Sensitivity**	**Strengths**	**Limitations**
Process Model (Gross, 1998;2014)	Emotion regulation occurs at different stages of the emotion-generative process.	Situation selection, modification, attentional deployment, cognitive change, response modulation.	Moderate – acknowledges timing but less focus on goals or environment.	Clear structure; widely used in research and therapy.	Can be too linear; less emphasis on flexibility or learning.

Emotion Regulation as Learned Skill (Wright, 2025)	Emotion regulation is shaped by learning mechanisms (e.g., reinforcement, modelling).	Strategy acquisition through experience, feedback, and social learning.	High – emphasizes how context and feedback shape regulation.	Integrates developmental and learning perspectives.	Less prescriptive about which strategies to use.
Multidimensional/Contextual Models (Martínez-Priego et al., 2024)	Strategy effectiveness depends on goals, context, and individual differences.	Flexible use of any strategy depending on situation.	Very high – context, goals, and values are central.	Promotes adaptability and personalization.	Harder to operationalize in research or therapy.
Emotion Regulation & Human Flourishing (Valenzeula et al., 2025)	Emotion regulation is part of ethical and existential development.	Aligning emotions with values, meaning, and purpose.	High – considers long-term goals and moral context.	Integrates philosophy, ethics, and psychology.	Less empirical; more conceptual and philosophical.
Emotional regulation flexibility (Aldao et al., 2025)	Emotion regulation can be used adaptatively to switch between emotional regulation strategies depending on situation and demands and personal goals.	Implicit/automatic regulation, flexible switching.	High – focuses on adaptability and unconscious processes.	Reflects real-world complexity of emotion regulation.	Still developing; measurement challenges
Implicit vs Explicit emotion regulation (Braunstein et al., 2017) two-dimensional model distinguishing regulation goals (implicit vs. explicit) and change processes (automatic vs. controlled), resulting in	Implicit Emotion Regulation Automatic, unconscious regulation of emotions	Habits, automatic responses, conditioned responses	Efficient, requires less cognitive resources	Efficient, requires less cognitive resources	Efficient, requires less cognitive resources

four classes of emotion regulation strategies					
	Explicit Emotion Regulation Conscious, deliberate regulation of emotions	Cognitive reappraisal, suppression, problem-solving	Flexible, can be adapted to different situations	Flexible, can be adapted to different situations	Requires cognitive effort and awareness

References

Aldao, A., Sheppes, G. & Gross, J.J. Emotion Regulation Flexibility. *Cogn Ther Res* 39, 263–278 (2015). https://doi.org/10.1007/s10608-014-9662-4

Laura Martin Braunstein, James J Gross, Kevin N Ochsner, Explicit and implicit emotion regulation: a multi-level framework, *Social Cognitive and Affective Neuroscience*, Volume 12, Issue 10, October 2017, Pages 1545–1557, https://doi.org/10.1093/scan/nsx096

Gross, J. J. (1998). The emerging field of emotion regulation: An integrative review. *Review of General Psychology, 2*(3), 271–299. https://doi.org/10.1037/1089-2680.2.3.271

Gross, J. J. (2014). Emotion regulation: Conceptual and empirical foundations. In J. J. Gross (Ed.), *Handbook of emotion regulation* (2nd ed., pp. 3–20). The Guilford Press.

Wright, R. N., Adcock, R. A., LaBar, K. S. (2025). Learning emotional regulation: An integrative framework. Psychological Review, 132; 173-203.

Valenzuela, P., Navarini, C., Mercado, J. A., Fowers, B., Panno, A. (2025). Emotional regulation and human flourishing: theoretical and empirical perspectives. Frontiers in Psychology, 16. DOI=10.3389/fpsyg.2025.1565130

Comparison of Hope Theories in Health and Illness

Model/Theory	Core Idea	Key Strategies	Context Sensitivity	Strengths	Limitations
Snyder's Hope Theory (Synder et al., 2002)	Hope is a cognitive motivational system involving goals, pathways, and agency.	Goal-setting, pathway generation, agency enhancement.	Moderate – focuses on individual cognition.	Empirically validated; widely used in therapy.	Less emphasis on emotion or social context.
Herth Hope Index (HHI) (Herth, 1992)	Hope is a multidimensional life force involving readiness, connectedness, and future orientation.	Assessment via HHI; fostering spiritual and relational support.	High – includes emotional and spiritual dimensions.	Useful in nursing and palliative care.	Less dynamic; more descriptive than prescriptive.
Scioli's Integrative Hope Theory (Scioli, & Biller, 2009)	Hope is a future-directed network built from biopsychosocial resources.	Building resilience through biological, psychological, and social means.	High – integrates multiple domains.	Holistic and adaptable.	Complex to measure and apply clinically.
Existential/Narrative Approaches (Soundy et al., 2013)	Hope exists on a continuum between despair and delusion; shaped by meaning-making.	Therapeutic dialogue, narrative reconstruction, acceptance.	Very high – deeply contextual and personal.	Encourages depth and authenticity.	Less structured; harder to quantify.
Positive Psychology /	Hope is a psychological strength	Brief interventions, goal-setting,	Moderate – focuses on	Promotes resilience and optimism.	May overlook deeper existential concerns.

Flourishing (Cutcliffe, 2003)	supporting wellbeing and recovery.	strength-based therapy.	individual strengths.		
Soundy's MEAH Framework (Soundy, 2024)	Hope is shaped by emotional responses and adaptation to illness; includes different types of future hope.	Therapeutic conversations, emotional mapping, Hope and Adaptation Scale.	Very high – integrates emotion, adaptation, and context.	Clinically grounded; supports rehabilitation and chronic care.	Newer model; less widely adopted outside rehabilitation.

References

Snyder, C. R. (2002). Hope Theory: Rainbows in the Mind. Psychological Inquiry, 13(4), 249–275.

Herth, K. (1992). Abbreviated instrument to measure hope: Development and psychometric evaluation. Journal of Advanced Nursing, 17(10), 1251–1259.

Scioli, A., & Biller, H. B. (2009). Hope in the Age of Anxiety. Oxford University Press.

Cutcliffe, J. R. (2003). Reconceptualizing hope in the context of chronic illness. Journal of Advanced Nursing, 41(6), 543–551.

Lopez, S. J., & Snyder, C. R. (Eds.). (2009). Oxford Handbook of Positive Psychology. Oxford University Press.

Soundy, A., Smith, B., Dawes, H., Pall, H., Gimbrere, K., & Ramsay, J. (2011). Patient's expression of hope and illness narratives in three neurological conditions: a meta-ethnography. Health Psychology Review, 7(2), 177–201. https://doi.org/10.1080/17437199.2011.568856

Soundy, A. (2024). Harnessing Hope in Managing Chronic Illness. Routledge.

Theories of Hopelessness in Psychology

Theory	Key Concepts	Originators	Clinical Implications
Learned Helplessness Theory	Repeated exposure to uncontrollable stressors leads to passivity, low motivation, and hopelessness. Individuals stop trying even when escape is possible.	Martin Seligman & Steven Maier (1967)	Explains depression and PTSD; interventions focus on restoring perceived control and promoting 'learned hopefulness'.
Reformulated Learned Helplessness Theory	Attributional style matters: internal, stable, and global attributions for negative events increase vulnerability to depression.	Abramson, Seligman & Teasdale (1978)	Cognitive therapy targets maladaptive attribution styles to reduce depressive symptoms.
Hopelessness Theory of Depression	Depression arises when negative inferential styles (stable/global causes, negative consequences, negative self-characteristics) interact with stressful life events. Hopelessness is a sufficient cause of depression.	Abramson, Metalsky & Alloy (1989)	Predicts depression severity and duration; informs CBT approaches focused on modifying inferential styles.
Beck's Cognitive Theory of Hopelessness	Hopelessness includes cognitive, emotional, and motivational components. It is a key predictor of suicidal intent.	Aaron Beck et al. (1974–1975)	Used in suicide risk assessment (e.g., Beck Hopelessness Scale); therapy focuses on challenging negative expectations.<br><br>Beck proposed that hopelessness is a central cognitive feature of depression. It reflects a negative expectation about the future, where individuals believe that:

			· Nothing will improve<br>· They are powerless to change their situation<br>· Negative outcomes are inevitable<br>· This belief system contributes to passivity, despair, and suicidal ideation, making hopelessness a key predictor of depression severity and suicide risk
Developmental Elaboration of Hopelessness Theory	Childhood emotional abuse fosters depressogenic inferential styles, increasing vulnerability to hopelessness and depression.	Rose & Abramson (1992)	Highlights importance of early intervention and trauma-informed care.

References

Seligman, M. E. P., & Maier, S. F. (1967). Failure to escape traumatic shock. Journal of Experimental Psychology, 74(1), 1–9.

Abramson, L. Y., Seligman, M. E. P., & Teasdale, J. D. (1978). Learned helplessness in humans: Critique and reformulation. Journal of Abnormal Psychology, 87(1), 49–74.

Abramson, L. Y., Metalsky, G. I., & Alloy, L. B. (1989). Hopelessness depression: A theory-based subtype of depression. Psychological Review, 96(2), 358–372.

Beck, A. T., Weissman, A., Lester, D., & Trexler, L. (1974). The measurement of pessimism: The hopelessness scale. Journal of Consulting and Clinical Psychology, 42(6), 861–865.

Rose, D. T., & Abramson, L. Y. (1992). Developmental predictors of depressive cognitive style: A longitudinal study. Journal of Abnormal Psychology, 101(4), 554–560.

Figure 42: A table outlining the first and second research question and its line of argument synthesis (steps 1-4)

**Step 1 (the question):**<br><br>- What are the main psychosocial factors that people with CLBP commonly report that they are uncertain about?<br><br>- How can HCPs influence these psychosocial factors (either positively or negatively) affecting the outcomes for people with CLBP?			
**Step 2: Potential explanations**			
**What I searched?**	**Why?**	**Article:**	**Conclusions:**
Uncertainty<br>Chronic low back pain<br>2003-2024<br>Google scholar	What do patients with CLBP feel most commonly uncertain about?	Costa et al (2023)<br><br>Costa et al (2022a)	**Diagnostic uncertainty**<br>- Cause<br>- Imaging/investigations<br>**The clinical encounter**<br>- Ability (expertise) and willingness to help<br>- Listened to and understood<br>- Aspects of the examination<br>- Exploring concerns<br>- Honesty + open discussions about uncertainty
*First systematic search	What do patients with CLBP feel most commonly uncertain about?	Corbett, M., Foster, N. and Ong, B. (2007)	**Diagnostic uncertainty**<br>- Clear diagnosis or acceptable explanation for their symptoms<br>- Imaging/investigations<br><br>**Prognostic uncertainty**<br>- Psychological component/well-being.<br>- Unpredictable symptoms<br>- Fear of the unknown<br>- Impact on their self-identity / future
*First systematic search	What do patients with CLBP feel most	Toye and Barker (2012)	**Diagnostic uncertainty**<br>- Biomedical focus vs accepting a biopsychosocial explanation

	commonly uncertain about?		**Prognostic uncertainty**<br>- Fear: Impact on self-identity<br>- Impact on self-identity
Framework for hope	What do patients with CLBP feel most commonly uncertain about?	Soundy et al (2014)	***Impact on their future and self-identity**<br>Systematic review – 10 studies.<br>Hope is severely challenged at times of disease onset, during change or uncertainty. Individuals were overwhelmed by impact on their future.<br>Goals are key – but they must be meaningful.<br>HCPs need to understand the patients' self-identity.
Low back pain, Epistemic Humility<br>2003-2024<br>Google scholar	How does the therapeutic relationship between the patient and clinician affect uncertainty?	Buchman et al (2017) 'Investigating trust, expertise and epistemic injustice in chronic pain', *Bioethical Inquiry,* 14 pp. 31-42.	Trust, expertise and knowledge is essential for a therapeutic relationship.<br><br>We should strive for epistemic humility and avoid epistemic injustice.

Step 3: Testing and modifying the ideas/explanations

Q: Do certain aspects during the consultation lead to specific aspects on the maladaptive and adaptive emotion regulation cycles?
Is there a negative or positive?

Emotion regulation pathways		
Adaptive emotional regulation	***Concept***	***Maladaptive emotional regulation***
- Accepting an explanatory model for pain (Toye and Barker, 2012).	**Examination and diagnostic investigations**	- Diagnostic uncertainty<br>- Lack of a thorough examination +/- diagnostic investigations.

<table>
<tr>
<td>- Thorough examination with justifications.
- Accepting of the biopsychosocial model (Bourke et al, 2022; Spink, A., Wagner, I. and Orrock, P. 2021).</td>
<td>How can the examination or diagnostic explanation go wrong, or right?</td>
<td>- Fixed on the biomedical model (Toye and Barker, 2012).</td>
</tr>
<tr>
<td>- Patient centred care.
Setting meaningful goals.
- Active listening and feeling understood.
- Epistemic humility.</td>
<td>Interactions with the clinician
Is there certain interactions that work?

(Buchman et al 2017; Costa et al, 2022; Costa et al 2023; Darlow et al, 2013; Lian et al, 2021; Serbic and Pincus, 2015; Slade et al, 2012).</td>
<td>- Poor patient-clinician relationship.
- Symptoms not legitimised.
- Epistemic injustice</td>
</tr>
<tr>
<td>- Sufficient symptom management.

- Learning to live with pain, whilst maintaining an acceptable self-identity.</td>
<td>Physiological symptoms and pain perception

How does pain perception link and how does clinician interaction impact that in a good or bad way?

(Bourke et al, 2022; Wojtyna, E., Palt, L. and Popiolek, K., 2015)</td>
<td>Fluctuating symptoms/episodic nature of CLBP.
People with CLBP view their symptoms as a threat due to its unpredictable nature
- Self-doubt
- Low pain self-efficacy
- Negative pain perception and its impact on their future.</td>
</tr>
<tr>
<td colspan="3">As above
Bandura’s self-efficacy theory (1994)

Hope is negatively correlated with self-efficacy, explored within chronic musculoskeletal pain (Or et al, 2021).</td>
</tr>
<tr>
<td>- Identify and deconstructing fears
- Supervised (+/- group) exercise.
- Graded exposure.
- Pacing.</td>
<td>Fear of the unknown

How does fear of the unknown increase IU?

What helps or doesn’t help fear?</td>
<td>There is statistically significant link between fear of the unknown and anxiety disorders + depression.

Fear of losing self/identity.</td>
</tr>
</table>

	(Carleton, 2016)	
- Appropriate psychological support.<br>- CBT.<br>- Laughter therapy.	**History of psychological disorders**	= Mental health disorders / depression = leads to maladaptive emotional regulation (Leite et al, 2019)<br><br>Conditions such as: Anxiety disorders, Depression, Perceived stress, OCD, Agoraphobia

Step 4: Identification of part of an idea

The information above outlines:
- Factors that people with CLBP commonly feel uncertain about
- How the patient-clinical interaction can influence the outcome of the patient both positively and negatively.

<u>STEP 4:</u> Iterative process of idea generation

Figure 43: A table outlining the third research question and its line of argument synthesis (steps 1-4)

**Step 1 (the question):** How are the concepts of hope and uncertainty linked?			
**Step 2: Potential explanations**			
**What I searched?**	**Why?**	**Article:**	**Conclusions:**
Intolerance of uncertainty 2020-2024 Google scholar	What is IU?	Sandhu, T., Xiao, B. and Lawson, R. (2023)	• Outlines the concept of IU and underlying mechanisms<br><br>Forms of uncertainty:<br>1st order uncertainty = risk, is incomplete / estimated.<br>2nd order uncertainty = uncertainty about 1st order uncertainty<br>Higher order uncertainty = how frequently 1st order uncertainty changes<br>Structural uncertainty = the number of probabilistic relationships<br><br>Early life stresses may explain the variances in cognitive outcomes and beliefs about uncertainty.
Intolerance of uncertainty AND Hope 2014-2024 Google scholar	How are hope and uncertainty linked?	Demirtas and Yildiz (2019)	• The link between hope and uncertainty is **cognitive flexibility**, but there is limited research to support this direct link.<br>• Individuals who are cognitive flexible are tolerant of uncertainty and able to cope with internal and external stresses.<br>• Hopelessness is negatively correlated with cognitive flexibility<br>• Hopelessness is positively correlated with IU and preserved stress<br>• Cognitive flexibility is negatively correlated with preserved stress<br>• Cognitive flexibility is negatively correlated with IU
Intolerance of uncertainty AND Emotion regulation 2014-2024 Google scholar	How is IU linked with emotion regulation?	Ouellet et al (2019)	• The study explored how IU contributes to worry.<br>• Linked through two aspects of the ERM (negative problem orientation and negative emotion orientation).<br>• Thus provides evidence for an integrated model (IUM and ERM)

Intolerance of Uncertainty AND Emotion regulation Google scholar 2003-2024	How do emotion regulation strategies influence tolerance of uncertainty?	Sahib et al (2023)	Systematic review and meta-analysis. 91 studies.<br>Positive correlation between maladaptive ERS and intolerance of uncertainty<br>Negative correlation between adaptive ERS and intolerance of uncertainty.
3 x quantitative studies from the initial systematic search explored CBT, mindfulness and laughter therapy as treatment interventions for improving hope.	How do we address maladaptive emotion regulation strategies in people with CLBP?<br><br>How do we improve hope?	Abdolghaderi et al (2018)	Mindfulness based cognitive behavioural therapy significantly increased hope (agency and pathway) but had NO effect on pain beliefs<br>Quasi-experimental study. 30 participants. Age: 30-50years. All women. LBP > 6months. Two groups: MBCT 8week course vs Control
		Razavi et al (2022)	To explore the effectiveness of cognitive therapy on hope and pain in women with CLBP.<br>Quasi-experimental study with pre- and post- test design and control group. 20 participants (10 in each group). All women. Chronic LBP.<br>CBT statistically significantly improved hope and self-management abilities and reduced pain.
		Fateme et al (2017)	To evaluate the effect of laughter therapy on hope, self-efficacy and pain control in patients.<br>Semi-experimental study. Pre and post-test design with one experimental group and one control.<br>Convenient sampling. 30 patients.<br>Laughter therapy increased post-test scores of hope and self-efficacy in the experimental group and reduced pain.
Framework for hope	Is there already a Framework for Hope?	Soundy et al (2014)	Systematic review – 10 studies.<br>Hope is severely challenged at times of disease onset, during change or uncertainty. Individuals were overwhelmed by impact on their future.<br>Goals are key – but they must be meaningful.<br>HCPs need to understand the patients' self-identity.

**Step 3: Testing and modifying the ideas/explanations**<br><br>Is cognitive flexibility the key to efficient emotion regulation?<br>Does high levels of cognitive flexibility improve hope and uncertainty?			
**What I searched?**	**Why?**	**Article:**	**Conclusions:**
Cognitive flexibility<br>Referenced in Bourke et al 2022<br>(Citation chasing)	How does cognitive flexibility affect emotion regulation?	Vowles and McMracken (2009)	It is well established that a predisposing factor for stress and depression is hopelessness and this relationship is dependent on the ability of an individual to be psychologically flexible.<br>When an individual is psychologically flexible, they are able to confidently respond and adapt to different situations and are more tolerate of conflict and uncertainty, subsequently enhancing levels of hope.
Cognitive flexibility AND emotion regulation<br>2014-2024<br>Google scholar	How does cognitive flexibility affect emotion regulation?	Gentili et al, (2019)	Psychological Flexibility as a resilience factor in Individuals with Chronic Pain.
**Step 4: Identification of part of an idea**<br><br>- Hope and uncertainty are linked and dependent on how the individual regulates their emotions and their ERS.<br>- A key influential factor is cognitive flexibility, but more research is required into how we improve/address it.<br>- Some evidence to show CBT/mindfulness improves hope but doesn't change pain perception.			

<u>STEP 4:</u> Iterative process of idea generation

Figure 44: A table outlining the fourth research question and its line of argument synthesis (steps 1-4)

**Step 1 (the question):**<br><br>Are there any conditions or intrinsic factors that predispose an individual to be less tolerant of uncertainty?			
**Step 2: Potential explanations:**			
**What I searched?**	**Conditions or intrinsic factors**	**Article:**	**Conclusions:**
Uncertainty AND Cognitive processing<br>Google scholar<br>2003-2024	Fear of the unknown	Carleton (2016)	People with anxiety related disorders or depression have statistically significantly higher fear of the unknown.<br><br>Important to identify 'unknowns' at each level of emotion processing.
Hope AND Depression<br>2003-2024<br>Google scholar	Psychological conditions<br>e.g anxiety and depression	Leite et al (2019)	Systematic review, 7 articles.<br>People with depression have low levels of hope – (proven link across several illnesses, but not specifically LBP).<br>Mental health disorders / depression = leads to maladaptive emotional regulation<br>Conditions such as: Anxiety disorders, Depression, Perceived stress, OCD, Agoraphobia
Uncertainty, Chronic low back pain<br>Management<br>Google scholar<br>2003-2024	Fluctuating nature of LBP	Bourke et al (2022)	Qualitative interviews. Grounded Theory. 9 participants<br>6 sub-themes:<br>The main sub theme was self-doubt in their ability to cope with fluctuating symptoms and its impact on day-to-day coping.

Uncertainty<br>AND Depression<br>2003-2023<br>Google scholar	Depression	Serbic and Pincus (2015)	= Higher levels of depression and disability were found in the group with diagnostic uncertainty, but levels of pain intensity did not differ between the groups.
Pain perception<br>Hope<br>Chronic low back pain<br>2003-2024<br>Google scholar	How does their pain perception effect hope?	Wojtyna et al (2015)	Aim: Exploring mechanisms by which hope affects pain perception.<br>Cross sectional study.<br>150 patients with CLBP.<br><br>Level of state hope depends on their previous experiences of pain and the presence of pain at that moment when they undertake an outcome measure of hope.<br><br>People who had high current or previous levels of pain had significantly lower levels of state-hope than those not in pain.<br>Linked with stress and depression.<br><br>In people who have previously experienced low pain intensity's, hope-state may increase when pain re-occurs. Whereas severe pain switches off state-hope.
**Step 3: Testing and modifying the ideas/explanations**<br><br>Do certain conditions lead to the maladaptive pathway? Do certain conditions enhance pain, fear or pain perception?			
**What I searched?**	**Conditions or intrinsic factors**	**Article:**	**Conclusions:**
Intolerance of uncertainty theory	IU is a distress intolerance	Freeston and Komes (2023).	Contrasting perspective with Carleton (2016)<br>IU is a distress intolerance<br>Somatic error theory of anxiety<br>- Examines uncertainty as a felt sense which arises from cognitive appraisal of an internal feeling.

Citation searching from Carleton (2016)	Fear of the unknown	Hong and Cheung (2014)	Meta-analytic review of 73 articles<br>Explored how six commonly reported cognitive vulnerabilities are associated with anxiety and depression.<br>All 6 had moderate – strong correlations<br>IU was most significant
Citation searching from Carleton (2016)	IU: Theoretical and practice perspectives	Carleton (2012)	
**Step 4: Identification of part of an idea:**<br><br>- Patients past medical history and presence of psychological conditions, neurodevelopmental conditions.<br>- Also influenced by their degree of self-efficacy and pain perception.<br>- Fear or intolerance on uncertainty – ongoing debate			

Q: Do you agree with the terms or labels?

Pain perception and physiological symptoms: These two were merged as the model of 'fluctuating uncertainty' by Bourke et al (2022) was shaped by pain self-efficacy (perceived ability to cope) **and** pain perception.

'Constructing an explanatory model' (the diagnosis/understanding) was added as another key component, as per the model by Toye and Barker (2012).

STEP 5: Expression of the theory and testing the model.

Version 1

Q: Should the process start with an unknown?

There are several key elements (history of psychological disorders, fear of the unknown, physiological symptoms, interactions with the clinician and the examination +/- investigations) that can all be influenced by components that are unknown. Therefore, I envision 'the unknown' as encompassing all of these factors which then would then have an effect on how the individual regulates their emotions and subsequently tolerates uncertainty.

Factor 1: Physiological Signals and Symptoms

Persistency of them overtime – longer could be worse

Ability of symptoms to fluctuate and change

Factor 2: History of mental health disorders and/or experiences

Past mental health disorders or related experience may positively or negative influence the response

Factor 3: F[illegible] unknown

An individual's propensity (trait-fear) to experience fear caused by the perceived absence of info at any level of consciousness or point of processing

[illegible] and understanding of pain

Good pain / Bad pain

Inability to consider suffering

Can you consider how pain is defined

[illegible] with clinicians

Patient and clinician relationship and trust.

Feeling heard / listened to.

Establish meaningful goals

The examination.

Q: Should this move to the centre of the model as the clinician can influence / explore all of the other four components?

The Unknown

"A perceived absence of information at any level of consciousness or process"

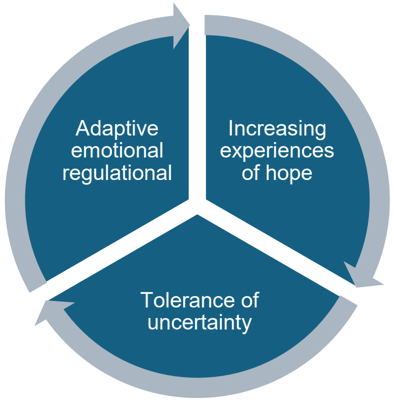

Outcome Perception of uncertainty about the future

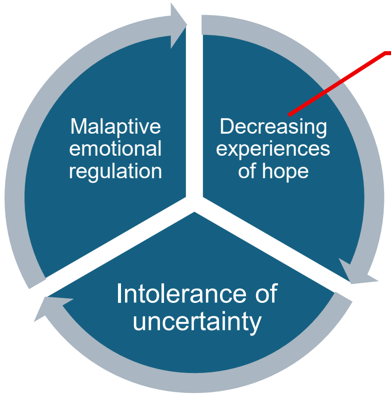

Q: Does the unknown factors lead to different responses? Can we remove the need for the second cycle?

Yes -

See version 4a

<u>STEP 5:</u> Model testing and modifications.

Version 2:

Version 2' did not demonstrate how the psychosocial factors could lead to the adaptive ERS or maladaptive ERS. Articles and further literature searching was undertaken to demonstrate how these different elements fit together.

Q: Do the factors link together? If so how? What is the evidence for this?

Interactions with the clinician was moved to the centre because during the consultation, they can obtain information around their pain perception, concerns/fears, history of emotional disorders etc. **They can influence all factors.**

They are also the most trusted/main source of information –

ARROWS / INTERACTIONS / RELATIONSHIPS:

Q: Do the arrows look right? If not, how should they look? How do the different elements fit together? Are the unidirectional? Or go both ways?

Evidence to support this: the studies that support this?

We manage maladaptive emotion regulation with these treatment interventions/approaches which are based on interactions with the clinician (again, situated at the centre). Those approaches are also based on the model of hope from Toye & Barker (2012).

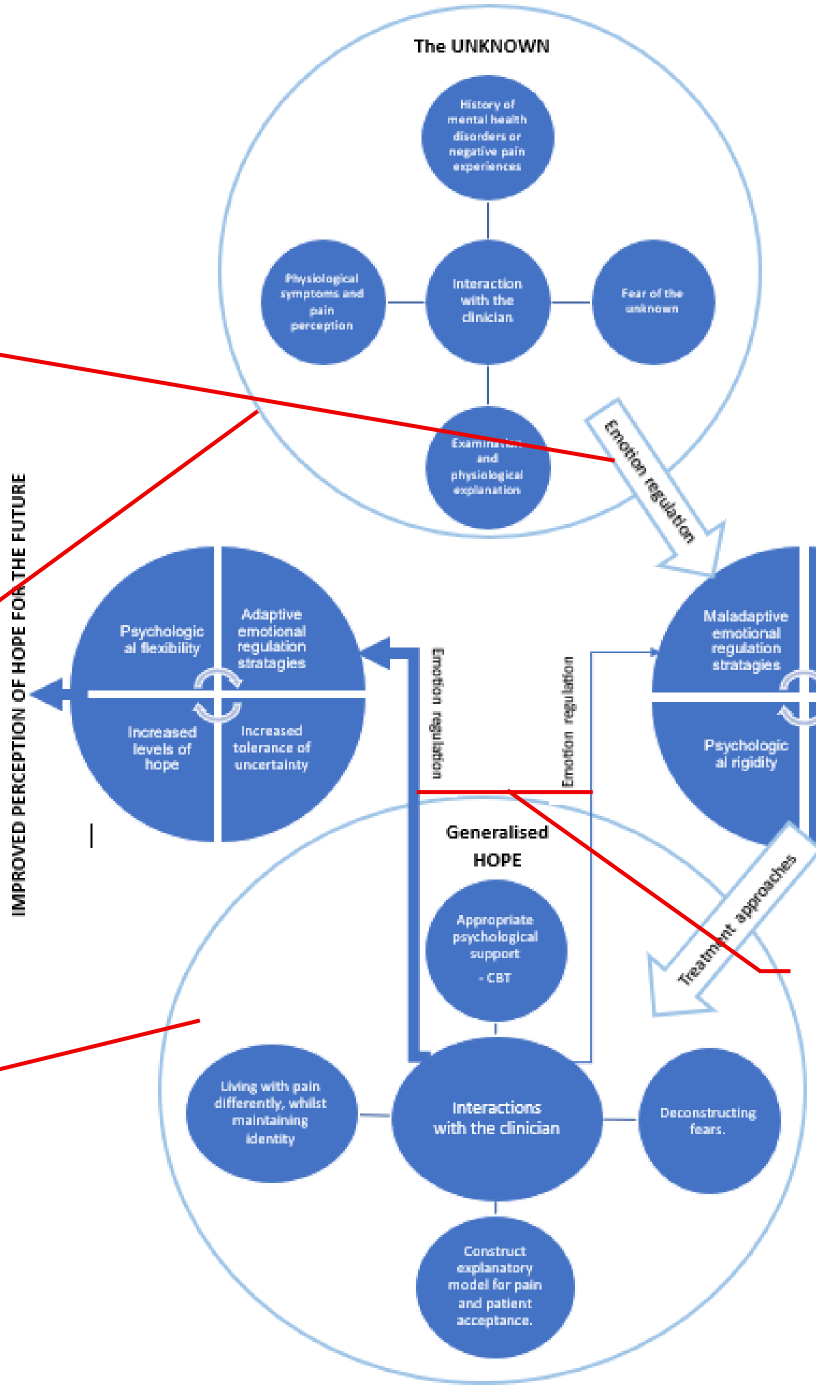

Emotion regulation cycles.

See next page.

Arrows - Justification:
Although most of the time we have a positive influence/outcome (large arrow), sometimes we unfortunately can negatively impact patients' perceptions (smaller line back to maladaptive emotion regulation strategies). ***Literature:*** *Darlow et al (2013)*

Q: Does there appear to be adaptive and maladaptive responses from the studies and is there evidence from the studies of these two cycles? If it doesn't always occur why or when does it not?

The previous/initial cycles:

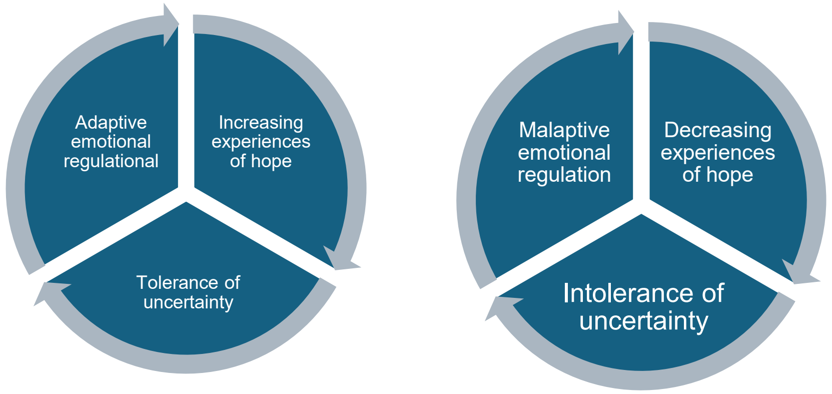

New emotion regulation cycles:

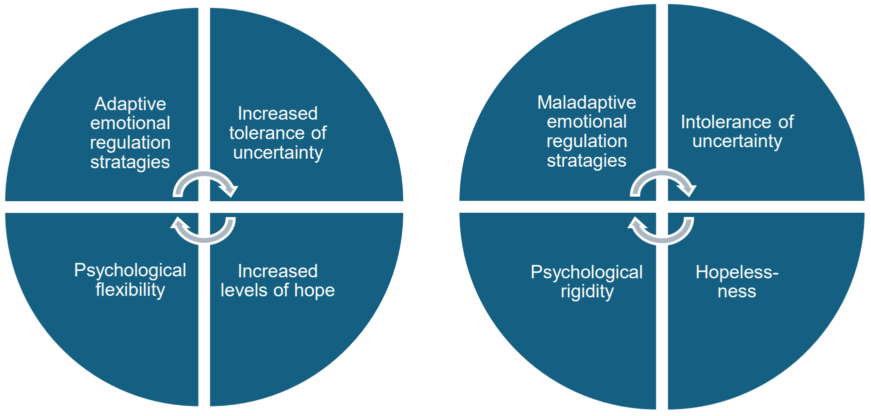

People regulate their emotions in one of two ways:

1) ADAPTIVE:
= TOLERATE uncertainty
= Increased hope
= Cognitively flexible.

2) MALADAPTIVE:
= INTOLERATE uncertainty
= Decreased hope
= Cognitively rigid.

Demirtas and Yildiz, 2019.

Sahib et al, 2023.

Vowles and McMracken, 2009.

<u>STEP 5:</u> Model testing and modifications

Version 3

Q: Is there a different way to present it?

The main issue with this model is that it doesn't show how ALL the 'unknown' factors are interlinked. See version 4a + 4b

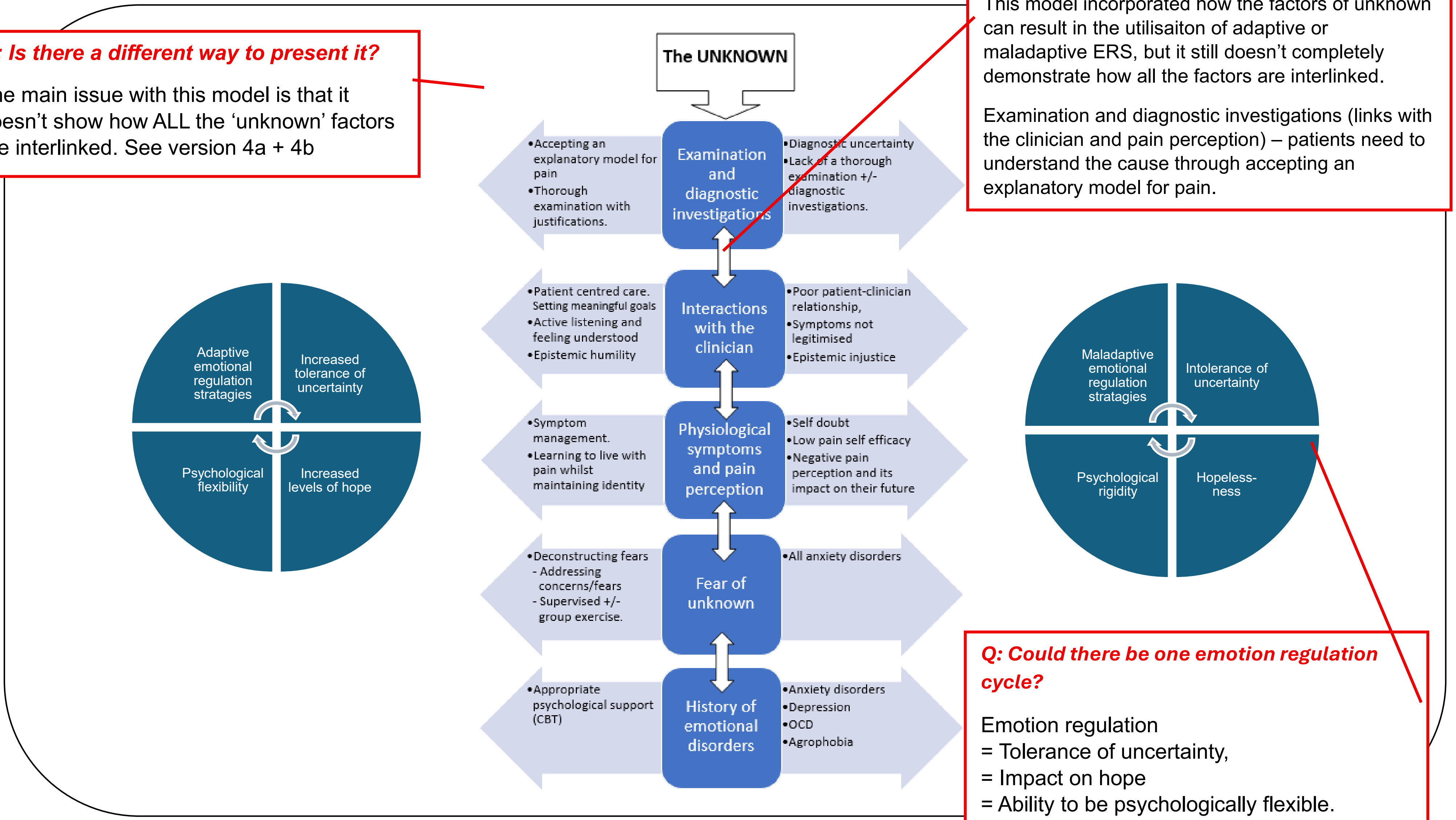

Q: Do the factors link together? If so how? What is the evidence for this?

This model incorporated how the factors of unknown can result in the utilisaiton of adaptive or maladaptive ERS, but it still doesn't completely demonstrate how all the factors are interlinked.

Examination and diagnostic investigations (links with the clinician and pain perception) – patients need to understand the cause through accepting an explanatory model for pain.

Q: Could there be one emotion regulation cycle?

Emotion regulation
= Tolerance of uncertainty,
= Impact on hope
= Ability to be psychologically flexible.

STEP 5: Model testing and modifications

Version 4a

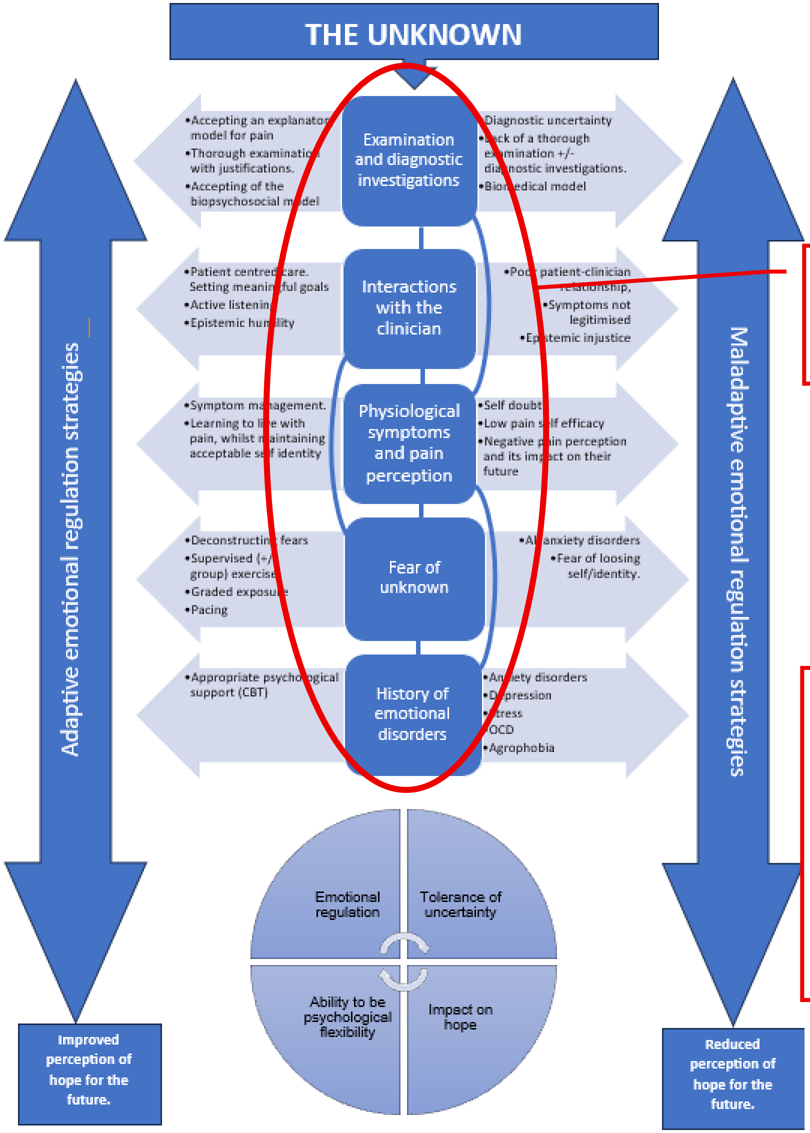

ALL are the factors are interlinked.

Version 4b

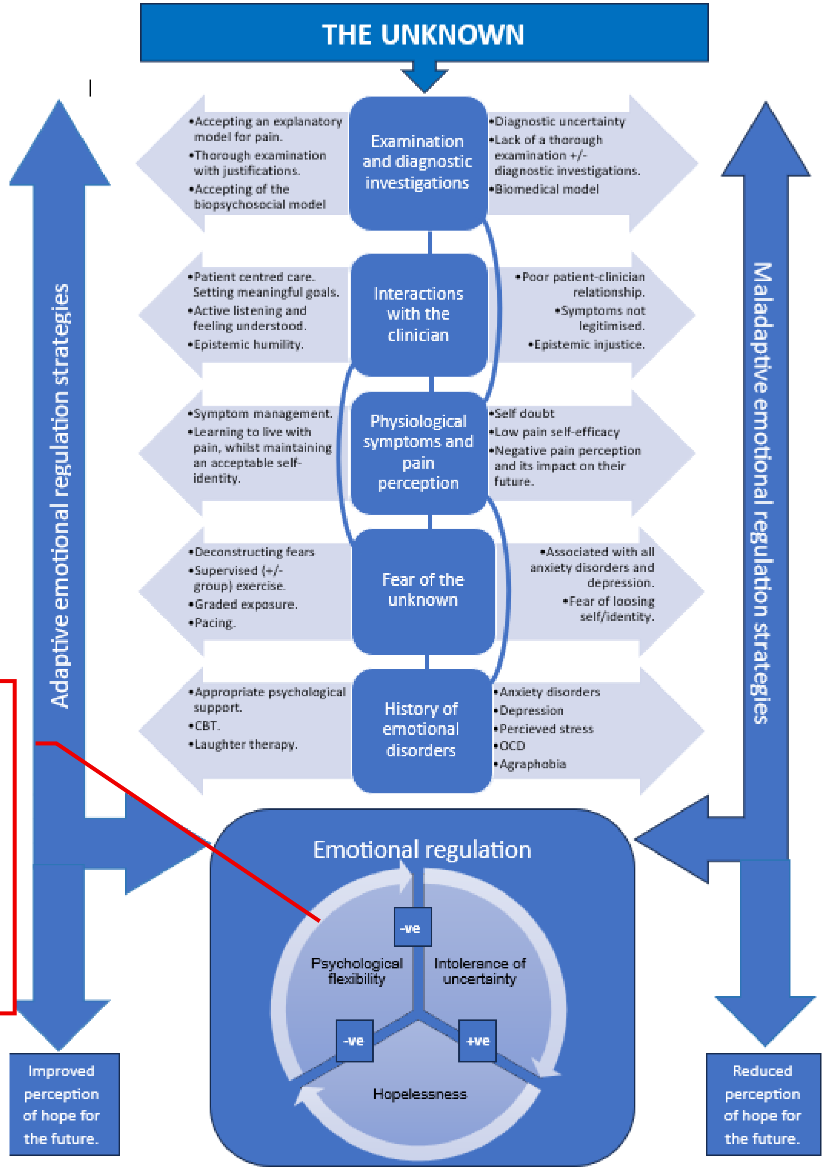

Amendment: Condensed the emotion regulation cycle and added how they are correlated (Demirtas and Yildiz, 2019)

STEP 5: **Model testing and modifications**

Q: Could a simplified process be presented so it can be understood via key stages? See version 5

Version 5

Amendment: Added how they are correlated (Demirtas and Yildiz, 2019)

Q. Are the -ve and +ve always the same within the regulation cycle?

Demirtas & Yildiz (2019) – only one study that explores the relationship between hopelessness, cognitive flexibility, intolerance of uncertainty and perceived stress.

*** Only one study - but the results were statistically significant**

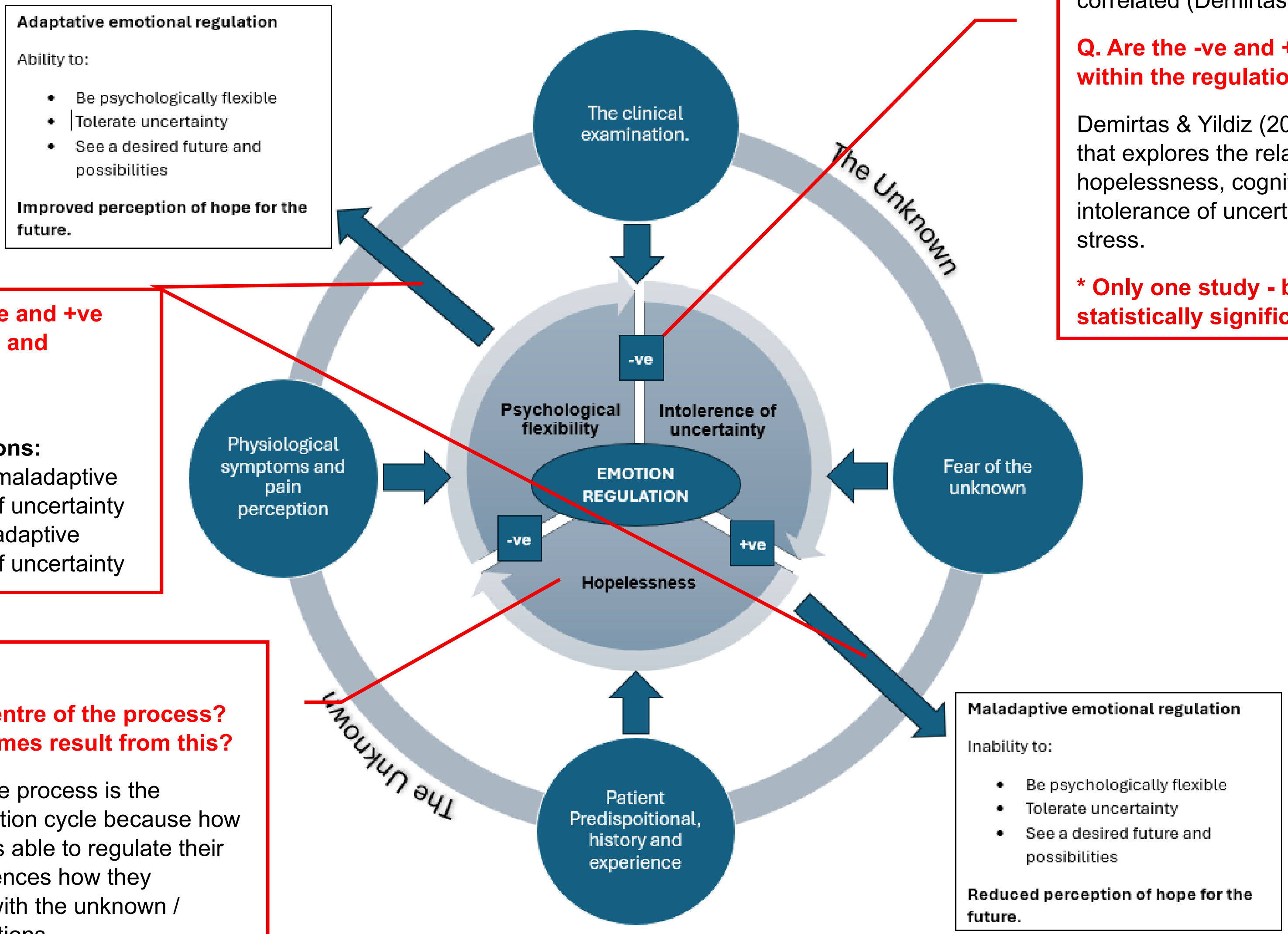

Q. Amendment: Added -ve and +ve correlation symbols for IU and emotion regulation cycle?

Sahib et al (2023)
Well established correlations:
= +ve correlation between maladaptive strategies and intolerance of uncertainty
= -ve correlations between adaptive strategies and intolerance of uncertainty

Amendment:

What is the centre of the process? And do outcomes result from this?

The heart of the process is the emotion regulation cycle because how the individual is able to regulate their emotions influences how they manage/deal with the unknown / uncertain situations.

<u>STEP 5:</u> Model testing and modifications
Version 6:

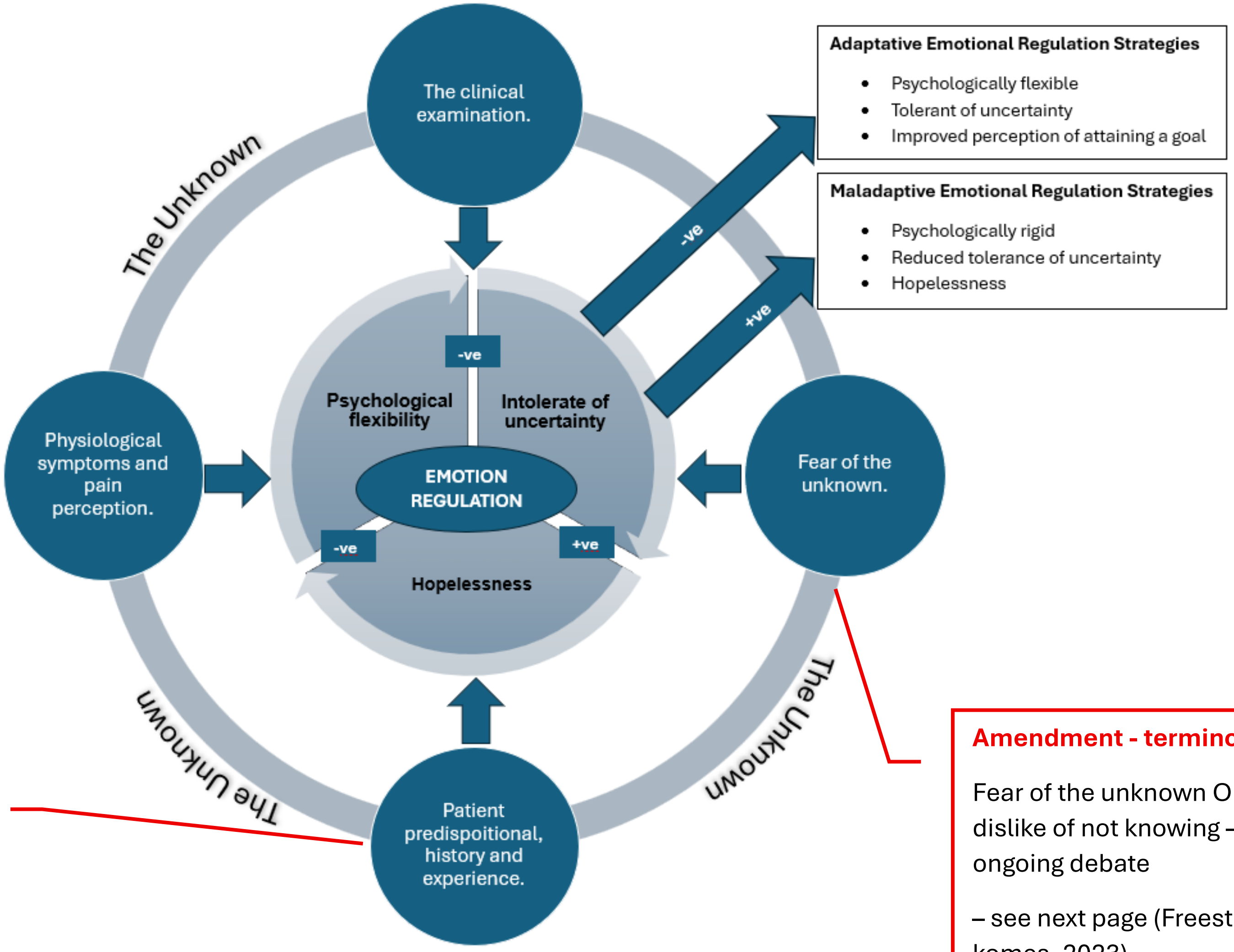

Amendment

See next page / further reading. As opposed to it being an 'unknown' component, it is rather a factor that predisposes the individual to IU and

Amendment - terminology:

Fear of the unknown OR dislike of not knowing – ongoing debate

– see next page (Freeston and komes, 2023).

<u>STEP 5:</u> Model testing and modifications

Version 7a:

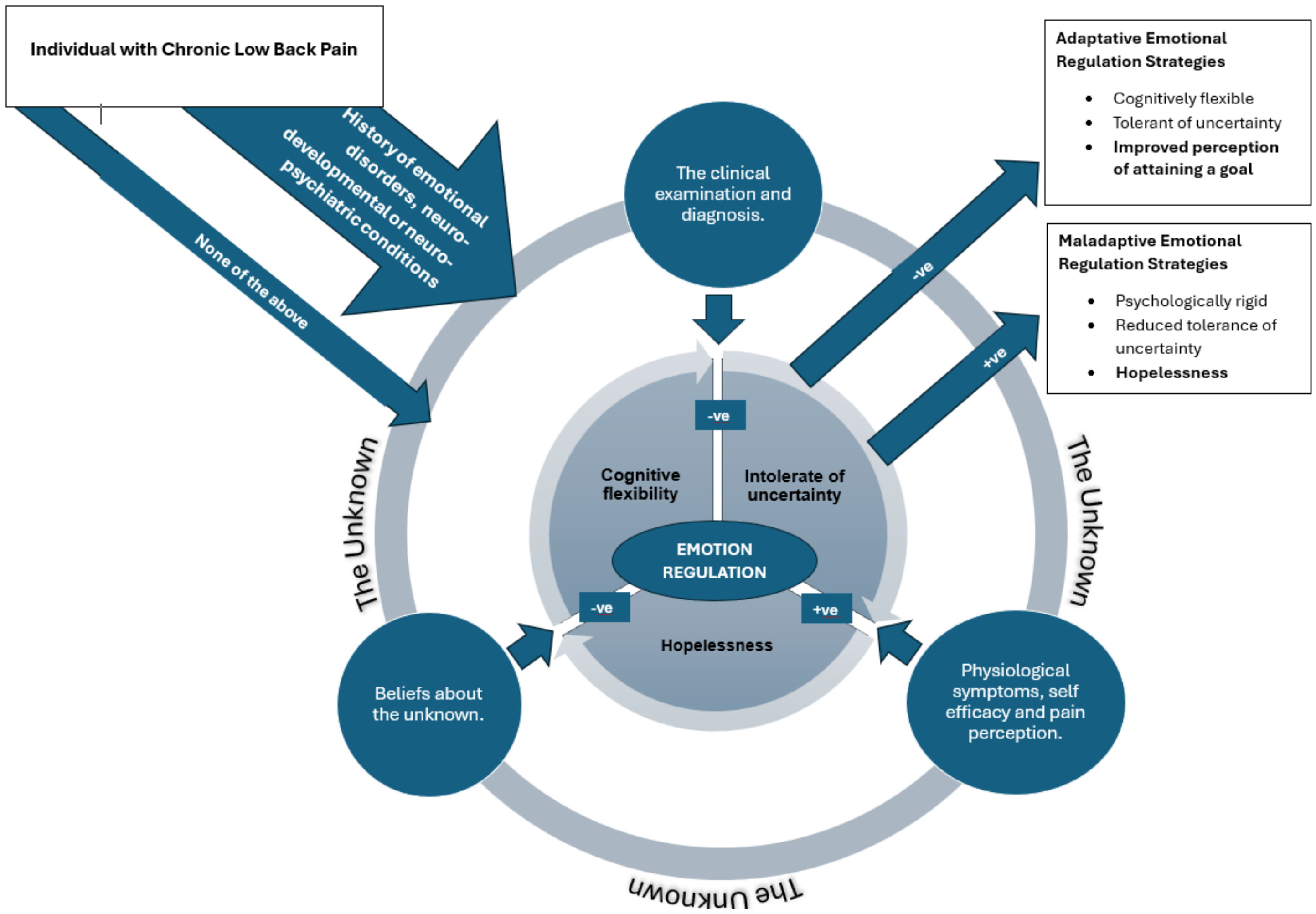

- Final amendments to format, terminology and labels.

<u>STEP 6:</u> Theoretical saturation

FINAL VERSION (7b)

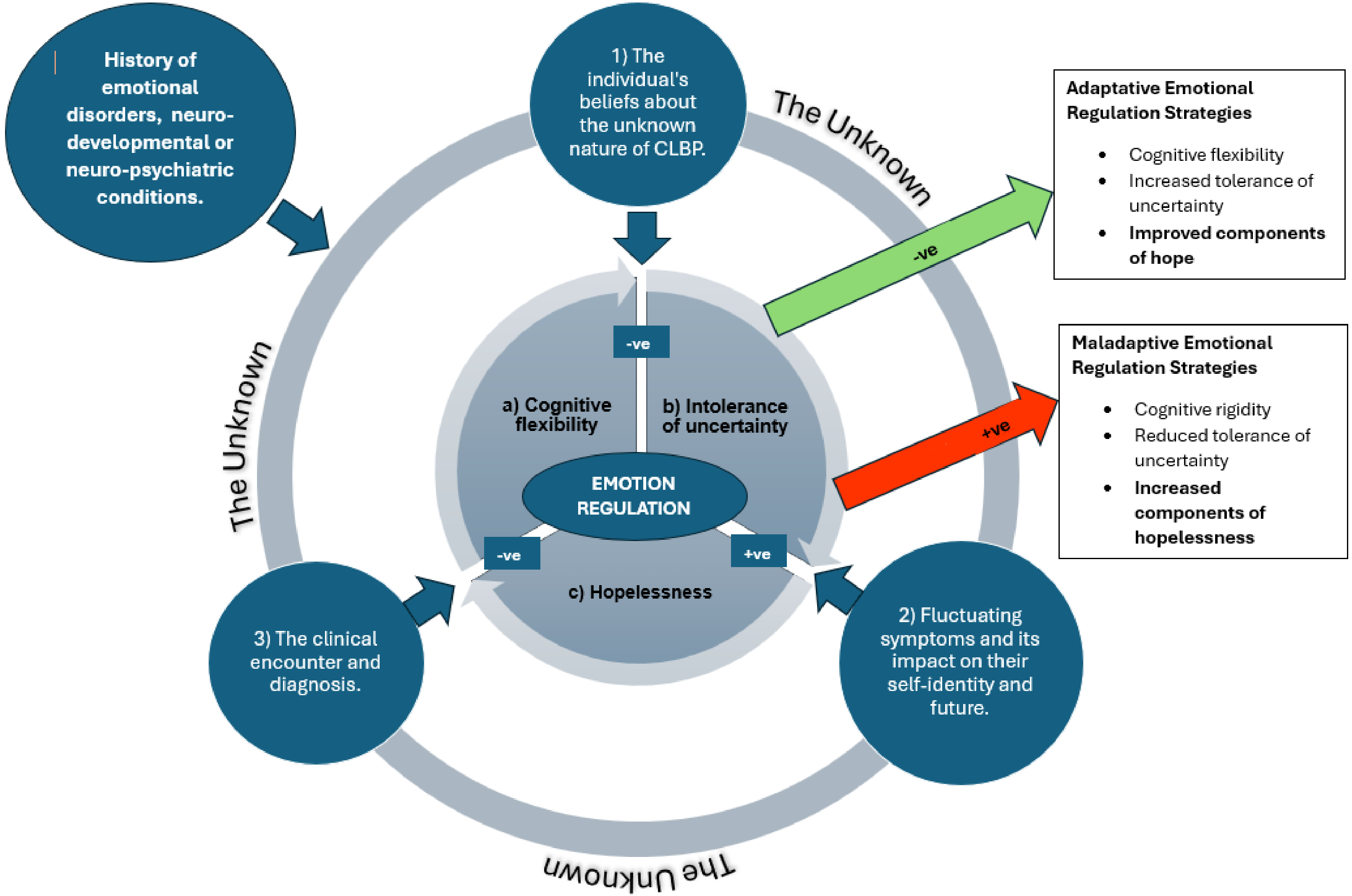

Peer Review Comments and responses

Reviewer 1

The authors present an interesting new approach to analyzing construct relationships that play a role in the context of chronic back pain and should be taken into account in therapy. It seems plausible to me that hope, uncertainty/ambiguity tolerance and emotion regulation play an important role in the modulation of the experience of pain and impairment and are interlinked. Understanding the method of theory development was rather difficult for me as a quantitative researcher with a background in affective/ clinical neuroscience. In my view, the value of the final model lies in the fact that it attaches great importance to emotion regulation. When the concept of emotion regulation is introduced, maybe it would be informative to differentiate between explicit and implicit emotion regulation. On Page 12 you elaborate on the role of interoceptive processes largely influencing implicit emotion regulation involving automatized/ conditioned regulation processes operating outside of conscious awareness.
Author response: thank you for this comment and suggestion we have added a section which elaborates on this.
Overall, some of the connections between the constructs introduced remain unclear to me. I was wondering, for example whether "hope" is really an emotion or related to the cognitive construct of self-efficacy or action orientation (Kuhl). Can "hope" really be considered an emotion as is seems to entail several cognitive-evaluative processes?
Author response: across the literature hope as been identified with 5 major components one of the components is hope as an emotion. We have provided clarity on this.
In some sections, the line of argument could be more stringent. New complex constructs are constantly being introduced, making it difficult to derive central ideas which may inform future research.
I find it astonishing that the connection between tolerance of ambiguity/ tolerance of uncertainty and internalized attachment experiences has been little addressed in the literature. I think it is plausible that basic trust/secure attachment plays an important role with regard to the central concepts of the model.
Author response: explored and incorporated adult attachment insecurity. Thank you.

Overall, I think that the topic of this article and some of the ideas are very relevant to advancing pain management by addressing uncertainty intolerance and emotion regulation. The methodological approach to theory development is new and interesting.
Author response: Shifted the focus to uncertainty intolerance and emotion regulation.

1. Abstract: Please correct typos in the last sentence.
Author response: this has been updated.
2. Page 2, lines 52-53: „..., it can be central to the destruction of hope, resulting in major psychological consequences such as severe depression or even suicide." This sounds a little drastic. Perhaps the authors could tone it down a little.
Author response: this has been updated.

3. Page 2, lines 55-62: I was thinking whether it may be conductive to understanding the relationship between hope and goal achievement if the definition by Snyder et al. was introduced at the beginning of the paragraph.
Author response: this has been updated.

4. It seems to be closely related to constructs such as self-efficacy and actions vs. state orientation
Author response: this has been added as a limitation in the discussion and an area for further research.

5. Page 3, lines 75-77: How does uncertainty influence the ability to hope? Because the perceived lack of information complicates the definition of goals which makes it difficult to mobilize goal-directed energy.
Author response: added lines 68-71

6. Pages 3-4, lines 98- 110: This section is difficult to understand for readers who are not familiar with social science models of theory formation.
Author response: This has been expanded to aid understanding

7. Page 5, line 178: Grammar
Author response: this has been undertaken.

8. Page 8, line 260: I am not sure whether I missed this piece of information but it did not really become clear to me from Figure 2 and the text how exactly eligibility criteria was expanded.
Author response: This was identified within the supplementary file but has now been moved back into main text to make it clear.

9. Figure 5: Why does the history of emotional disorders influence “The Unknown”?
Author response : Thank you – you’re correct, the arrow should feed into the centre/emotion regulation.

What about personality disorders?
Author response: This has now been incorporated.

What does “-ve” and “+ve” mean? I believe that readers could benefit from figure legend introducing the main tenets of the final model. Please introduce the abbreviations.
Author response: Thank you. This has now been amended.

10. Page 10, lines 357-358: "...providing superficial support for treatment interventions such as Cognitive Behavioural Therapy (CBT)". I am a little confused here as mindfulness plays a major role primarily in third wave CBT approaches, such as Acceptance and Commitment Therapy (ACT) and Dialectical Behaviour Therapy (DBT). Mindfulness is not a central component of second wave CBT. Maybe you could be a little more specific here.
Author response: Reference to CBT has been removed to prevent opening up considerations and a limitation has been added.
11. Page 10, lines 420-422: The various explanations of uncertainty intolerance are not mutually exclusive. Someone who has a very sensitive Behavioral Inhibition System and inadequate self-soothing skills is less tolerant of unpleasant sensations. Maybe you could think about how the different theoretical conceptualizations of IU relate to each other.
Author response: this has been added.

12. Page 10, lines 428-430: To what extent are interoceptive processes dysregulated in people with mental illness? I think that this aspect is not self-evident and needs clarification.
Author response: This has been clarified

13. Page 10-11, lines 434-456: I am missing the common thread in this paragraph. It does not clearly convey how exercise therapy/ graded exposure influences core constructs of the final model and how it helps to restore a sense of identity. The paragraph introduces the complex construct "self-identity" without defining it or without being embedded in a theoretical background and ends by citing research about the impact of CBT on hope and pain intensity.
Author response: Removed/amended

14. Page 13, lines 459-460: Does hope influence pain perception or is it the other way around? The study by Wojtyna et al. found that hope is influenced by experiences of pain and pain in the present moment. If the study describes associations rather than causal relationships, please formulate the statements accordingly.
Author response: Amended

15. Page 13, lines 469-470: Is CLBP really "poorly" understood? Maybe you could phrase your ideas behind this expression differently. Self-efficacy and self-identity are being mixed up somehow
Author response: Re-phrased

16. Page 14, lines 501-502: Is cognitive flexibility really a component of emotion regulation or does it result from adaptive implicit and explicit emotion regulation? Others would argue that cognitive rigidity would result from the inability to access brain systems supporting the generation of new self-congruent goals (compare Kästner and Petzke (2024). Personality systems interactions theory: an integrative framework complementing the study of the motivational and volitional dynamics underlying adjustment to chronic pain. Front. Pain Res.).

Author response: Incorporated reference, thank you

17. Page 14, lines 509-510: There is a recent meta-analysis summarizing the evidence from randomized controlled trials investigating the efficacy of Acceptance and Commitment Therapy for chronic pain. Maybe you want to cite this: Ma et al. (2023). The Efficacy of Acceptance and Commitment Therapy for Chronic Pain. A Systematic Review and Meta-analysis. The Clinical Journal of Pain.
Author response: Incorporated reference, thank you

Reviewer 2
Introduction

1. Page 2, line 49, Define what ERS is.

   Author response: This was completed – as per researcher 1 above

The introduction is unsatisfactory, as it fails to discuss important concepts such as the distinct mechanisms underlying different types of CLBP (nociceptive, neuropathic, and nociplastic pain). The claim that the majority of CLBP cases lack identifiable structural causes is unsupported by data. Moreover, the rationale for focusing on hope and uncertainty over other psychological constructs—such as anxiety, depression, or knowledge deficits—remains unclear.
Author response: This has been added – page 2 / lines 36-43
The introduction also provides no explanation of how these factors affect treatment outcomes or recovery. The definitions of hope and uncertainty specific to CLBP are vague; for example, is "hope" related to pain improvement or the identification of a pain cause? Similarly, what does "uncertainty" encompass in this context? Additionally, while emotional regulation appears central to the proposed framework, the authors fail to introduce or systematically review this concept. This omission undermines the theoretical model and creates a confusing and weak rationale.
Author response:: This is now covered.

Methods

1. The exclusion of participants over 70 years old is unconvincing.

Author response: Searching was undertaken again and blind by a second reviewer and this has been removed.

The cited reference is outdated from 10 years ago and does not reflect current data.
Author response: A sentence has been added

Furthermore, the review does not suggest that CLBP prevalence decreases in this demographic. Instead, it indicates consistent prevalence among those over 65. Excluding older participants diminishes the study's inclusivity and relevance.
Author response:: as above this has been removed.

2. The authors must explicitly describe the inclusion criteria for selecting articles about uncertainty in the methods section.
As above: Searching was completed again by a second reviewer and this detail added.
Figure 2 should complement the written content rather than leaving readers to interpret it independently.
The idea generation process described in Figure 2, step 4, appears incomplete and arbitrary. The premise of question #1—What are the key psychosocial factors that people with CLBP commonly report that they are uncertain about?—is unclear. Most patients are uncertain about the causes and recovery of their pain, making the focus on specific psychological factors less intuitive and harder to justify.
Author response: This has been removed.

4. The exclusion of optimism from the search strategy requires explanation.
Author response: Thank you for this comments. We have identified this and drawn on research that considered it a similar but different concept. For instance from the added TenHouten article it is stated that "Hope is not the same as optimism that things will work out for the best, but a belief that some things are working towards because they are right, regardless of how they turn out" Optimism is a secondary emotion of made up of anticipation and joy-happiness.

5. The PRISMA diagrams for both hope and uncertainty raise questions about validity and reliability, with only 6 articles on hope and 3 on uncertainty included out of 225 initially identified. This low inclusion rate raises concerns regarding whether the appropriate search strategy is applied.
Author response: We agree and have completed re-done the searching with two blind reviewers and covidence software to enhance reliability and validity of methods.
Results:
The authors fail to address how sociodemographic factors influence hope, uncertainty, and emotional regulation in CLBP populations. Ignoring these variables weakens the proposed model (Figure 5) and diminishes the depth and applicability of the discussion.
Author response: A limitation has been added.

2. Figure 5 inadequately explains the interplay between hope, uncertainty, and emotional regulation strategies. The relationships among components are unclear, and the model lacks specificity regarding how these factors yield adaptive or maladaptive outcomes. A revised figure with clearer connections and explanations is necessary.
Author response: A revised figure has been undertaken.
3. The review does not provide a detailed analysis of the 9 included articles, despite their centrality to the framework. The methodologies, findings, and relevance of these studies should be thoroughly discussed to establish credibility.
Author response: This has been considered and updated.

4. Emotional regulation strategies (ERS) are emphasized in the results section, yet ERS was not systematically reviewed. The inclusion of ERS in the theoretical model is unsupported by evidence, further undermining the review's conclusions.
Author response: A third search has been added on ERS and CLBP completed

Discussion

1. The relationship between hope and uncertainty is not novel, and the authors' discussion adds little insight. While the importance of these factors in treatment outcomes is noted, the discussion lacks depth regarding their mutual influence and underlying drivers. Key questions—such as whether uncertainty arises from patients' lack of knowledge about their condition or clinicians' failure to address these factors—are not explored.
   Author response: We have rewritten the discussion

2. The proposed theoretical model is difficult to interpret and fails to provide practical guidance for clinical application. It overlooks critical components influencing the interplay between hope, uncertainty, treatment outcomes, and recovery in CLBP populations. While the authors call for further research, the model's limitations and lack of clarity hinder its utility in advancing understanding or practice.
Author response: We have added implications.

Reviewer 3
Abstract
Must specify that the authors used PRISMA methodology.
Author response: This has been updated.

Introduction
I suggest you start with the full name of CLBP and then continue with the acronyms.
Author response: This has been updated.

Method
- The authors refer to using an ethnographic methodology but carried out a systematic review based on the PRISMA model. The authors must describe this methodology in detail.
Author response: More details have now been added.
- If the eligibility criteria indicate that the studies use "population", the authors should replace it with "sample".
Author response: This has been changed.
- The authors should also explain why was limited the search since 2003.
Author response: All done – to gather contemporary literature
- The exclusion criteria, e.g. excluding grey literature, are not mentioned

Author response: We have included grey literature now as a search.
- In the search strategy, the authors mention that did two systematic searches. The authors should justify why did that.
Author response: This is now explained in the methodology
- The authors should also specify whether the inclusion/exclusion criteria were the same for both systematic searches.
Author response: Thank you this has been updated.
- If the second review is an extension of the first, why didn't the authors decide to expose only the second review?
- The authors must show the PRISMA graph for each review.
Author response: Thank you this has now been added.

- The authors should also specify the information search formula, and write the keywords and their Boolean operators for each of the systematic reviews.
Author response: Thank you this has now been added.

- In point 2.5 on Quality, the authors do not express clearly if did or did not do the evaluation. – Author response: Thank you this has now been added.
- Lines 220-256 has a very long text. The authors should write it in a clear, precise, and brief way. Moreover, the authors seem to be reporting results, so it should not be in the Methods section but in the Results section.
Author response: Thank you this has been changed to introduce the related concept of intolerance of uncertainty in the introduction and integrate points from this section in the results.
- The authors begin the Results section with a proposal of their model but first should report the results found in the search and analysis of articles and then propose the model, thus ensuring a logical and coherent discussion.
Author response: We have added a search output summary and quality apprasia.

The authors refer to the Limitation that the search was carried out by only one of the authors, so the authors did not comply with the requirements of PRISMA.
I consider that major methodological weaknesses prevent an adequate analysis of it.
Author response: Thank you we agree so completed two blind searches again and used a software to enhance the process.

Details of actions taken following Peer Review Process (1)

Third systematic search – Emotion regulation

Figure 45. A table outlining the databases and search terms for the third systematic search.

Database	Search Terms
MEDLINE	‘emotion regulation’ or ‘emotion dysregulation’ or ‘regulation of emotion’<br>AND ‘Chronic low back pain’ OR ‘non-specific low back pain’ OR ‘persistent low back pain’
CINAHL	‘emotion regulation’ or ‘emotion dysregulation’ or ‘regulation of emotion’<br>AND ‘Chronic low back pain’ OR ‘non-specific low back pain’ OR ‘persistent low back pain’
PubMed	(‘emotion regulation’ or ‘emotion dysregulation’ or ‘regulation of emotion’) AND (‘Chronic low back pain’ OR ‘non-specific low back pain’ OR ‘persistent low back pain)
AMED	‘emotion regulation’ or ‘emotion dysregulation’ or ‘regulation of emotion’<br>AND ‘Chronic low back pain’ OR ‘non-specific low back pain’ OR ‘persistent low back pain’
PEDro	‘Chronic low back pain’ ‘emotion regulation’<br>‘Chronic low back pain’ ‘emotion dysregulation’<br>‘Persistent low back pain’ ‘emotion regulation’<br>NB: It automatically uses ‘and’ between words.
Google Scholar	allintitle: “emotion regulation” AND "chronic low back pain".
ScienceDirect	(“emotion regulation”) AND (chronic low back pain OR persistent low back pain OR non-specific low back pain) in title and abstract.

Limitations:

- Search mode: Boolean/Phrase
- Publication date: 2003-2025
- Language: English
- Search field: Abstract.

Inclusion criteria:

- Population: adults (18+) with chronic low back pain (>3months)
- Used an outcome measure or discussion of emotion regulation in the results section of the abstract.

Figure 46: The systematic searches were repeated for all three concepts – blind, by both authors using Covidence software

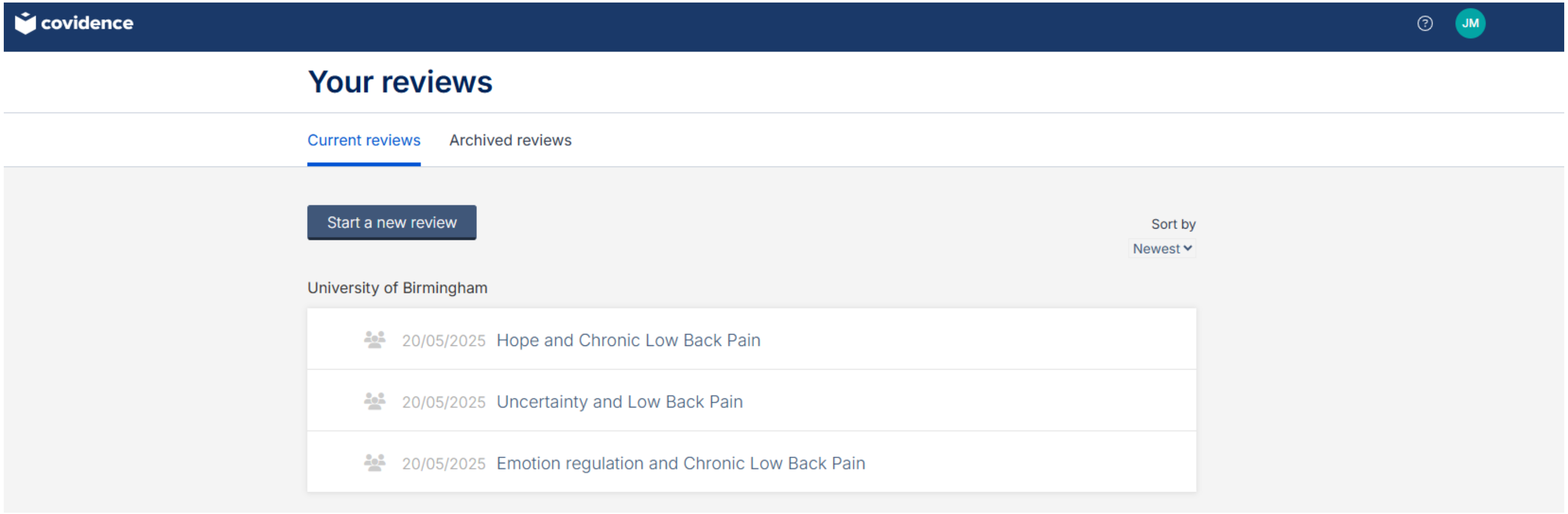

Figure 47. Extraction list Systematic Search 1 - Hope and CLBP :

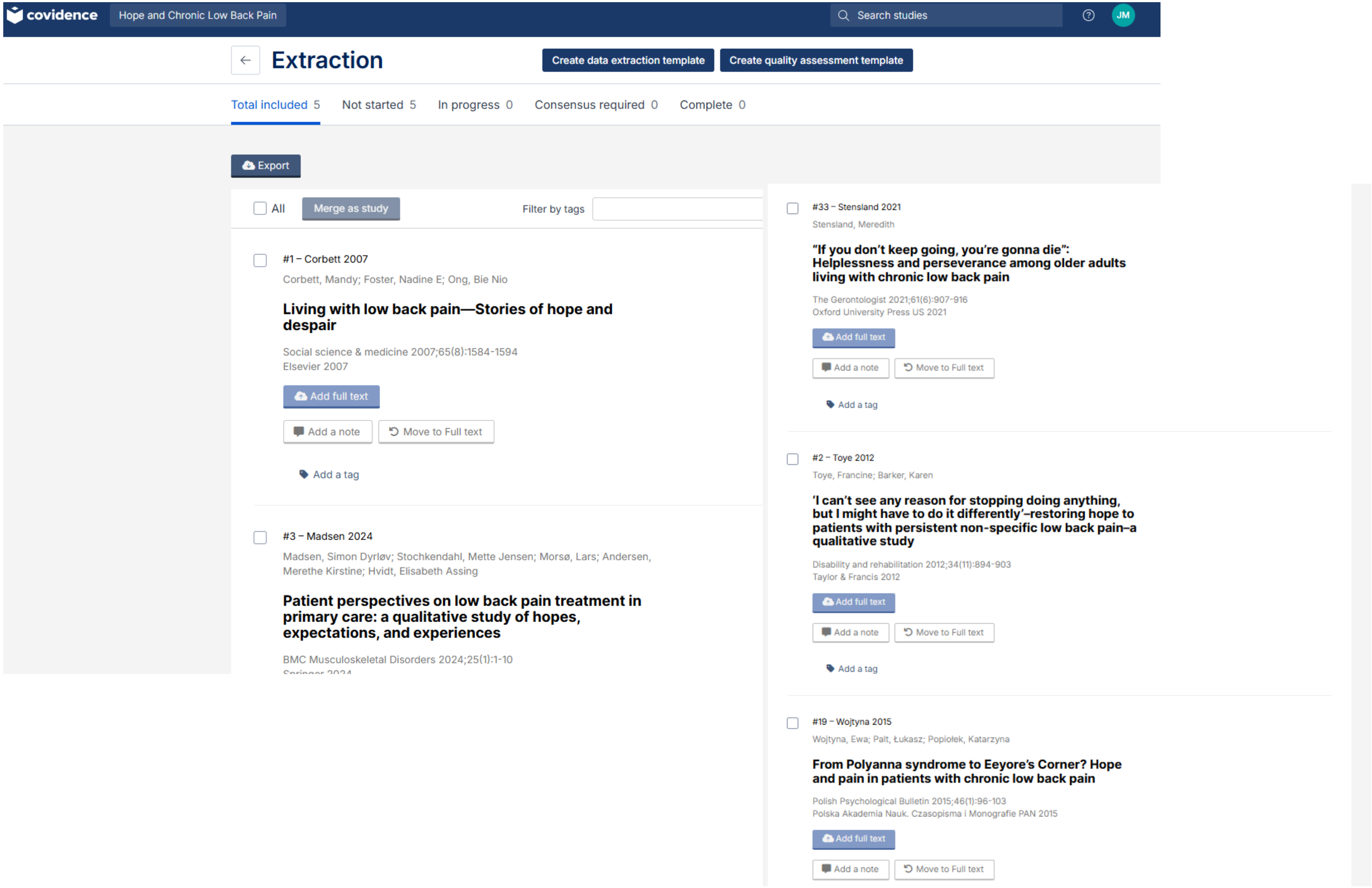

Figure 48. Extraction list Systematic Search 2 - Uncertainty and CLBP:

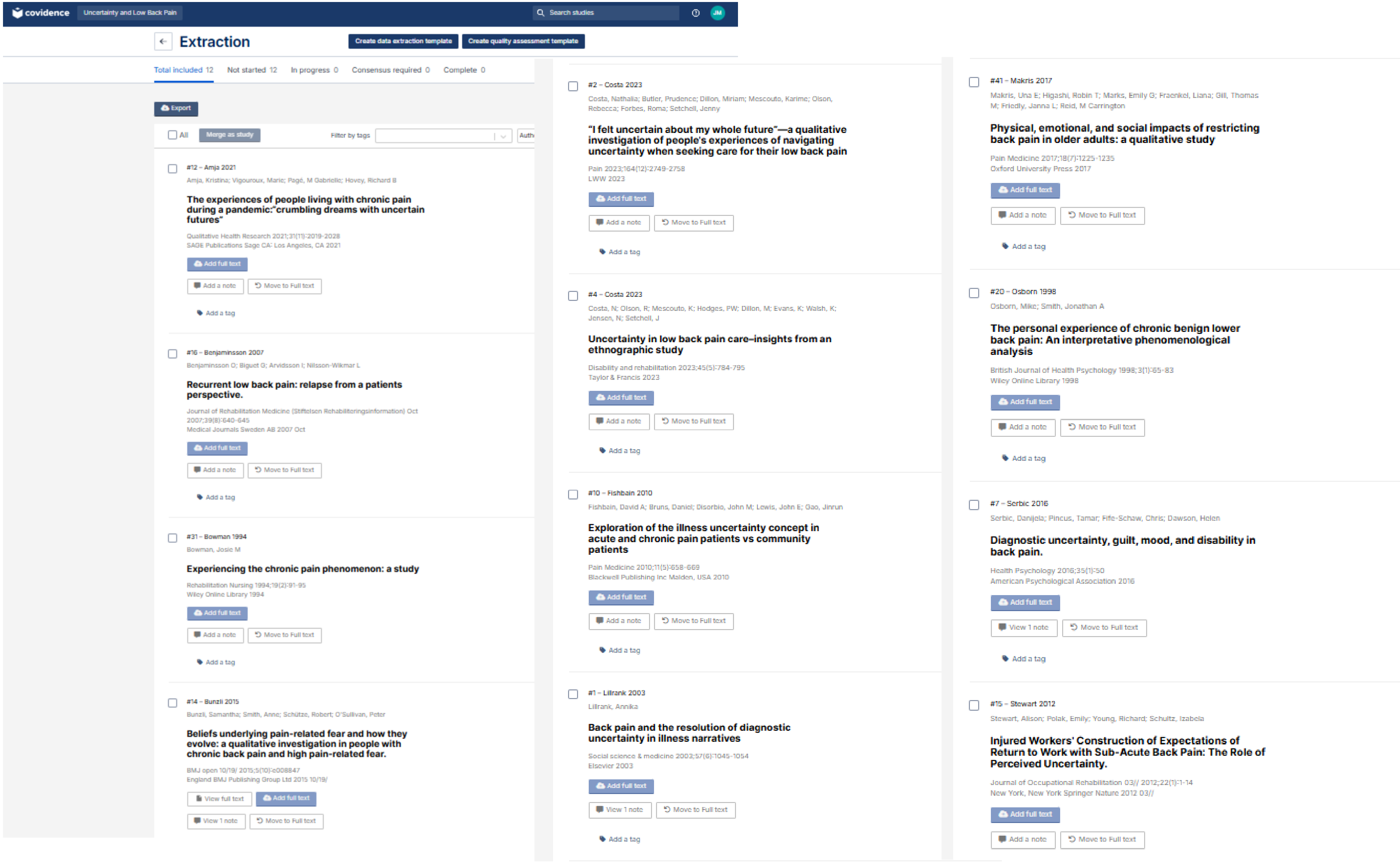

Figure 49. Extraction list Systematic Search 1 – Emotion Regulation and

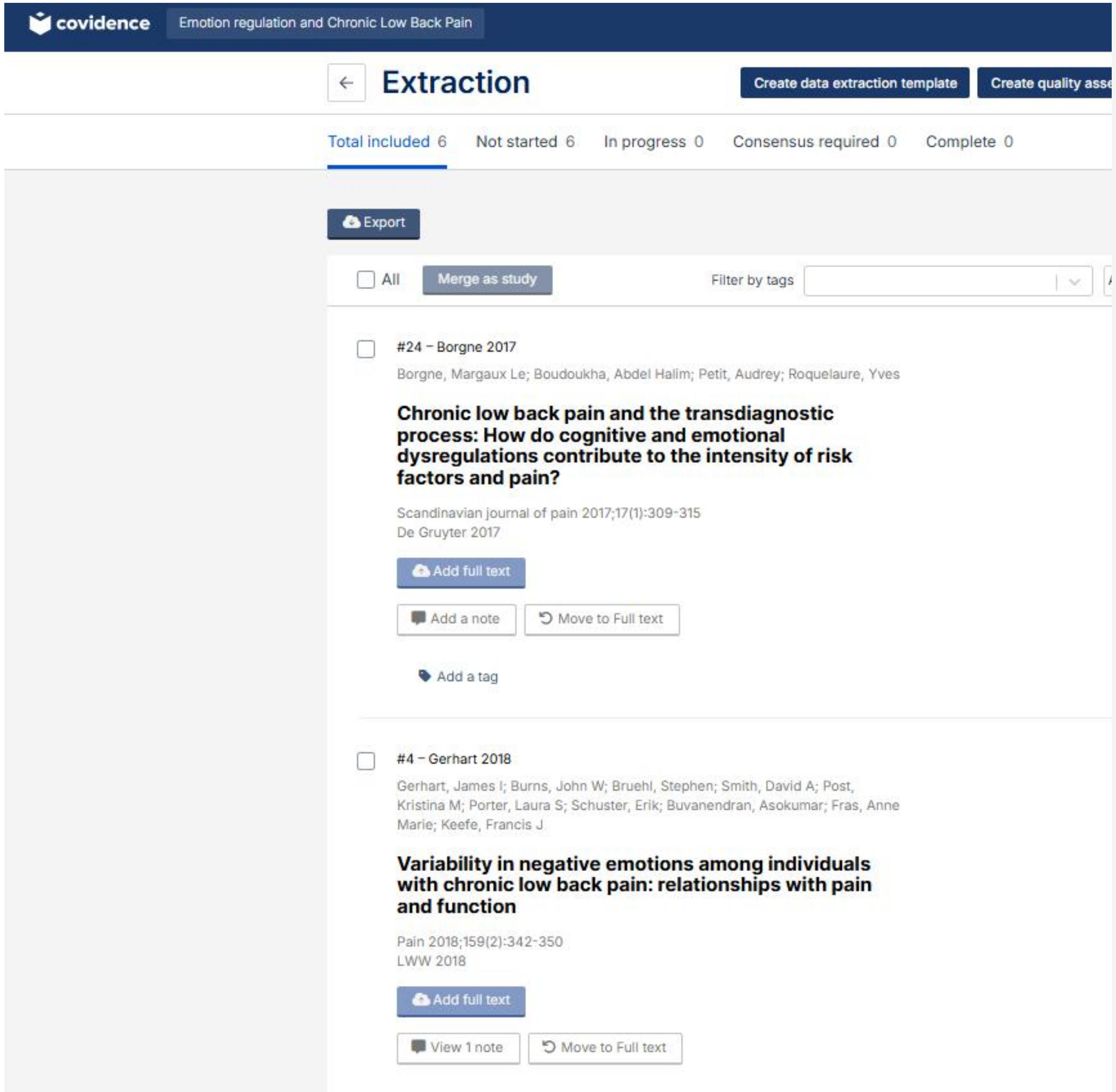

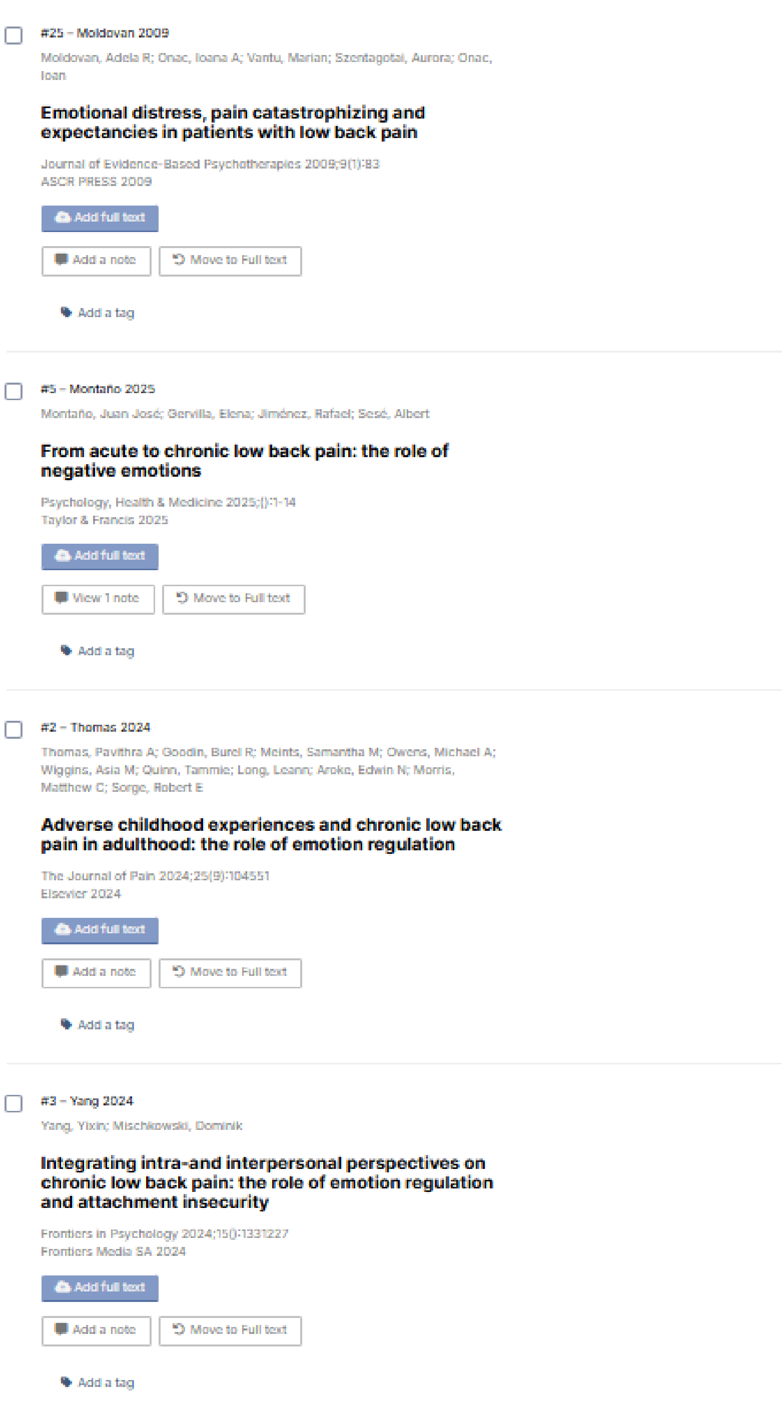

<u>STEP 2 - reviewed:</u> Immersive reading, coding and exploring how the studies relate.

Figure. 50

	**First order coding**<br>(Participant verbatim quotes):	**Second order coding**<br>(Study author reported findings)	**Third order constructs**<br>(Meta-ethnography interpretation)
**Article 3. (Hope)**<br><br>Stensland, M. (2021)	[illegible]<br>• "There's nothing. I would get my hopes all up and think, 'Well, this is gonna do it. This has got to do it.' Nothing. Nothing ever did. "<br>• "I wish it'd go away. Period, but no, it won't—it will not go away. Nothing I try works."<br>• "I have never in my life felt as helpless and as hopeless as I do right now."	Feeling helpless because nothing works.	Treatment failure
	• "I think it's easier to get in a funk [when in pain], and then when you're in a funk, the pain seems worse. "<br>• "I know it's not gonna get better. That's depressing."<br>• "Here we go again with the darn back and then that makes me more depressed. And the depression makes the back pain worse. It's just the cycle.	Feeling down and depressed.	Negative emotions
	• "I felt like right then if I had a gun, I think I would have shot myself. It was just hurting so bad and now they tell me, 'We can't help you.'"<br>• "I realized that the only way I'm not going to have back pain is to die ."<br>• "Unless they cut my head off [the pain will not stop]. There are times I just wish I weren't here anymore."<br>• "I don't think there's a need for me. I don't necessarily know that anybody would miss me if I were gone."	Distantly wishing for an end.	Negative emotions
	• "I feel that everybody has a cross to bear and maybe this [CLBP] is mine. I mean, I guess, I don't have any other answer for it. It's kind of the way it is."<br>• "I do believe in spiritual healing and to me that would be where you find a peace. Finding a peace with what you have does not mean you've given up."<br>• "If you don't accept it, you'll drive yourself crazy."<br>• "The greatest attribute that older people can attain is to be content with what they have. I feel really blessed even with the pain."	Accepting the reality of my pain.	Acceptance

	•"You just keep going. You, unless you're a very depressed person that's looking for the quick way out, otherwise you just keep going."<br>•"It's sort of mind over matter in a way. You learn how to just do things even with pain."<br>• "If you don't keep going, you're gonna die. I mean that's all there is to it."	The pain stays, I keep going.	Acceptance
**Article 4 (Hope)**<br><br>Madsen et al, (2024)	*Ehm, it's simply because I thought, this needs to be taken care of. Some treatment is needed to... for me to be human, right, and not live on pain medication to make it through the day. [P6, physiotherapy]*<br><br>*I think, sometimes, they close it [diagnostic procedures] down too quickly, like it is too expensive to refer you onwards in the system, like, that's how you could sometimes feel. [P12, chiropractic]*<br>*It might be better in 2 weeks, but it might be even worse and make the situation harder than what was really necessary to begin with. [P17, general practice]*	Theme 1: Something needs to be done<br>- Life disruption<br>- Expectations of clinical assessment<br>- Clinicians' expertise	**Diagnostic uncertainty**<br>- People with CLBP want a clear diagnosis or acceptable explanation for their symptoms.<br>- +/- diagnostic investigations<br><br>**Prognostic uncertainty**<br>- Impact on their self-identity / future
	It's the thoroughness in his examination of my body. That is, he really gets to the bottom of this, completely, and keeps trying. [P8, physiotherapy]<br><br>*I think I have a really good sense of [PT1's] uhm, her professionalism, and... she is good at putting things right... in terms of what I have been thinking [...] she seems very, very competent. [P9, physiotherapy]*	Theme 2: Experiences and emotional responses to the interaction with the clinician<br>- Trust<br>- Personal attributes of clinicians	*Already incorporated within the model. Strengthens theory/model

	**First order coding** (Participant verbatim quotes):	**Second order coding** (Study author reported findings)	**Third order constructs** (Meta-ethnography interpretation)
**Article 1 (Uncertainty)**<br><br>**Benjaminsson et al (2007)**	Always when I have this, it is a reaction to something stupid I've done; I've used my body in an incorrect way. …. When I feel well, it doesn't have anything to do with what I've done, but something I haven't done, that I haven't performed an incorrect movement for example. (*Patient 7*).<br><br>The pain frightened me pretty much, so I was afraid to move and then the pain got worse and worse. I lived my life like a little porcelain doll. It felt like my back would break, by the smallest movement… (*Patient 16*).<br><br>I've had a bad back, I am weak, so I am a bad person. I am doing the wrong thing, I am not capable. The back pain makes me feel bad mentally, like I'm not capable and strong. (*Patient 2*).	**Relapse: an unsolved mystery, a source of uncertainty and self-accusation.**<br>- Constantly looking for a cause & medical solution.	Diagnostic uncertainty
	I try to live life as usual and do what I want despite the pain. …I won't get rid of my pain and avoid…I think that I will do things that will take me to the physiotherapist again. I'll be careless, forget myself and then in 5 years I might become stiffer. You don't get more supple and less injury prone when you are older. I don't know, the body is worn out. (*Patient 4*).<br><br>It's all about a new identity. I've never seen myself as a disabled or handicapped person. I've always been able to do what I want…It's hard to realize that you have to get new goals in life. (*Patient 10*).	**Relapse: an obvious part of life that has to be ignored.**<br>- Trying to live life as usual	**Prognostic uncertainty**<br>- Impact on their future
	The back pain is a way to keep within limits. If I'm not good at staying within limits, then the back pain sets the limits, for instance for how much I can cope with in everyday life. (*Patient 1*).	**Relapse: a reminder to keep within limits, both physically and psychologically**	**Pain perception**

**Article 2 (Uncertainty)**<br><br>**Bowman, J (1998)**	The back pain has helped me to relax and to set my priorities straight. You can't do everything, you have to take the good with the bad, as I say. (*Patient 12*).	**Relapse: an indicator to change behaviour to prevent further relapse.**<br><br>**Seeking non medial relief**	Links with self-efficacy
	[illegible]<br>I'm needing something done for my back and legs, you know. I'm going to give him [the physician] a few days. If he don't come across with something, I'm just going to have to go somewhere and get a doctor [who] will run some tests and find out what the problem is.	**Seeking a cause**	**Diagnostic uncertainty**
	First he sent me to [a hospital] for an MRI. He sent me over there to Dr. B.—he's the one who put the injection in there—and after that my hip started hurting, still hurting. And then he sent me home. He sent me back to Dr. S. and he referred me to the pain clinic. I don't think none of them knows what it is.	**Seeking medical help**	**Clinician encounter**
	There's a certain amount of pain that you're going to have to live with. I realize that. I know that I'll never be completely pain-free. I don't expect that. It's just something I'll have to live with the rest of my life. Just to be able to cope with it is what I want.	**Accepting the pain**	**Acceptance**

**Article 3 (Uncertainty)**<br><br>**Bunzli et al (2015)**	**Main theme:**<br>The pain experience did not make sense.		**Diagnostic uncertainty**
	When my back was completely bad, 10/10 pain, I got scared then, thinking what is going to happen am I going to be in a wheelchair and yeah when it gets that bad and I can't walk then that is when I do get really scared. (044, line 233)<br><br>You just don't want to live with that sort of pain…everything just stops you are just so consumed with that pain level. I am writing, I am really distressed and can't cope. It is just not good and so I avoid it. (029, line 350)	**THE PREDICTABILITY, CONTROLLABILITY AND INTENSITY OF PAIN**<br>The experience of pain as unpredictable, uncontrollable and intense made it threatening.	**Prognostic uncertainty**<br>- Unpredictable symptoms
	I think that is where my hesitation and anxiousness comes from…no no I do not want to blow another disc…so I am just super cautious. (012, line 697)<br><br>If you (feel pain) you panic because you don't know if the pain is going to go away. You don't know if you have gone backwards…I don't want to go back to the original pain. (032, line 311)	**NEGATIVE PAST PERSONAL EXPERIENCES OF PAIN**	**Links with pain perception**
	I guess we all have that fear of the spine…there is something about the back, that fear of my god I don't want to do something to my spine, because if I hurt my spine I am not going to be able to walk, I am not going to be able to mobilise and what if I am an invalid and I can't do anything. (013, line 11)<br><br>You grow up hearing horror stories about back surgeries and how it makes things ten times worse. (012, line 26)	**THE INFLUENCE OF SOCIETAL BACK BELIEFS**	**Influenced by beliefs of clinicians, family/friends**

	It could be my discs but they say not, so I am very confused and that is a big deal for me, that I don't know what it is. (038, line 190)<br><br>When you look at everything that says there is nothing to show, how can you treat it? How can you treat it when tests come back negative? (025, line 427)	**PROCESS OF SEEKING DIAGNOSTIC CERTAINTY**	**Diagnostic uncertainty**
	That's the point that I couldn't understand like I am doing everything they want me to do. I am doing physio, I am moving and trying all this and the pain isn't dying. This is crazy. (010, line 128)<br><br>I've been blown off by everyone and stuff I had sought for myself hadn't really worked. I don't know what to do. (016, line 455)	**REPEATED EXPERIENCE OF FAILURE TO CONTROL PAIN**	**Treatment failure**
**Article 4 (Uncertainty)**<br><br>**Lillrank, A (2003)**	After six months of unsuccessful physical therapy, my therapist suggested an appointment with a specialist she knew… This doctor listened and understood me, and above all he wanted to help me without giving up. He found out what was wrong with me. (31114-1)	**To be finally diagnosed was a great relief**	**Diagnostic uncertainty**
	I also began to believe that I imagined my pain. Even many doctors "helped" me in that [belief]. (31114-1)	**They struggled repeatedly to be taken seriously,**	**Consultation**<br>- **Trust**<br>- **Confidence**<br>- **Epistemic humility-** upholding credibility of

			patients' testimonies
**Article 5 (Uncertainty)**<br><br>**Makis et al (2017)**	"It's stopped me from walking normally, whereas before I walked with a normal gait. I've been slowing down to a crawl." (NYC focus group participant)<br><br>"Well, I don't like to bake anymore because standing for any length of time bothers me. I just can't stand there that long working." (PEP interview participant)<br><br>"I always sleep on my back. If I sleep on my side, I can barely walk when I get up in the morning and I hate sleeping on my back. It's not my usual posture but I have to do it because then when I get out of bed, I can walk." (CT focus group participant)<br><br>"First of all, I don't sleep all night. I go to bed early and I get a couple hours of sleep and the rest of the night I'm awake up and down with my [back] pain." (PEP interview participant)	**Physical impact**<br>- Inability to exercise<br>- Affecting functional tasks<br>- Sleep disturbance	**Prognostic uncertainty**<br>- Impact on their self-identity / future
	"Well I'm sour and maybe I'm nasty, I get mean. My temper goes up and I don't want to talk [to] anybody, just leave me alone. Let me die in peace or die in pain or whatever. Just don't bother me." (PEP interview participant)<br><br>"...I get crabby when everything hurts too much... I give people kind of those sharp answers, short and swift." (PEP interview participant)<br><br>"Fear appears. Is this going to get worse to the point that I can't be mobile at all?" (CT focus group participant)	**Psychological impact**<br>- Sadness and irritability<br>- Fears<br>- Loss of hope	**Links with emotional disorders, hope and fear of the unknown**

	"We have a niece and nephew who invite us all the time to join big functions, and I can't do it anymore. I don't want to be in that position where I have to sit for a long time...It becomes uncomfortable. You don't want to leave a group of people you love constantly like that." (CT focus group participant)	**Social impact**<br>- Isolation<br>- Inability to pursue hobbies	**Prognostic uncertainty**<br>Impact on their self-identity
**Article 6 (Uncertainty)**<br><br>**Osborn & Smith (1998)**	I just keep asking myself why the pain is there and I haven't got an answer. I don't know how I should feel really it's just that I don't think it should be there why should I have it? I would have thought that after all this time it should have eased up and gone away but it hasn't (Linda).<br><br>Well I always thought you had pain to tell you when there was something wrong.	**Searching for an explanation**	**Diagnostic uncertainty**
	When I see all of my friends, I saw one running for the bus the other day I thought Oh my God it's ages since I had a good run or a good walk, you know. So for about 5 minutes I felt sorry for myself, and then I saw somebody else in a wheelchair so you know, I'm not quite as bad as that.<br><br>I just think I'm the fittest because there are 3 girls and I'm the middle one and I thought well I'm the fittest and I used to work like a horse and I thought I was the strongest and then all of a sudden it's just been cut down and I can't do half of what I used to do.	**Comparing this self with other selves**	**Prognostic uncertainty**<br>- Impact on their self-identity / future
	It's like anger building up in you. It's like if you're talking to people you're forever, its as though you've got to try and convince them that there's something wrong with you, that get's you down<br><br>It's quite embarrassing because its not something that you can see and I do feel guilty, I know that my back really does hurt and I'm not making it up and I feel sort of angry that I can't do it and I think well I wish I could just prove to them that my back really is bad and that I really must not do it, because if I do I put myself back weeks (Dottie).	**Not being believed**	**The clinical encounter**

	I just want to be on my own. I can't stand anybody, I'm mardy and I'm mardy with everyone else. You know what I mean. I'd rather just take off upstairs (Mary-Ann).<br><br>If anyone asks me if I am going anywhere, come on, no. Rather than tell them why [the discomfort of pain] I just say I can't be bothered they probably think I'm a bit of a misery it's better than going out with them and spoiling their fun.	**Withdrawing from others**	**Prognostic uncertainty**<br>Social relationships

Repeat Step 4: Iterative process of idea generation

Peer review feedback	Article	Main Findings / Contribution to the model
To what extent are interoceptive processes dysregulated in people with mental illness?	Khalsa et al., (2017)<br><br>Pang et al (2019)	Sensitive neurobiological mechanism – BIS<br><br>In people with emotional disorders (such as anxiety and depression), interoceptive processing is often dysregulated, leading to amplified negative emotional responses and a heightened sense of threat or uncertainty.
The role of emotion regulation in chronic pain.	Koechlin et al., 2018	Evidence for increased pain and distress associated with response-focused emotional regulation strategies (e.g., suppression)<br><br>Mixed evidence for antecedent focused emotional regulation strategies like reappraisal, but more positive<br><br>Depression, anxiety is linked with more maladaptive strategies, identifying the importance of cognitive reapprasial

What are the different forms of ERS?	Braunstein et al (2017)	Explicit / Implicit emotion regulation<br><br>The framework is built on two orthogonal dimensions:<br><br>Emotion Regulation Goal<br>Ranges from implicit (nonconscious) to explicit (conscious).<br>Emotion Change Process<br>Ranges from automatic to controlled.
What is the role of secure attachment with regards to these central concepts?	Milulincer 1998<br>Fávero et al., (2021)<br><br>Krahe et al (2013)	Crucial role in developing effective emotion regulation skills in adulthood.<br><br>Insecure attachment (either anxious or avoidant) is linked to greater psychological distress, maladaptive emotional responses, and a reduced ability to tolerate uncertainty.
Evidence for cognitive flexibility, cognitive rigidity (inflexibility) and cognitive fusion	Vlaeyen & Linton (2012)<br><br>Gildanders et al (2014)<br><br>Palm and Follette (2011)	Hypervigilance towards pain stimuli, which is normally adaptive becomes rigid<br><br>The reasons for this could be due to adverse conditioning for instance associating specific movements with pain directly over time)<br><br>Cognitive fusion is the dominance of thoughts and perceptions dominating behaviour over the other sources of behavioural regulation (Gillanders et al., 2014)<br><br>Cognitive fusion is identified as mediating the relationship between cognitive flexibility and distress (Palm and Follette, 2011)

<table>
<tr><td>Revisit predisposing factors:
- Personality disorders
- Adverse childhood experiences
- Alexithymia</td><td>
Fitzpartick et al (2023)
Thomas et al (2024)
Einstein et al (2014)</td><td>Expanded predisposing factors.
Predisposing factor.
Strong association ACEs & chronic pain
People with alexithymia are 2 x more likely to develop LBP</td></tr>
<tr><td>Revisiting predisposing factors</td><td>NICE (2016;2020)
WHO (2023)
Canadian Spine Pathway
Global CPGs (BMC 2024)</td><td>
<table>
<tr><th>Source</th><th>Assessment Domains</th><th>Recommended Tools</th><th>Imaging Guidance</th><th>Psychosocial Evaluation</th></tr>
<tr><td>NICE (2016;2020)</td><td>History, physical exam, risk stratification, functional assessment</td><td>STarT Back Tool, ODI, RMDQ, NRS/VAS, EQ-5D</td><td>Avoid routine imaging; MRI only if red flags or surgery considered</td><td>PHQ-9, GAD-7; assess fear-avoidance, depression, anxiety</td></tr>
<tr><td>WHO (2023)</td><td>Holistic, person-centred assessment of physical, psychological, social factors</td><td>Exercise, education, physical and psychological therapies</td><td>Discourages routine imaging and use of lumbar braces or opioids</td><td>Emphasizes integrated care and emotional wellbeing</td></tr>
<tr><td>Canadian Spine Pathway</td><td>History, red/yellow flag screening, physical exam, risk stratification</td><td>Structured timelines for reassessment; ODI, RMDQ</td><td>MRI only if neurological symptoms or failed conservative treatment</td><td>Yellow flag screening for psychosocial risks</td></tr>
<tr><td>Global CPGs (BMC 2024)</td><td>History, physical exam, psychosocial risk screening</td><td>Therapeutic exercise, spinal manipulation, acupuncture</td><td>Imaging varies; generally discouraged</td><td>Psychosocial flags used for risk stratification</td></tr>
</table>
</td></tr>
</table>

					unless red flags present	

References:

Braunstein, L. M., Gross, J. J., & Ochsner, K. N. (2017). Explicit and implicit emotion regulation: A multi-level framework. Social Cognitive and Affective Neuroscience, 12(10), 1545–1557. https://doi.org/10.1093/scan/nsx096

Brenning, K. M., & Braet, C. (2013). The emotion regulation model of attachment: An emotion-specific approach. Personal Relationships, 20(1), 107–123. https://doi.org/10.1111/j.1475-6811.2012.01399.x

Canadian Spine Clinical Pathway – Alberta Bone and Joint Health Institute (2025). https://www.albertahealthservices.ca/assets/info/aph/if-aph-prov-spine-low-back-primary-care-pathway.pdf

Fávero, M., Lemos, L., Moreira, D., Ribeiro, F. N., & Sousa-Gomes, V. (2021). Romantic attachment and difficulties in emotion regulation on dyadic adjustment: A comprehensive literature review. Frontiers in Psychology, 12, Article 723823. https://doi.org/10.3389/fpsyg.2021.723823

Gillanders, D. T., Bolderston, H., Bond, F. W., Dempster, M., Flaxman, P. E., Campbell, L., et al. (2014). The Development and Initial Validation of the Cognitive Fusion Questionnaire. Behav. Ther. 45, 83–101. doi: 10.1016/j.beth.2013.09.001

Koechlin, H., Coakley, R., Schechter, N., Werner, C., & Kossowsky, J. (2018). The role of emotion regulation in chronic pain: A systematic literature review. Journal of Psychosomatic Research, 107, 38–45. https://doi.org/10.1016/j.jpsychores.2018.02.002

Khalsa, S. S., Adolphs, R., Cameron, O. G., Critchley, H. D., Davenport, P. W., Feinstein, J. S., Feusner, J. D., Garfinkel, S. N., Lane, R. D., Mehling, W. E., Meuret, A. E., Nemeroff, C. B., Oppenheimer, S., Petzschner, F. H., Pollatos, O., Rhudy, J. L., Schramm, L. P., Simmons, W. K., Stein, M. B., Stephan, K. E., Van den Bergh, O., Van Diest, I., von Leupoldt, A., & Paulus, M. P. (2018). Interoception and mental health: A roadmap. Biological Psychiatry: Cognitive Neuroscience and Neuroimaging, 3(6), 501–513. https://doi.org/10.1016/j.bpsc.2017.12.004

Mikulincer, M. (1998). Adult attachment style and affect regulation: Strategic variations in self-appraisals. Journal of Personality and Social Psychology, 75(2), 420–435. https://doi.org/10.1037/0022-3514.75.2.420

NICE Guideline (2016;2020). Low back pain and sciatica in over 16s: assessment and management. National Institute of Health Care Excellence. URL: https://www.nice.org.uk/guidance/ng59/resources/low-back-pain-and-sciatica-in-over-16s-assessment-and-management-pdf-1837521693637

Palm, K. M., and Follette, V. M. (2011). The Roles of Cognitive Flexibility and Experiential Avoidance in Explaining Psychological Distress in Survivors of Interpersonal Victimization. J. Psychopathol. Behav. Assess 33, 79–86. doi: 10.1007/s10862-010-9201-x

Pang, J., Tang, X., Li, H., Hu, Q., Cui, H., Zhang, L., Li, W., Zhu, Z., Wang, J., & Li, C. (2019). Altered interoceptive processing in generalized anxiety disorder—A heartbeat-evoked potential research. Frontiers in Psychiatry, 10, Article 616. https://doi.org/10.3389/fpsyt.2019.00616

World Health Organisation (2023). WHO guideline for non-surgical management of chronic primary low back pain in adults in primary and community care settings. World Health Organisation, Geneve. https://www.who.int/publications/i/item/9789240081789

Zhou et al. (2024). Global Comparison of Clinical Practice Guidelines – BMC Musculoskeletal Disorders. https://bmcmusculoskeletdisord.biomedcentral.com/articles/10.1186/s12891-024-07468-0

Vlaeyen, J. W., and Linton, S. J. (2012). Fear-avoidance model of chronic musculoskeletal pain: 12 years on. Pain 153, 1144–1147. doi: 10.1016/j.pain.2011.12.009

MODEL (version 8):

As per peer review feedback, this arrow should feed into emotion regulation (not the unknown)

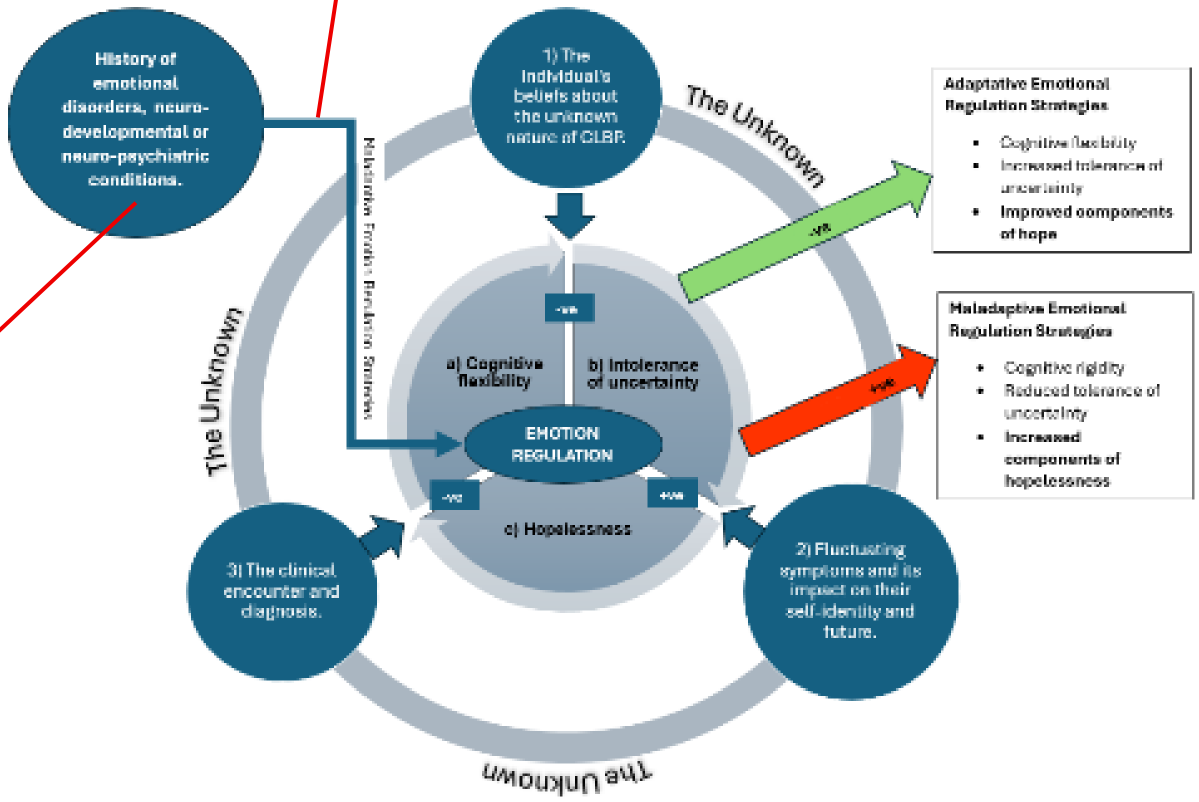

Amended/expanded.
(As per idea generation table)

- Personality disorders
- Alexithymia
- Tendency to worry
- Adverse childhood experiences
- Adult attachment insecurity
- BIS/BAS

MODEL (version 9):

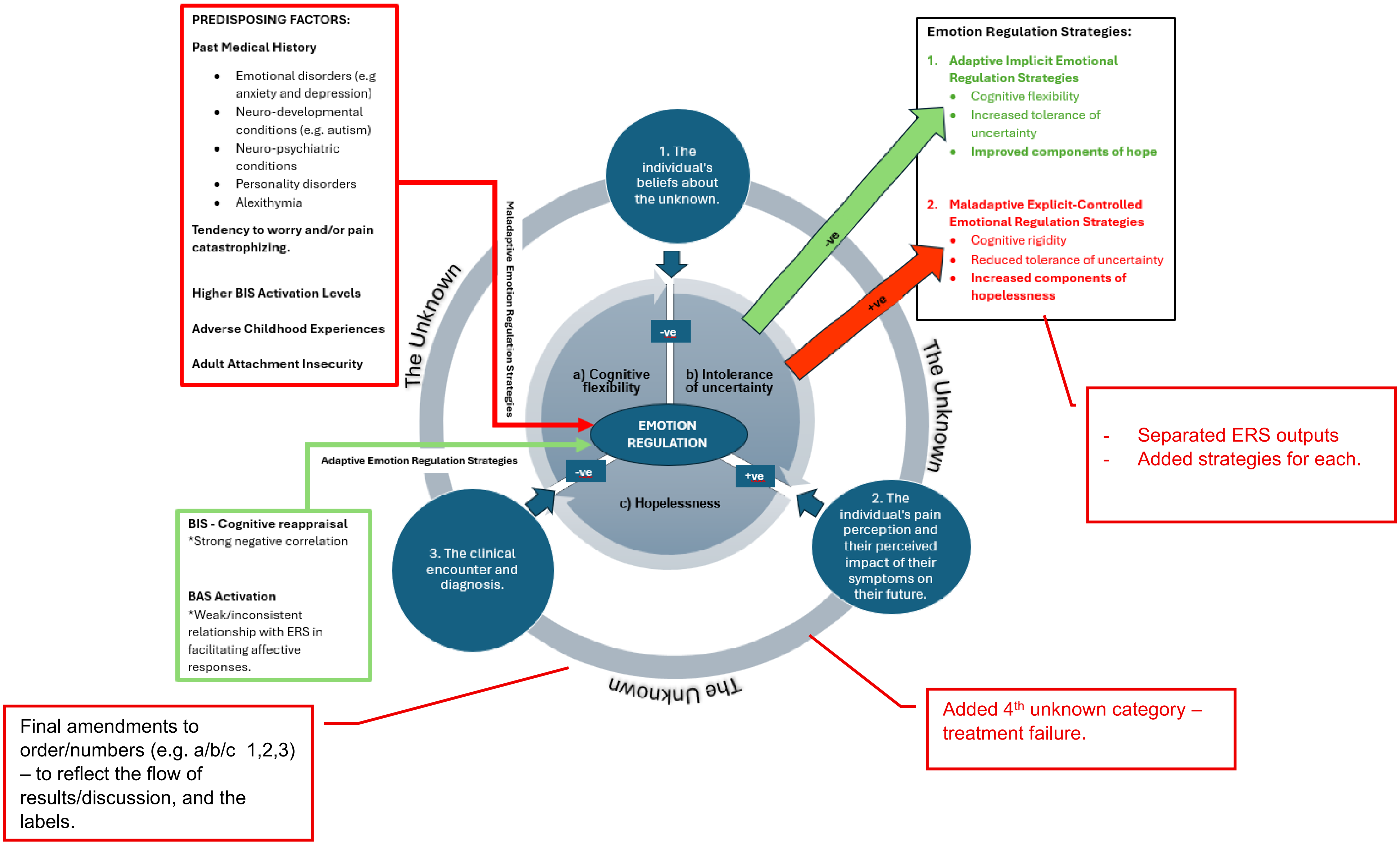

FINAL MODEL (version 10):

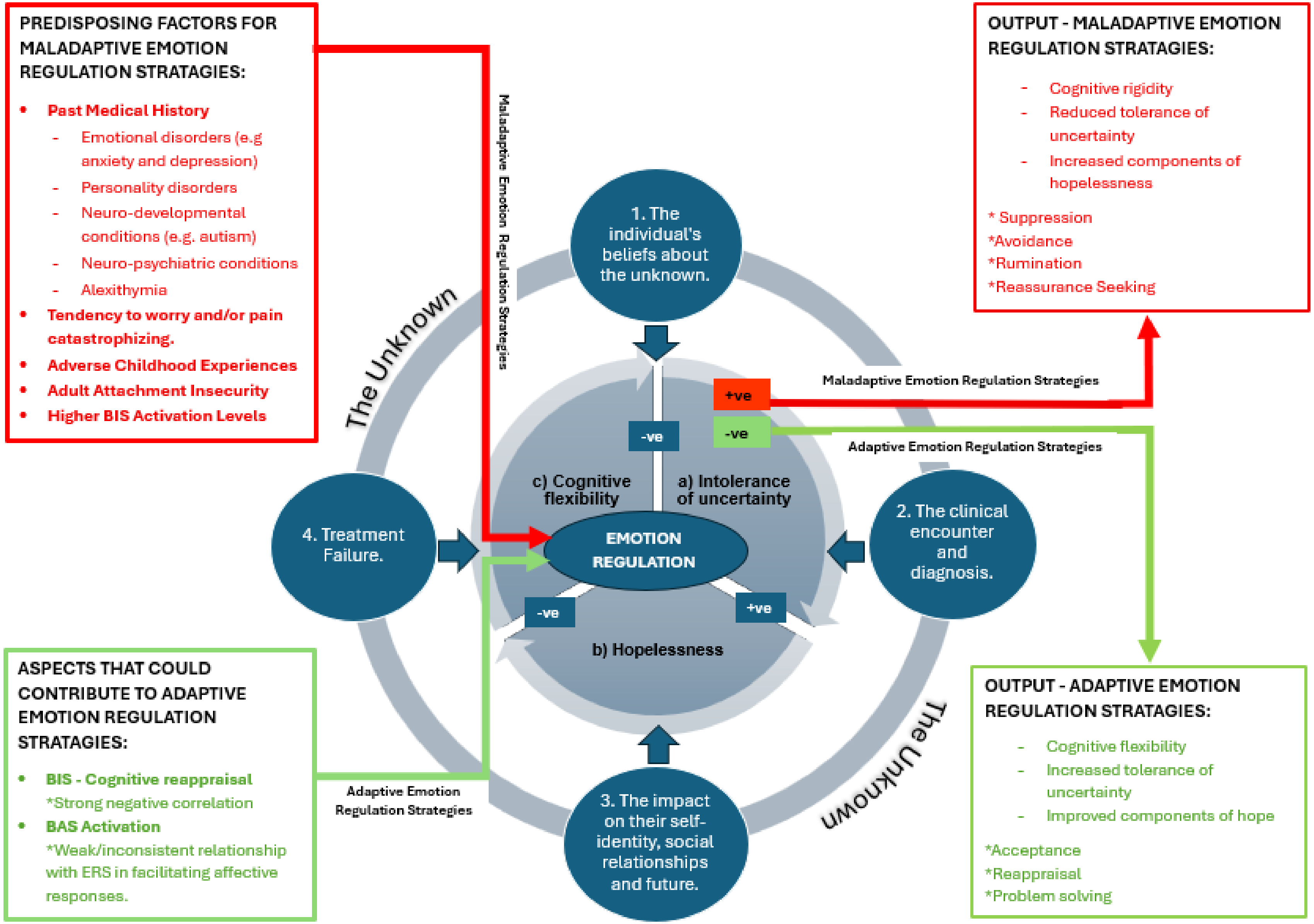

Supplement: Supplementary file 1 [file behavsci-15-01399-s001.zip › behavsci-3750954-supplementary.pdf]
